# Supplementary material for: Natural optical activity as the origin of the large chiroptical properties in π-conjugated polymer thin films
Source: Nat Commun. 2020 Dec 1;11:6137. doi: 10.1038/s41467-020-19951-y (PMC7708482; doi:10.1038/s41467-020-19951-y)
Supplement: Supplementary file 1 — Supplementary Information [file 41467_2020_19951_MOESM1_ESM.pdf]

Natural optical activity as the origin of the large chiroptical properties in π-conjugated polymer thin films

Jessica Wade[1,2], James N. Hilfiker[3], Jochen R. Brandt[4], Letizia Liirò-Peluso[5,6], Li Wan[1,2], Xingyuan Shi[2,4], Francesco Salerno[2,4], Seán T. J. Ryan[4], Stefan Schöche[3], Oriol Arteaga[7], Tamás Jávorfi[8], Giuliano Siligardi[8], Cheng Wang[9], David B. Amabilino[5], Peter H. Beton[6], Alasdair J. Campbell[1,2] and Matthew J. Fuchter[2,4*]*

*[1]Department of Physics, Imperial College London, South Kensington Campus, London SW7 2AZ, UK, [2]Centre for Processable Electronics, Imperial College London, South Kensington Campus, London SW7 2AZ, UK, [3]J.A. Woollam Co. Inc., 645 M Street, Suite 102, Lincoln, NE 68508-2243, USA, [4]Department of Chemistry and Molecular Sciences Research Hub, Imperial College London, White City Campus, 80 Wood Lane, London W12 0BZ, UK, [5]School of Chemistry & The GSK Carbon Neutral Laboratories for Sustainable Chemistry, University of Nottingham, Triumph Road, Nottingham NG7 2TU, UK, [6]School of Physics and Astronomy, University of Nottingham, University Park, Nottingham NG7 2RD, UK., [7]Departament de Física Aplicada, Universitat de Barcelona, IN2UB, Barcelona, 08028, Spain, [8]Diamond Light Source, Harwell Science and Innovation Campus, Didcot, Oxfordshire OX11 0DE, UK, [9]Advanced Light Source, Lawrence Berkeley National Laboratory, Berkeley, California 94720, USA, *e-mail: alasdair.campbell@imperial.ac.uk; m.fuchter@imperial.ac.uk*

Contents

Supplementary Methods: Synthesis of c-PFO and c-PFBT....3

Supplementary Table 1: Mechanisms used to explain observed chiroptical effects....13

Supplementary Table 2: Extracted parameters discussed in this work....14

Supplementary Figure 1: Extracted dissymmetry for ACPCA and CSCP thin films....15

Supplementary Figure 2: Spatially resolved circular dichroism of annealed polymer thin films....16

Supplementary Discussion 1: Mueller Matrix Spectroscopic Ellipsometry....17

Supplementary Figure 3: MMSE data recorded in reflection (R) and transmission (T) for neat, annealed ACP thin films....19

Supplementary Figure 4: Anisotropic dielectric function for neat, annealed polymer thin films....22

Supplementary Figure 5: MMSE data recorded in R and T for annealed ACPCA thin films....23

Supplementary Figure 6: MMSE data recorded in R and T for annealed, unaligned CSCP and ACPCA thin films....26

Supplementary Figure 7: Optical models generated for a monodomain cholesteric-like structure for ACPCA thin films....29

Supplementary Discussion 2 Multi-domain cholesteric model....38

Supplementary Figure 8: Spatially resolved circular terms of ACPCA thin films....49

Supplementary Figure 9: Anisotropic gyrotropic terms for ACPCA thin films....51

Supplementary Figure 10: MMSE data recorded in R and T at three different sample orientations for annealed, aligned CSCP thin films 53

Supplementary Figure 11: Attempting to fit the reflected MMSE data recorded from annealed, aligned CSCP thin films....55

Supplementary Figure 12: Proposed optical model for aligned CSCP thin films....58

Supplementary Figure 13: MMSE data recorded in R and T for annealed, aligned ACPCA thin films....68

Supplementary Figure 14: RSoXS for as-cast and annealed ACPCA thin films....69

Supplementary Figure 15: Radial RSoXS scans....70

Supplementary Discussion 3: AFM measurements....71

Supplementary Figure 16: 2D-FFT of the AFM phase image of annealed F8BT ACPCA films....72

Supplementary Figure 17: Optical micrographs....73

Supplementary Figure 18: *In situ* circular dichroism spectra of ACPCA thin films....74

Supplementary Figure 19: Extracted dissymmetry for ACPCA and CSCP thin films....75

Supplementary Discussion 4: Results of *in situ* CD....76

Supplementary Figure 20: *In situ* circular dichroism spectra of F8BT:aza[P] thin films....77

Supplementary Figure 21: *In situ* circular dichroism spectra of F8T2:aza[P] thin films....78

Supplementary Figure 22: *In situ* circular dichroism spectra of PFO:aza[P] thin films....78

Supplementary Figure 23: Temperature-dependent *in situ* circular dichroism spectra of CSCP thin films....79

Supplementary Figure 24: Time-dependent *in situ* circular dichroism spectra of CSCP thin films....80

Supplementary Discussion 5: *in situ* CD measurements....81

Supplementary Figure 25: Thickness dependence measurements of g_{abs}....82

Supplementary Discussion 6: Thickness dependence measurements of g_{abs}....83

Supplementary References....85

Supplementary Methods Synthesis of c-PFO and c-PFBT

Poly{9,9-bis[(3*S*)-3,7-dimethyloctyl]fluorenyl-2,7-diyl} (cPFO)

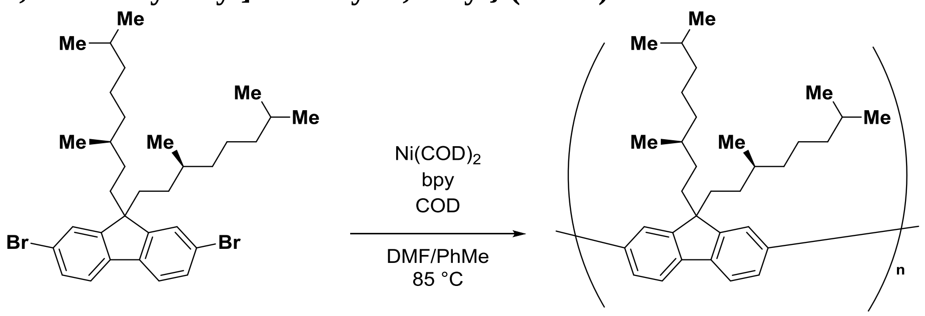

A stock catalyst solution was prepared by adding bis(1,5-cyclooctadiene)nickel(0) (608 mg, 2.21 mmol), 2,2′-bipyridyl (346 mg, 2.22 mmol), and 1,5-cyclooctadiene (325 µl, 287 mg, 2.65 mmol) to a 25 ml Young's tap flask. Vacuum and nitrogen were cycled three times. *N*,*N*-dimethylformamide (DMF, 12 ml) and toluene (8 ml) were added and the mixture submitted to three freeze/pump/thaw cycles. The dark purple solution was then heated to 85 °C in the dark for 30 min. 2,7-dibromo-9,9-bis[(3*S*)-3,7-dimethyloctyl]-9*H*-fluorene (300 mg, 0.496 mmol, 1.00 equiv.) was added to a separate 25 ml Young's tap flask. Vacuum and nitrogen were cycled three times, toluene (8.3 ml) was added and the solution submitted to three freeze/pump/thaw cycles. To this flask was added the dark purple stock solution (6.9 ml, containing 210 mg/0.76 mmol/1.5 equiv. of $Ni(COD)_2$). Gas evolution was visible immediately and the solution quickly turned dark red. The mixture was heated to 85 °C for 6 h, then allowed to cool to room temperature and quenched by the addition of 1 M hydrochloric acid (1.5 ml) and methanol (1.5 ml). Brine (10 ml) and chloroform (10 ml) were added and the phases separated. The organic layer was washed again with brine (2 x 5 ml), dried over $MgSO_4$, filtered, washed with chloroform and concentrated *in vacuo*. The residue was taken up in chloroform (4 ml) and added to cold methanol (100 ml) under fast stirring. The yellow precipitate was filtered, washed with more methanol (5 x 5 ml) and dried under high vacuum overnight to afford 180 mg of yellow precipitate. GPC analysis showed M_n 20600, M_w 41500, PD 2.01. Purification by preparative GPC afforded 54.7 mg of yellow solid, M_n 41400, M_w 54800, PD 1.16.

NMR spectroscopy: ^1H NMR (400 MHz, $CDCl_3$, δ) 7.84 (br d, J = 8.2 Hz, integral: 2.00), 7.76–7.55 (m, integral: 5.18), 2.12 (br s, integral: 4.12), 1.44 (br hept, J = 6.2 Hz, integral: 3.00), 1.32–0.99 (m, integral:

16.98), 0.99–0.88 (m, integral: 1.82), 0.87–0.57 (m, integral: 31.08). ^{13}C NMR (101 MHz, $CDCl_3$, δ) 151.8, 140.7, 140.2, 126.3, 121.7, 120.1, 55.2, 39.4, 37.8, 36.9, 33.1, 30.9, 28.1, 24.9, 22.8, 22.7, 19.7.

2,2'-{9,9-bis[(3*S*)-3,7-dimethyloctyl]-9*H*-fluorene-2,7-diyl}bis(4,4,5,5-tetramethyl-1,3,2-dioxaborolane) (S1)

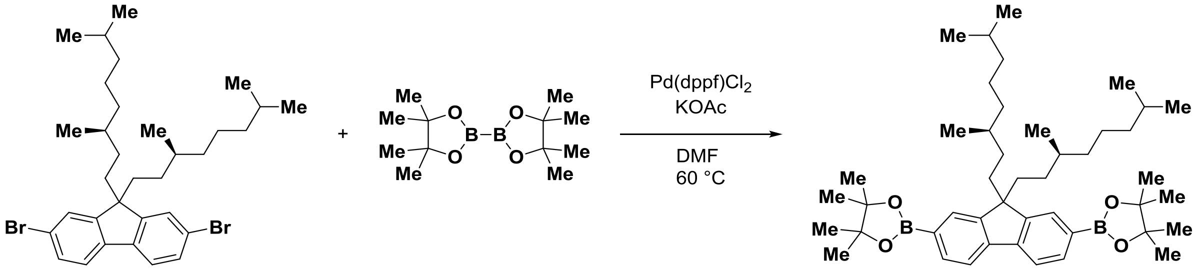

The reaction conditions are based on a report by Yoon, Wegner, and co-workers.[1] 2,7-dibromo-9,9-bis[(3*S*)-3,7-dimethyloctyl]-9*H*-fluorene (1.50 g, 2.48 mmol, 1.00 equiv.), bis(pinacolato)diboron (2.27 g, 8.93 mmol, 3.60 equiv.), Pd(dppf)Cl_2 ([1,1′-bis(diphenylphosphino)ferrocene]dichloropalladium(II), 109 mg, 149 µmol, 6.00 mol%), and potassium acetate (1.46 g, 14.9 mmol, 6.00 equiv.) were suspended in *N*,*N*-dimethylformamide (DMF, 22 ml). The mixture was submitted to three freeze/pump/thaw cycles, then heated to 60 °C under stirring. After 19 h, the reaction mixture was allowed to cool to room temperature, then diluted with diethyl ether (20 ml) and washed with 5 % aqueous LiCl solution (20 ml). The phases were separated and the aqueous layer back-extracted with diethyl ether (10 ml). The combined organic layers were washed with more 5 % aq. LiCl (20 ml), then brine (20 ml). The organic layer was dried over $MgSO_4$, filtered and the residue washed with diethyl ether until the filtrate showed no more UV absorption on a TLC plate. The filtrate was concentrated and purified by column chromatography using pentane as eluent and preparative gel permeation chromatography to afford the title compound as 472 mg (0.676 mmol, 27 % yield) of colourless foam.

NMR spectroscopy: ^{1}H NMR (400 MHz, $CDCl_3$, δ): 7.80 (dd, J = 7.5, 1.0 Hz, 2H), 7.74 (s, 2H), 7.71 (d, J = 7.6 Hz, 2H), 2.00 (pd, J = 13.1, 4.9 Hz, 4H), 1.38 (s, 24H), 1.14–0.91 (m, 13H), 0.87–0.75 (m, 15H), 0.64 (d, J = 6.5 Hz, 6H), 0.52 (tt, J = 12.5, 5.3 Hz, 2H), 0.39 (tt, J = 12.5, 5.8 Hz, 2H). ^{13}C NMR (101 MHz, $CDCl_3$, δ): 150.6, 144.2, 133.8, 129.1, 119.5, 83.8, 55.1, 39.3, 37.5, 36.7, 33.0, 30.5, 28.0, 25.1, 24.7, 22.9, 22.7, 19.7. These spectroscopic data correspond to previously reported data.[2]

poly({9,9-bis[(3*S*)-3,7-dimethyloctyl]fluorenyl-2,7-diyl}-*alt*-{benzo[2,1,3]thiadiazol-4,8-diyl}) (cPFBT)

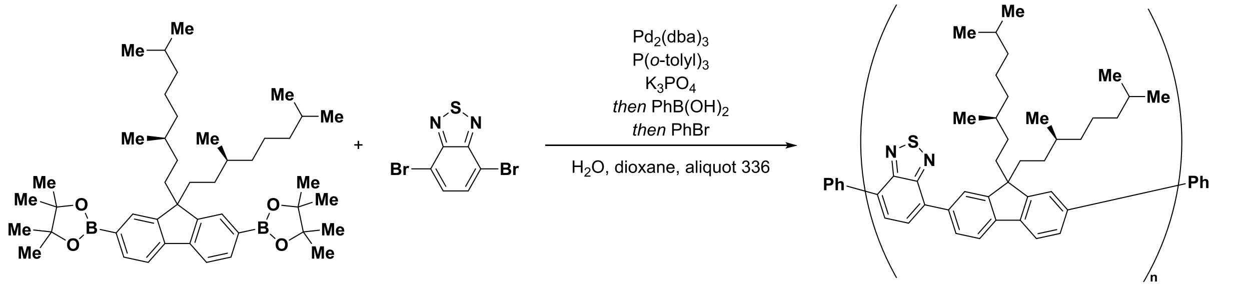

S1 (470 mg, 0.673 mmol, 1.00 equiv.), 4,7-Dibromobenzo[*c*]-1,2,5-thiadiazole (198 mg, 0.673 mmol, 1.00 equiv.), tris(dibenzylideneacetone)dipalladium(0) (12.3 mg, 13.5 µmol, 2.00 mol%), tris(*o*-tolyl)phosphine (16.4 mg, 53.8 µmol, 8.00 mol%), tribasic potassium phosphate (571 mg, 2.69 mmol, 4.00 equiv.) and one small drop of aliquot 336 were put into a 10 ml 2-neck flask. Vacuum/nitrogen were cycled three times and water (1.5 ml) and dioxane (1.5 ml) were added. The mixture was submitted to three freeze/pump/thaw cycles, then heated to 110 °C. After 19 h, a solution of phenylboronic acid (82.0 mg, 0.673 mmol, 1.00 equiv.), tris(dibenzylideneacetone)dipalladium(0) (12.3 mg, 13.5 µmol, 2.00 mol%) and tris(*o*-tolyl)phosphine (16.4 mg, 53.8 µmol, 8.00 mol%) in toluene (1 ml) was degassed by sparging with nitrogen for 10 min and then added to the reaction mixture. After an additional hour at 110 °C, a solution of bromobenzene (140 µl, 1.35 mmol, 2.00 equiv.), tris(dibenzylideneacetone)dipalladium(0) (12.3 mg, 13.5 µmol, 2.00 mol%) and tris(*o*-tolyl)phosphine (16.4 mg, 53.8 µmol, 8.00 mol%) in toluene (1 ml) was degassed by sparging with nitrogen for 10 min and then added to the reaction mixture. After one additional hour, the reaction was allowed to cool to room temperature, diluted with chloroform (15 ml) and washed with an approx. 1 wt% aqueous solution of sodium diethyldithiocarbamate trihydrate (3 x 30 ml) to remove Pd residue.[3] The organic layer was concentrated to approx. 3 ml, then added to methanol (200 ml) under fast stirring. The precipitate was filtered into a cellulose thimble and purified by successive Soxhlet extractions with methanol, acetone, and hexane (3 h each). The residue was extracted into chloroform, concentrated and purified by preparative GPC. Trailing and leading edges were cut off, then the main component was collected and re-injected. A high molecular weight fraction was obtained from the preparative GPC, precipitated in methanol (50 ml) under fast stirring, filtered and dried under high vacuum overnight to afford 24.5 mg of orange solid. GPC analysis showed M_n 15600, M_w 17200, PD 1.11.

NMR spectroscopy: ^{1}H NMR (400 MHz, $CDCl_3$, δ) 8.19–7.80 (m, integral: 9.03), 7.58 (t, *J* = 7.7 Hz, integral: 0.30), 7.53–7.46 (m, integral: 0.17), 2.17 (br. s, integral: 4.00), 1.46–1.30 (m, integral: 1.98), 1.31–0.85 (m, integral: 15.81), 0.85–0.64 (m, integral: 21.90). ^{13}C NMR (126 MHz, $CDCl_3$, δ): 154.6, 151.8, 141.2, 136.7, 133.8, 129.4, 128.8, 128.5, 128.1, 124.1, 120.2, 55.4, 39.4, 37.7, 36.9, 33.2, 30.9, 28.1, 24.9, 22.8, 22.7, 19.8.

Spectroscopic data

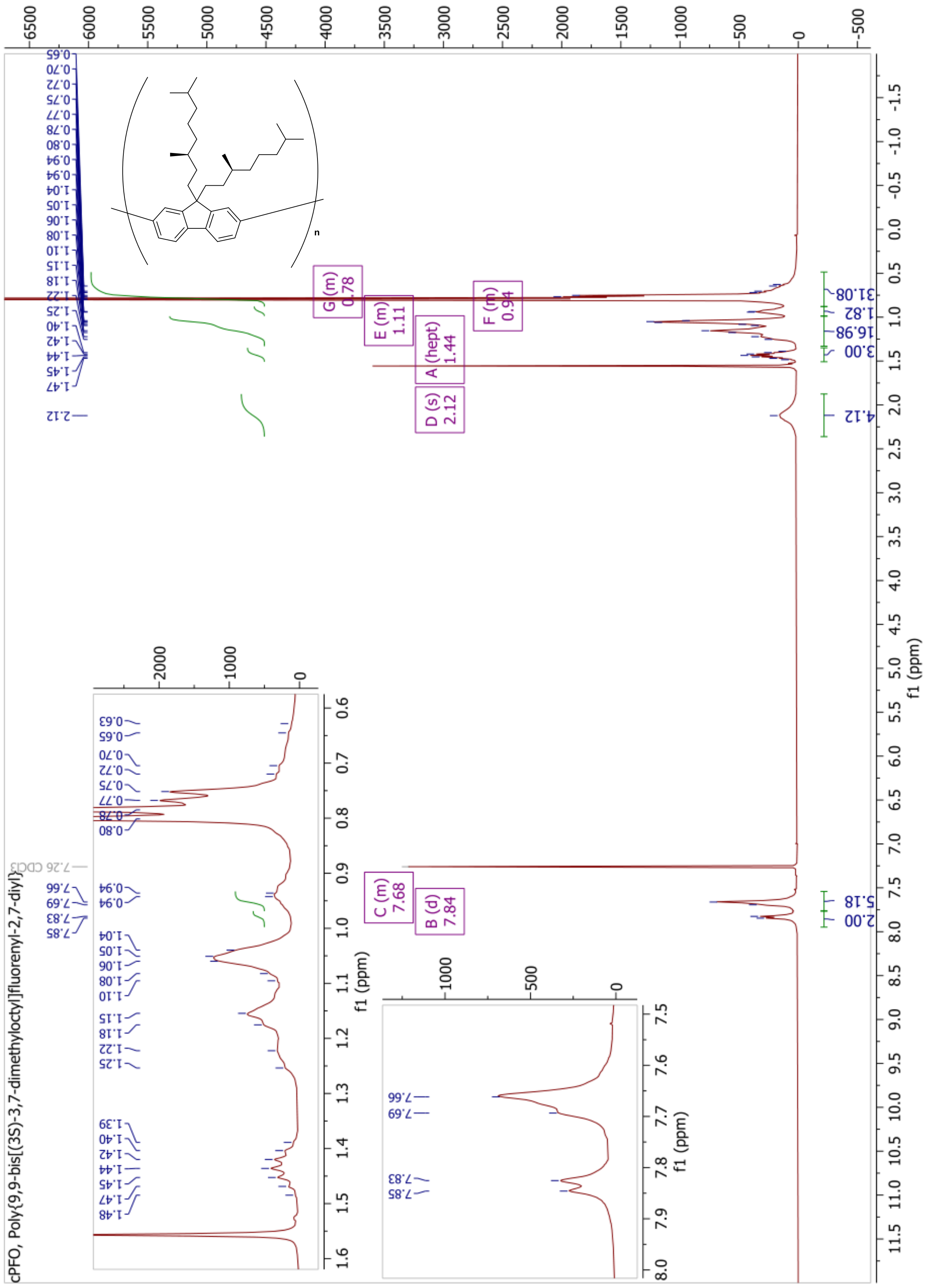

^{1}H NMR ($CDCl_3$, 20 °C) of cPFO

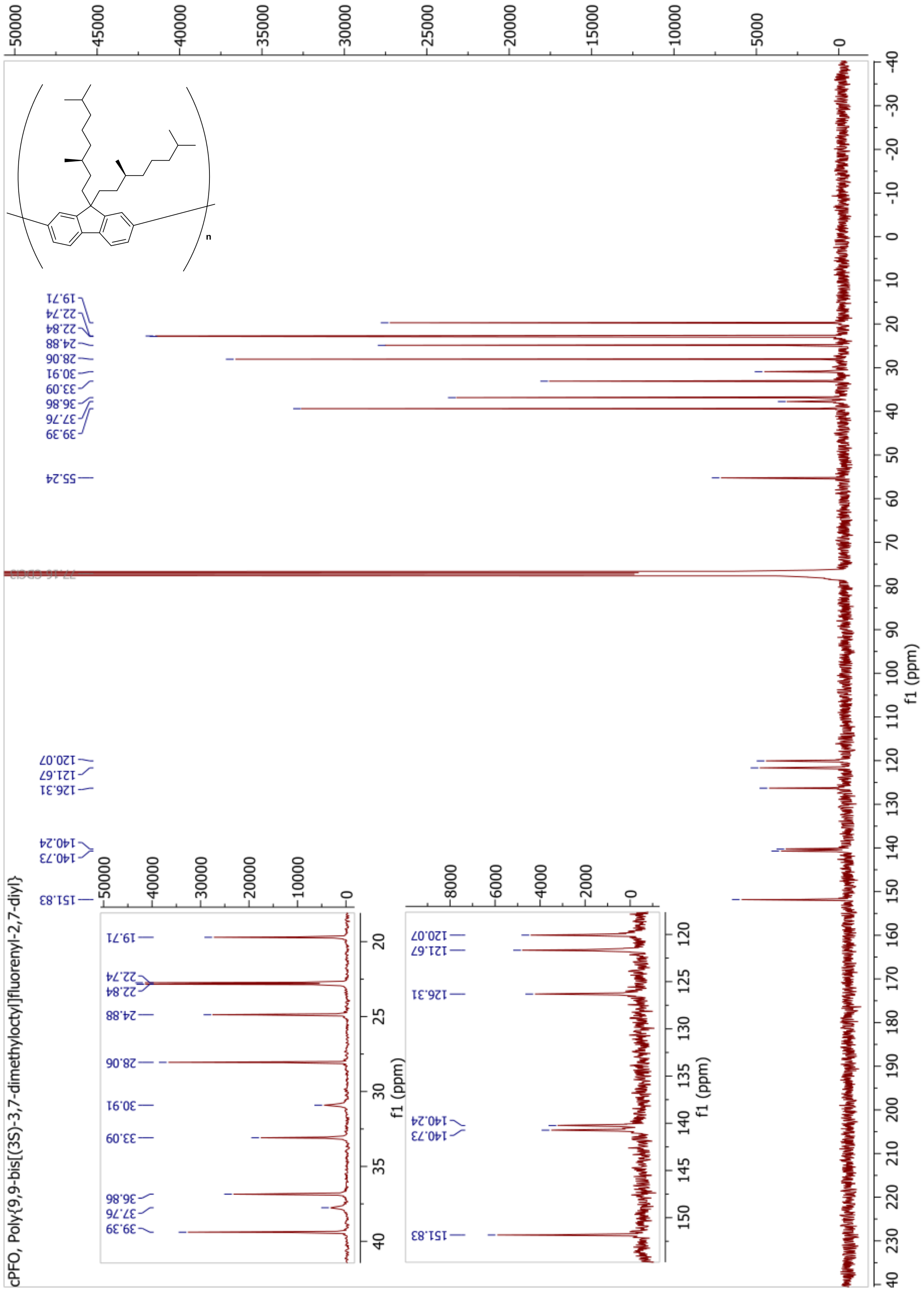

^{13}C NMR ($CDCl_3$, 80 °C) of cPFO

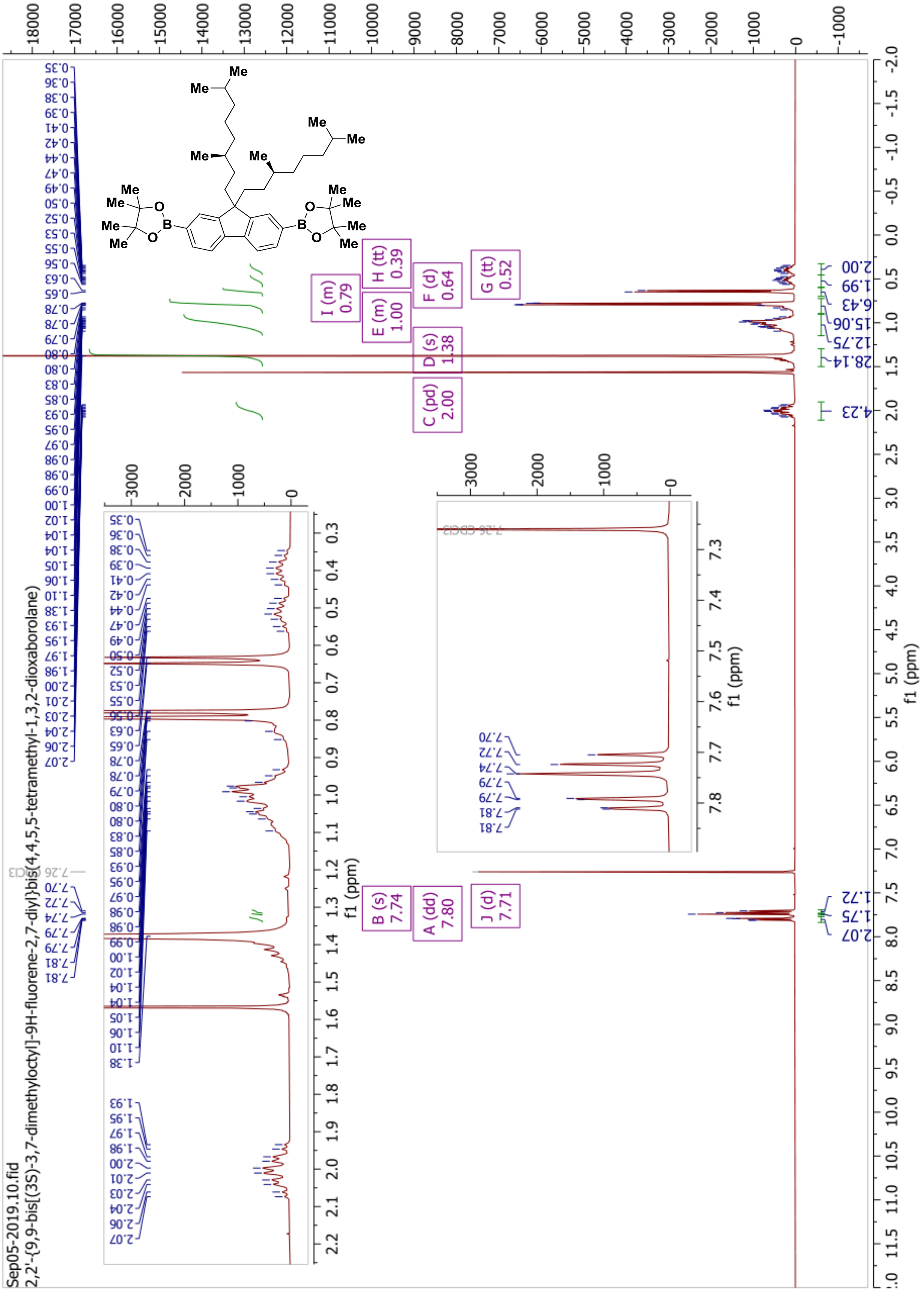

^{1}H NMR (CDCl$_3$, 23 °C) of S1

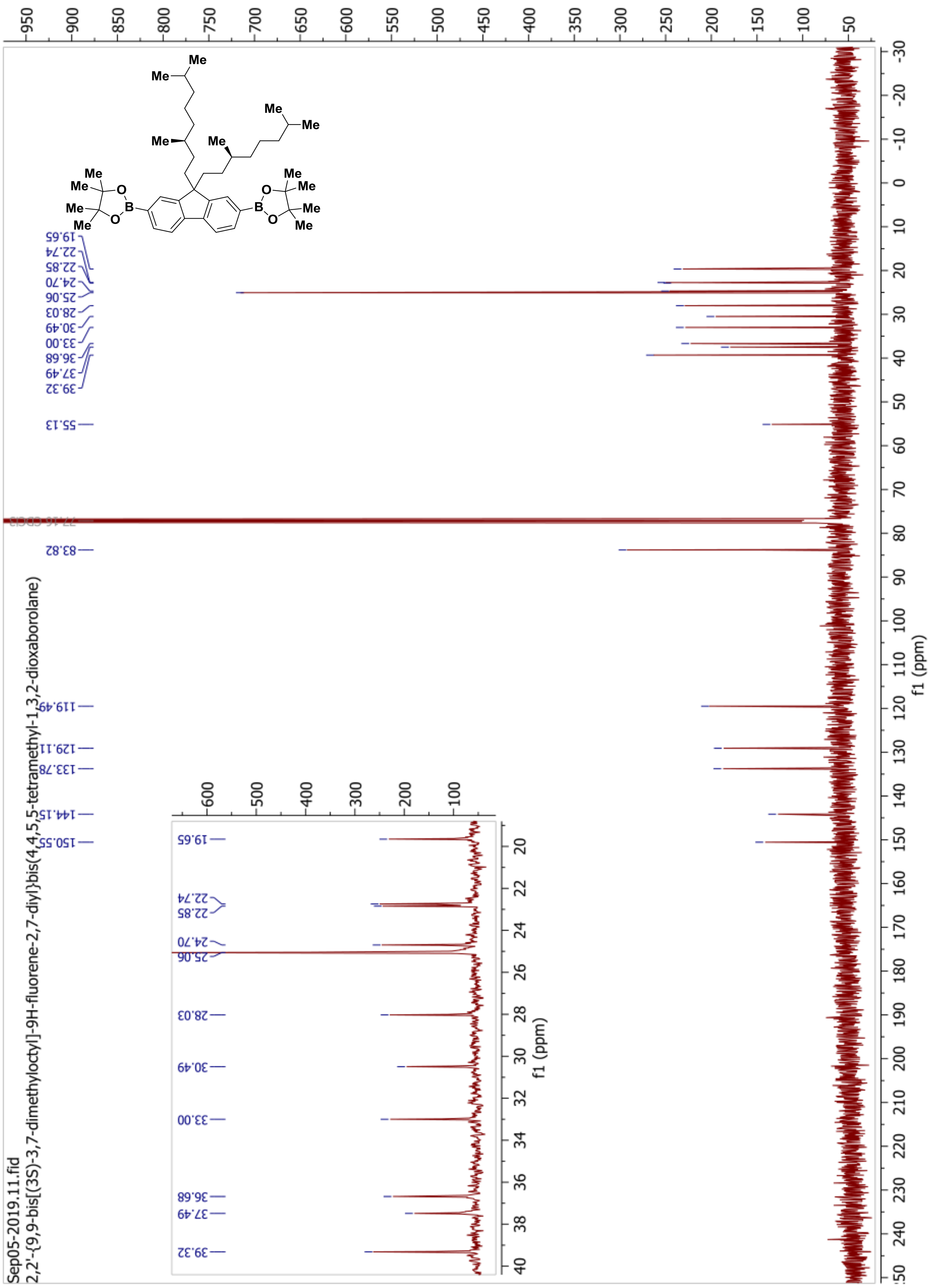

^{13}C NMR ($CDCl_3$, 23 °C) of S1

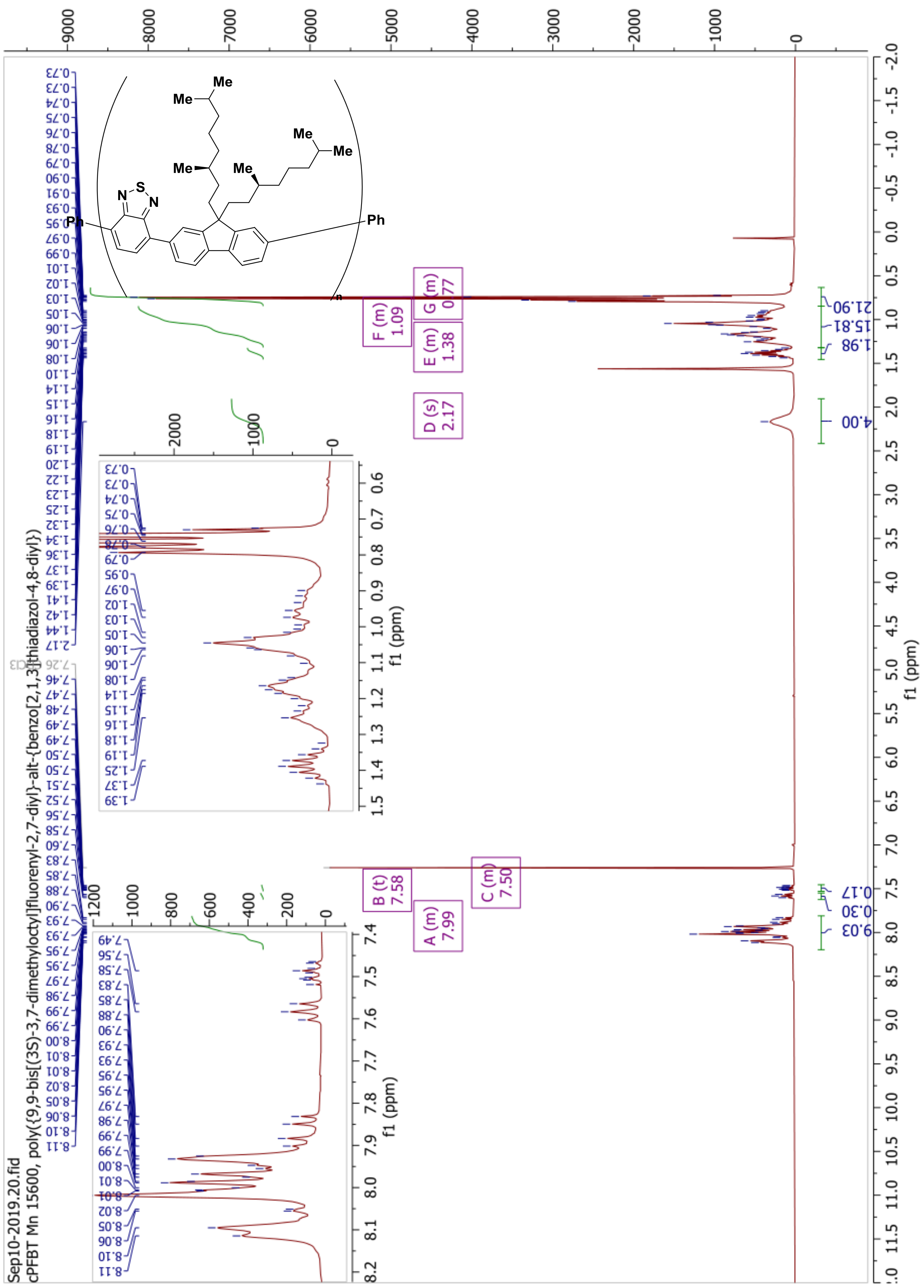

^{1}H NMR ($CDCl_3$, 23 °C) of cPFBT

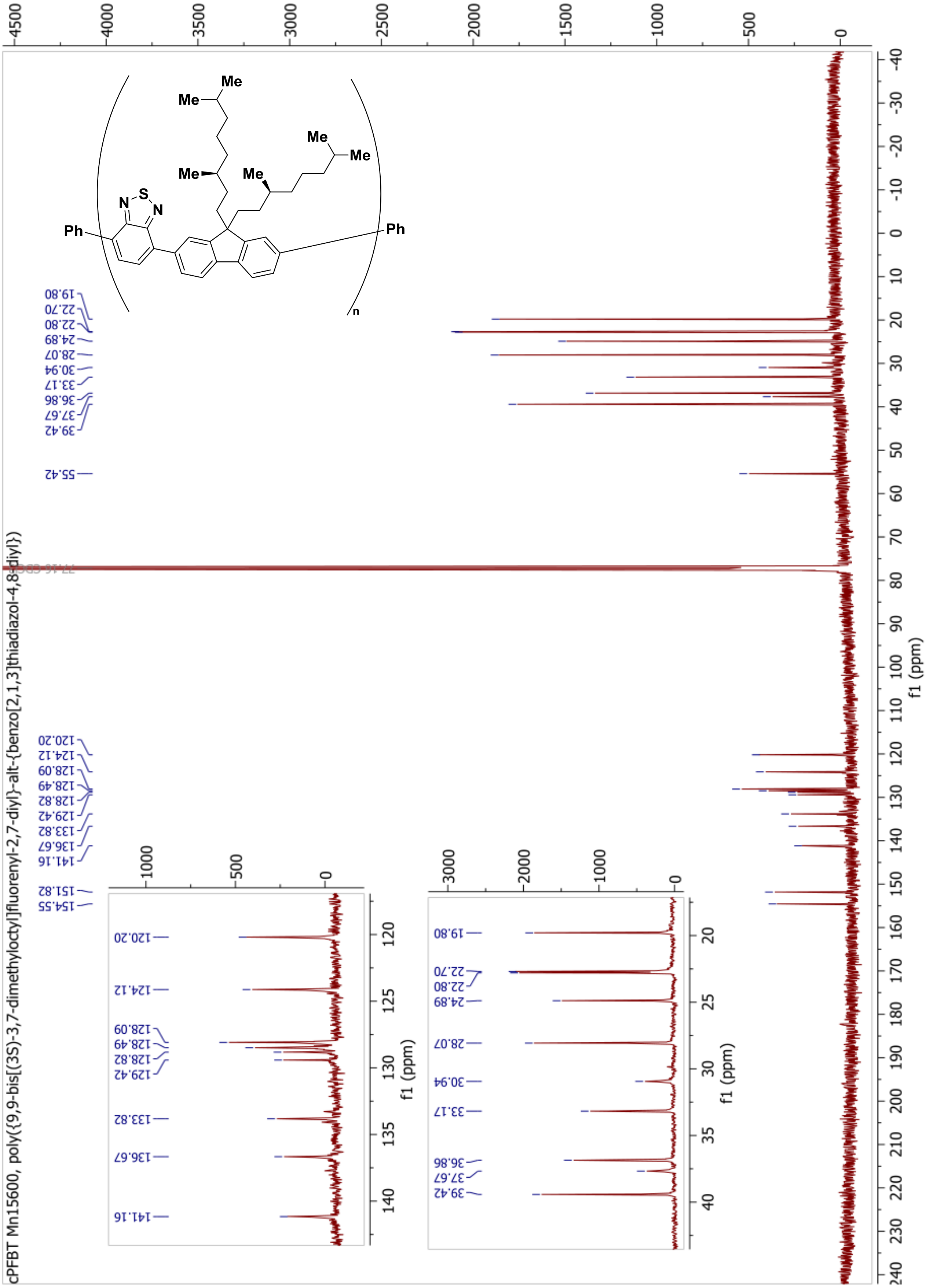

^{13}C NMR ($CDCl_3$, 23 °C) of cPFBT

Supplementary Table 1 Mechanisms used to explain observed chiroptical effects

**Material(s)**	**Dissymmetry (RT)**	**Pitch**	**Proposed model**	**Reference**
Poly(9,9′-dialkylfluorene-*alt*-2,5-dialkoxyphenyl) with various sidechains	g_{PL} 0.33 – 0.62<br>g_{abs} 0.70 – 0.82	700 – 1500 nm	Cholesteric	4
F8BT + R5011 / S5011	g_{PL} = 0.7, g_{EL} = 1.1	500 – 1000 nm	Cholesteric	5
(S,S)-ProSQ-C16 and (R,R)-ProSQ-C16	g_{abs} 0.75	N/A	Molecular level/ intrinsic	6,7
Chiral side-chain F8*-alt-DPP w/ plasticiser	g_{abs} < 0.1	Not provided	Cholesteric-like	8
Chiral side-chain PFBT (cPFBT)	g_{abs} 0.8,<br>g_{PL} 0.6,<br>g_{EL} 0.30	600 nm	Cholesteric (multi-domain)	9
Oligothiophenes	g_{abs} 10^{-5}	Not provided	Cholesteric-like	10,11
cPFBT	g_{abs} 0.8	600 nm	Cholesteric	12
Achiral BTAC molecules	g_{PL} 1.2<br>g_{abs} 0.3	N/A	3D chiral supramolecular structure	13
Chiral polyfluorene (cPFO)	g_{abs} 0.1	300 nm	Cholesteric	14
Chiral side-chain P3HT	g_{abs} 10^{-3}<br>g_{PL} 10^{-3}	N/A	Molecular level/ intrinsic	15
Chiral side-chain polyfluorene	g_{abs} 1 (thick), 0.0001 (thin)	Not provided	Long-range order, chiral LC phase	16
Functionalized nonafluorene	g_{PL} 0.6	123 nm	Cholesteric	17,18
poly[p-bis(3,7-dimethyloctyl)phenyleneethynylene]	g_{abs} 0.38<br>g_{PL} 0.19	25 nm	Helical twisted bundle	19
F8BT + aza[6]H	g_{PL} 0.5	N/A	Chiroptical co-crystalline phase	20
F8BT + aza[6]H	g_{abs} 1.1,<br>g_{PL} 0.4,<br>g_{EL} 1.05	N/A	Localised CP-emission due to molecular chirality	21
F8T2 + aza[6]H	g_{abs} 1.6	N/A	Magneto-electric coupling	This work
PFO + aza[6]H	g_{abs} 0.3			
cPFO	g_{abs} 0.5			
cPFBT	g_{abs} 0.8			

Supplementary Table 2 Extracted parameters discussed in this work

	Unit	PFO	F8T2	F8BT	cPFO	cPFBT
T_g	°C	60 - 70	70 - 80	100 - 110		
$\pi \rightarrow \pi^*$	nm	385	455	479	395	470
λ_{CDMax}	nm	405	485	485	405	479
T_{CDMax}	°C	160	120	125	140	140
$\vert CD\ Max \vert$	mdeg	3,110	21,500	10,200	13,900	12,100

Supplementary Figure 1: Extracted dissymmetry for ACPCA and CSCP thin films

Extracted dissymmetry (g-factor) for ACPCA (a) and CSCP (b) thin films. These data are extracted from the spectra in Figure 2.

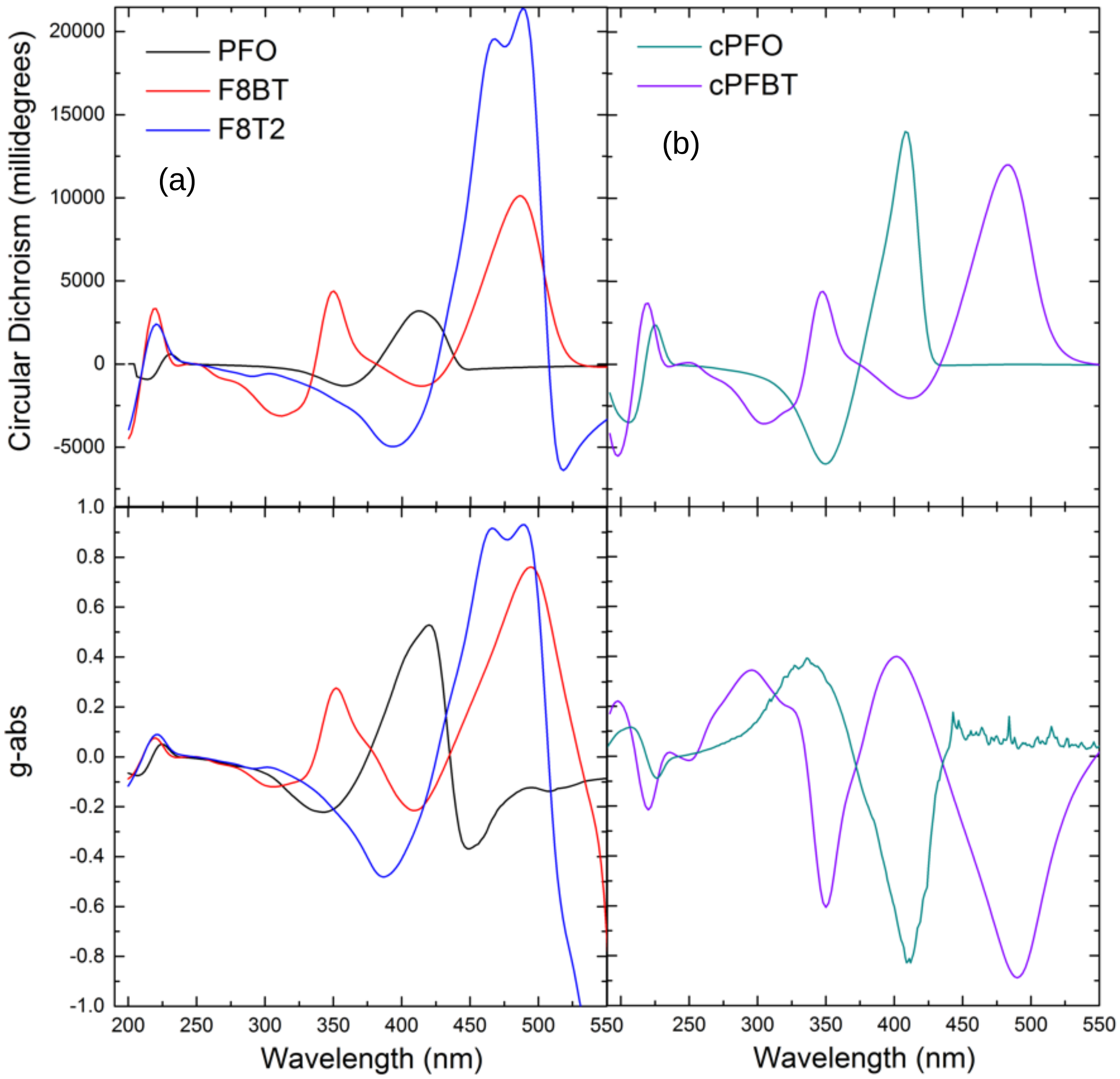

Supplementary Figure 2: Spatially resolved circular dichroism of annealed polymer thin films

Investigation of the uniformity of the chiroptical response of annealed achiral polymer – chiral additive thin films (F8BT : aza[P]) performed by mapping a 40 × 40 grid array area of 0.5 mm step size with a beam diameter of 0.05 – 0.100 mm. The chiroptical response was recorded at λ = 481 nm. Homogeneity of the chiral response across these length scales sample permits further characterisation using MMSE, as it confirms that the sample is uniform across the within beam size of the ellipsometer (3 – 4 mm).

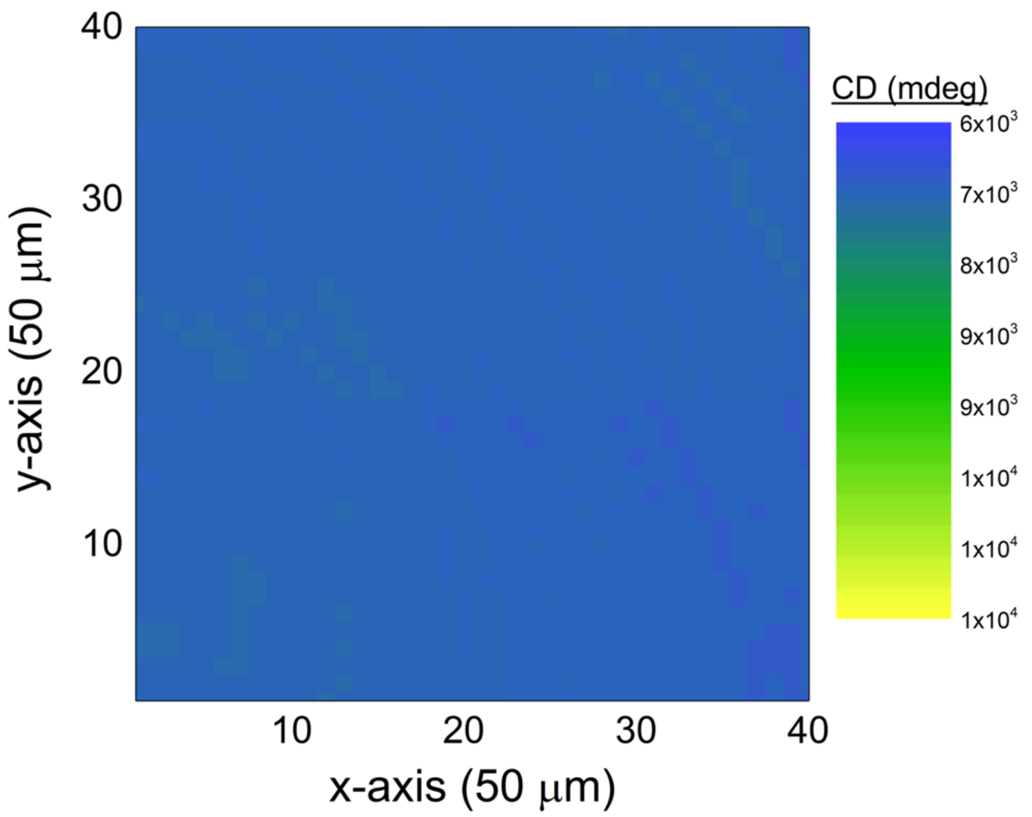

Supplementary Discussion 1 Mueller Matrix Spectroscopic Ellipsometry

The MM is written as a 4x4 matrix to describe transformation from incoming to outgoing Stokes parameters:

$$\begin{pmatrix} S_0' \\ S_1' \\ S_2' \\ S_3' \end{pmatrix}_{out} = \begin{bmatrix} M_{11} & M_{12} & M_{13} & M_{14} \\ M_{21} & M_{22} & M_{23} & M_{24} \\ M_{31} & M_{32} & M_{33} & M_{34} \\ M_{41} & M_{42} & M_{43} & M_{44} \end{bmatrix} \begin{pmatrix} S_0 \\ S_1 \\ S_2 \\ S_3 \end{pmatrix}_{in}$$

Supplementary Equation 1

The normalized MM for an isotropic sample or one without cross-polarization can be written in terms of N=cos(2Ψ), C=sin(2Ψ)cos(Δ), and S=sin(2Ψ)sin(Δ) as,

$$\begin{pmatrix} S_0' \\ S_1' \\ S_2' \\ S_3' \end{pmatrix}_{out} = \begin{bmatrix} 1 & -N & 0 & 0 \\ -N & 1 & 0 & 0 \\ 0 & 0 & C & S \\ 0 & 0 & -S & C \end{bmatrix} \begin{pmatrix} S_0 \\ S_1 \\ S_2 \\ S_3 \end{pmatrix}_{in}$$

Supplementary Equation 2

where Ψ and Δ are the isotropic ellipsometry parameters.

For non-depolarizing materials, the differential Mueller Matrix can be written as follows:

$$\text{Differential MM} = \begin{bmatrix} \text{A} & \text{-LD} & \text{-LD'} & \text{CD} \\ \text{-LD} & \text{A} & \text{CB} & \text{LB'} \\ \text{-LD'} & \text{-CB} & \text{A} & \text{-LB} \\ \text{CD} & \text{-LB'} & \text{LB} & \text{A} \end{bmatrix}$$

Supplementary Equation 3

where CD: Circular dichroism, CB: Circular birefringence, LD: Horizontal LD, LB: Horizontal LB, LD': 45° LD, LB': 45° LB, A: Absorbance

As can be seen above, CD and circular birefringence (CB) appear in the m_{14}/m_{41} and m_{23}/m_{32} elements of the differential Mueller matrix, respectively. The differential Mueller matrix (dMM) is calculated here as the matrix logarithm of the Mueller matrix [dMM=Log(MM)] which, in first approximation, can also be written as dMM≈I+MM, I being the 4x4 identity matrix. Therefore, Mueller matrix elements M_{14}/M_{41} and M_{23}/M_{32} are the key elements for the quantification of CD and CB, respectively.

Dielectric permittivity tensor for materials with uniaxial anisotropy:

$$D = \begin{bmatrix} \varepsilon_0 & 0 & 0 \\ 0 & \varepsilon_0 & 0 \\ 0 & 0 & \varepsilon_0 \end{bmatrix}$$

Supplementary Equation 4

Where ε refers to the permittivity. In uniaxial media, two of the diagonal terms are the same, and one is different. In this case the z-axis is the extraordinary axis (whilst the x- and y-axes are ordinary).

Bragg reflection occurs when:

$$\lambda = \left(\frac{n_e + n_0}{2}\right) p$$

Supplementary Equation 5

where p is the pitch – the cholesteric-stack thickness required for molecules to make a complete turn (360°)

To describe optical activity requires the complete 6×6 constitutive tensor that describes the complete electromagnetic interaction with the material and has the magneto-electric coupling terms in the off-diagonal blocks.

$$\begin{bmatrix} D \\ B \end{bmatrix} = \begin{bmatrix} \varepsilon & \zeta \\ \xi & \mu \end{bmatrix} \begin{bmatrix} E \\ H \end{bmatrix}$$

Supplementary Equation 6

For materials obeying Lorentz reciprocity, the following relations are implied:

$$\boldsymbol{\varepsilon} = \boldsymbol{\varepsilon}^T \text{ , } \boldsymbol{\mu} = \boldsymbol{\mu}^T, \boldsymbol{\zeta} = -\boldsymbol{\xi}^T$$

The magneto-electric tensors can in general be written as $\boldsymbol{\zeta} = \boldsymbol{\chi} - \boldsymbol{i\alpha}$ and , $\boldsymbol{\xi} = \boldsymbol{\chi}^{\mathrm{T}} - \boldsymbol{i\alpha^T}$, where $\boldsymbol{\chi}$ is the non-reciprocity or Tellegen tensor and $\boldsymbol{\alpha}$ is the chirality tensor. To satisfy the Lorentz reciprocity requirement above, it follows that $\boldsymbol{\chi} = \mathbf{0}$ and $\boldsymbol{\alpha} \neq \mathbf{0}$ must be fulfilled, where the latter relation is meant to imply that at least one of the tensor elements is not equal to zero.

Both ε and α are complex numbers and are described as (Supplementary Equation 7):

$$\varepsilon = \varepsilon_1 - i\varepsilon_2$$
$$\alpha = \alpha_1 - i\alpha_2$$

Supplementary Figure 3: Mueller Matrix Spectroscopic Ellipsometry (MMSE) data recorded in reflection and transmission for neat, annealed ACP films.

PFO. Note, no cross-polarization is witnessed in the off diagonal 4X4 blocks (upper right and lower left) of either the reflection or transmission MMSE. The model curves are matched to both reflected and transmitted MMSE data (along with normal incidence intensity transmission) by describing the uniaxial anisotropy of the PFO with the determined film thickness of 95.4 nm.

Reflection MMSE:

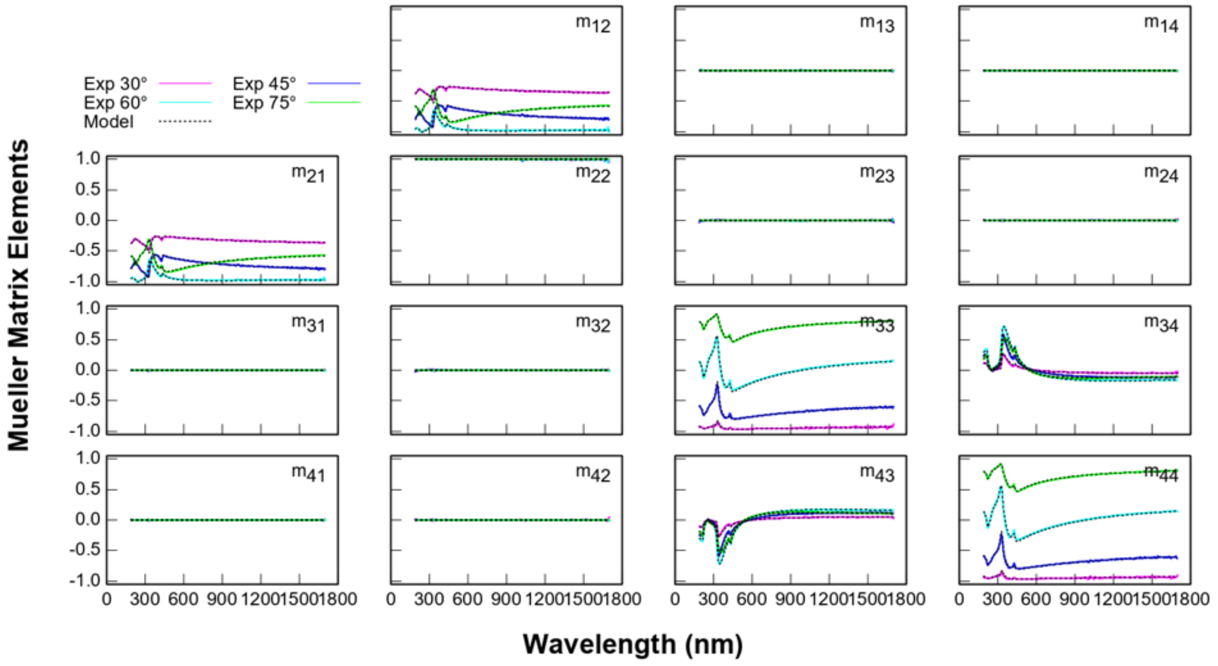

Transmission MMSE:

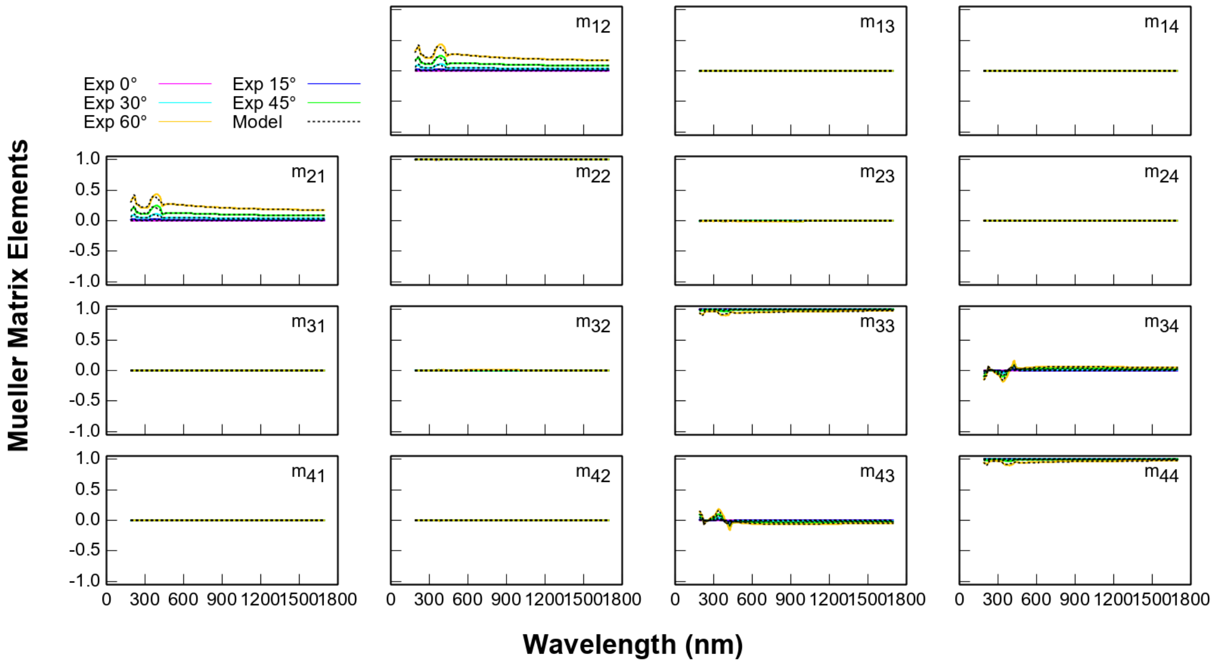

F8T2. Note, no cross-polarization is witnessed in the off diagonal 4x4 blocks (upper right and lower left) of either the reflection or transmission MMSE. The model curves are matched to both reflected and transmitted MMSE data (along with normal incidence intensity transmission) by describing the uniaxial anisotropy of the F8T2 with the determined film thickness of 92.1 nm.

Reflection MMSE:

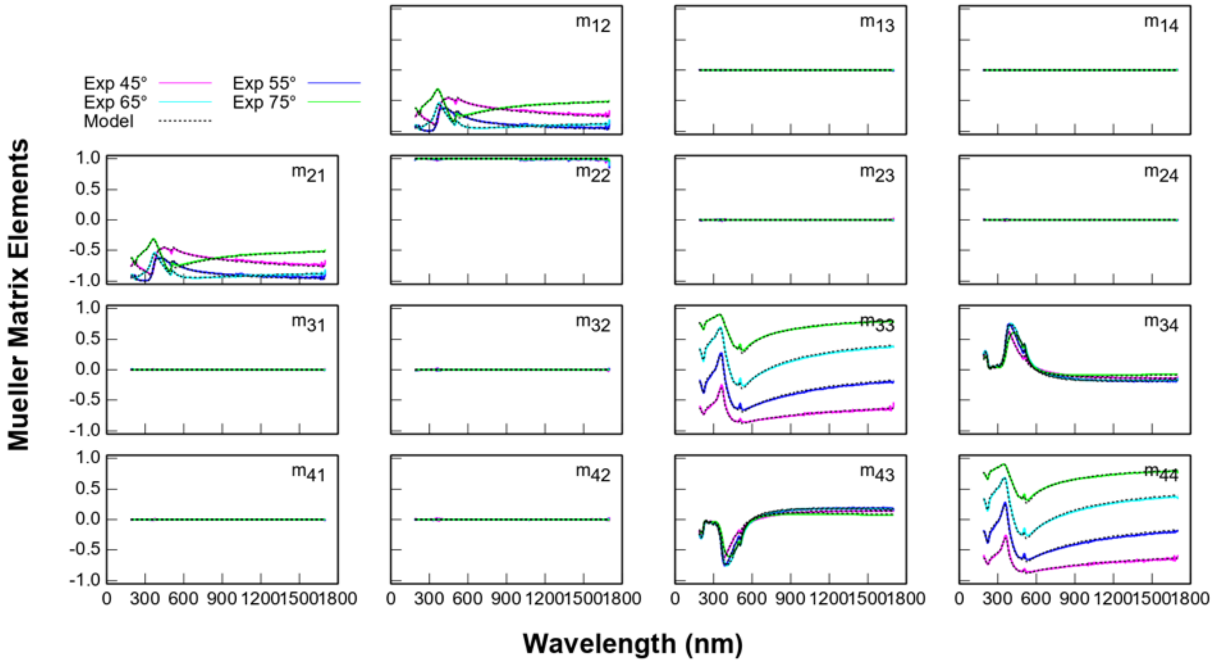

Transmission MMSE:

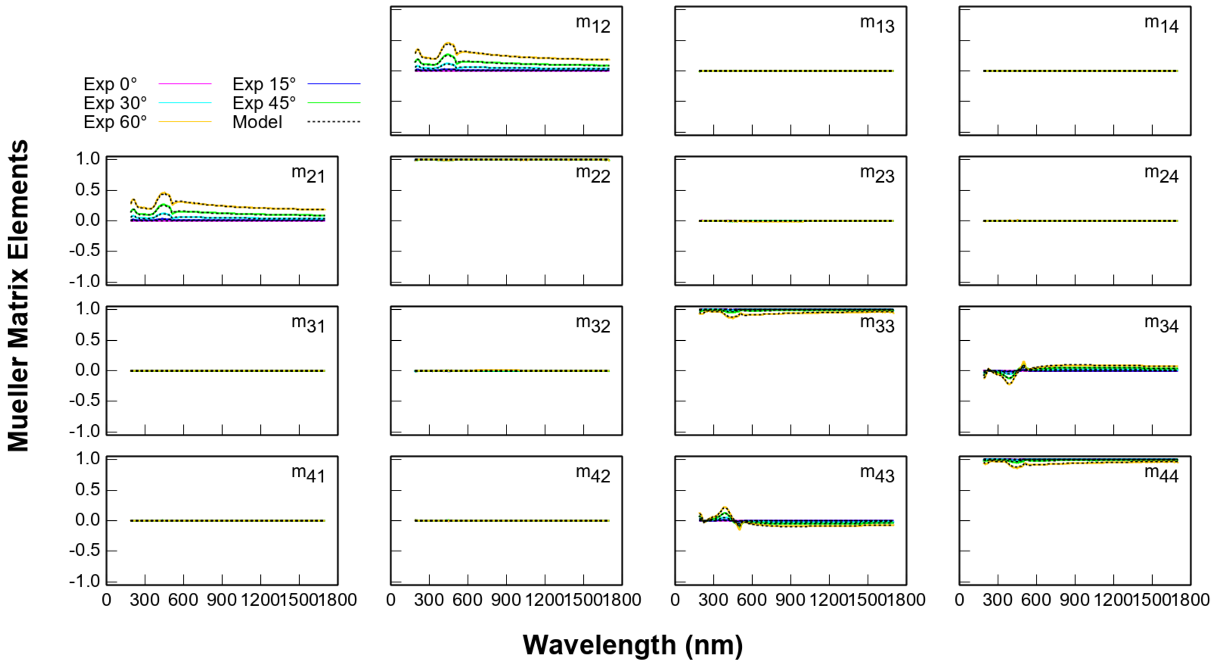

F8BT. Note, no cross-polarization is witnessed in the off diagonal 4 x 4 blocks (upper right and lower left) of either the reflection or transmission MMSE. The model curves are matched to both reflected and transmitted MMSE data (along with normal incidence intensity transmission) by describing the uniaxial anisotropy of the F8BT with the determined film thickness of 132.5 nm.

Reflection MMSE:

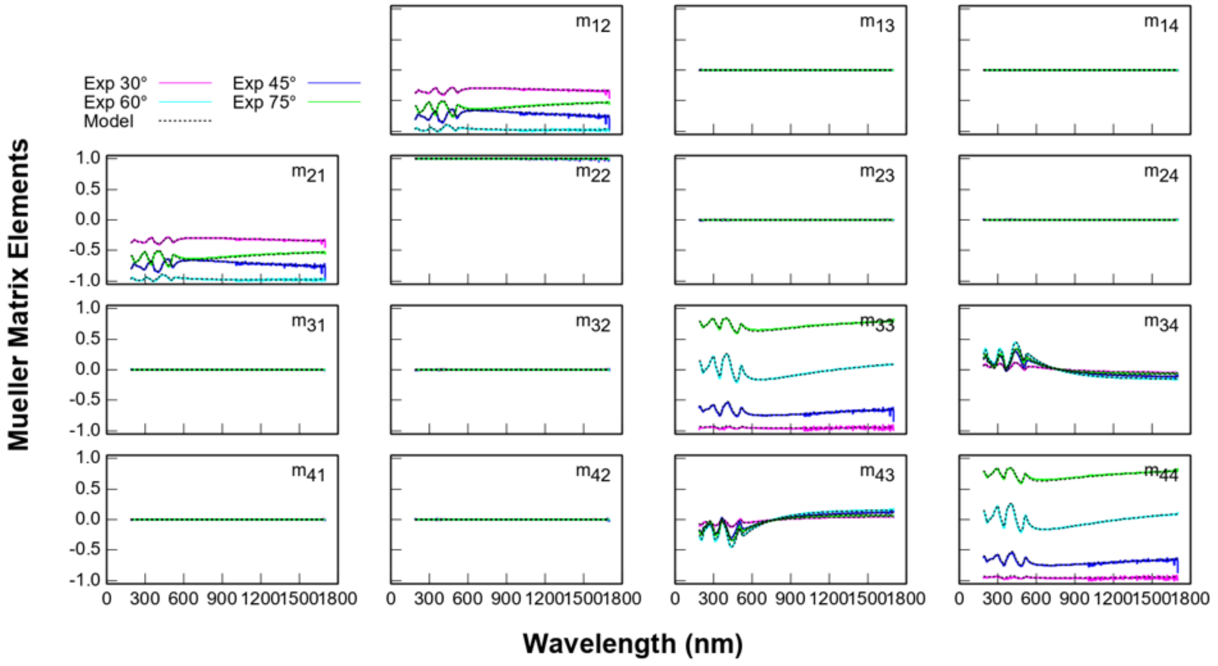

Transmission MMSE:

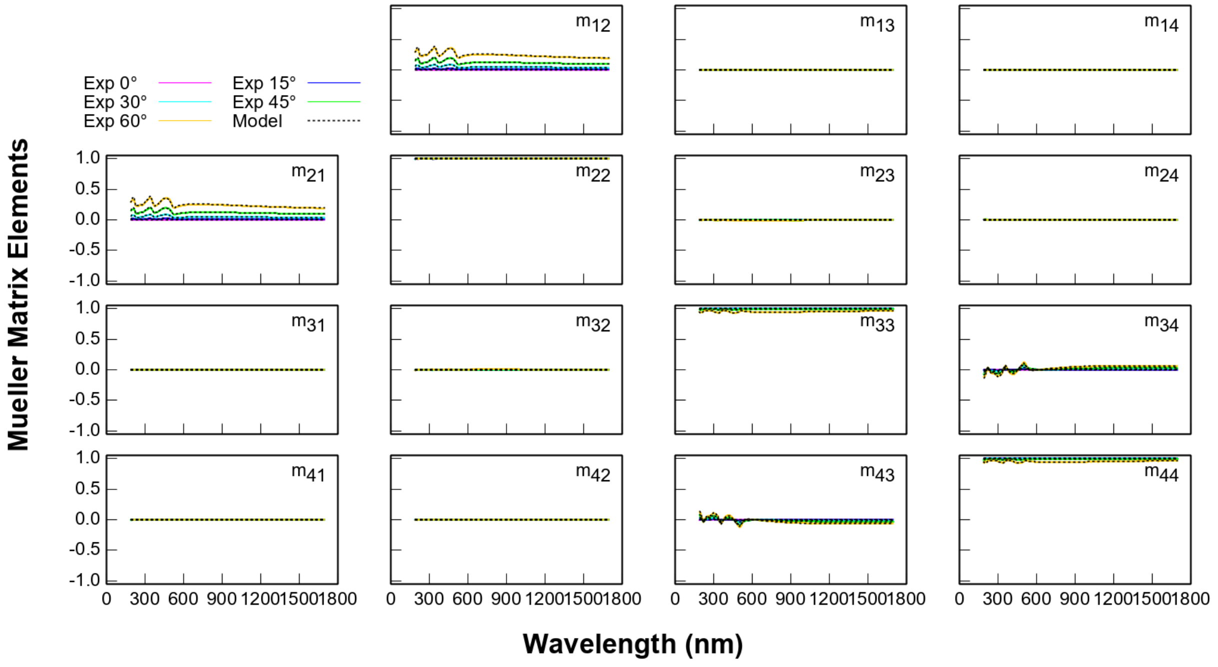

Supplementary Figure 4: Anisotropic dielectric function for neat, annealed ACP films.

PFO:

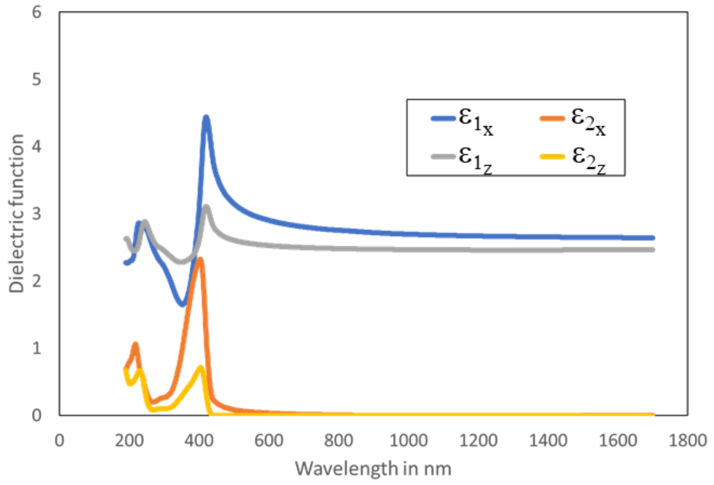

F8BT:

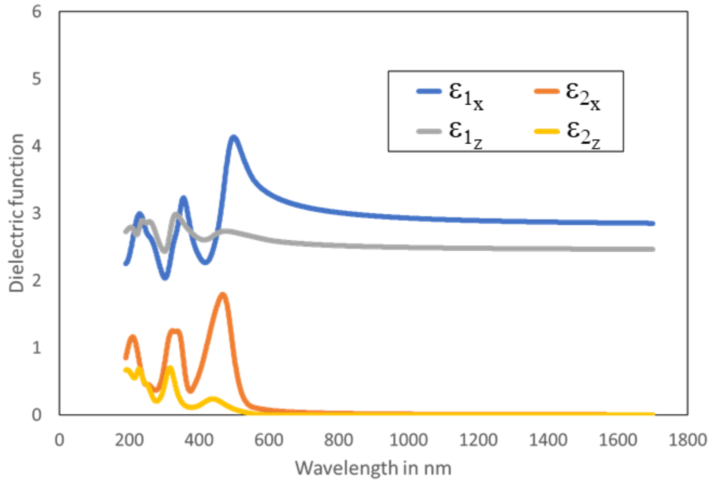

F8T2:

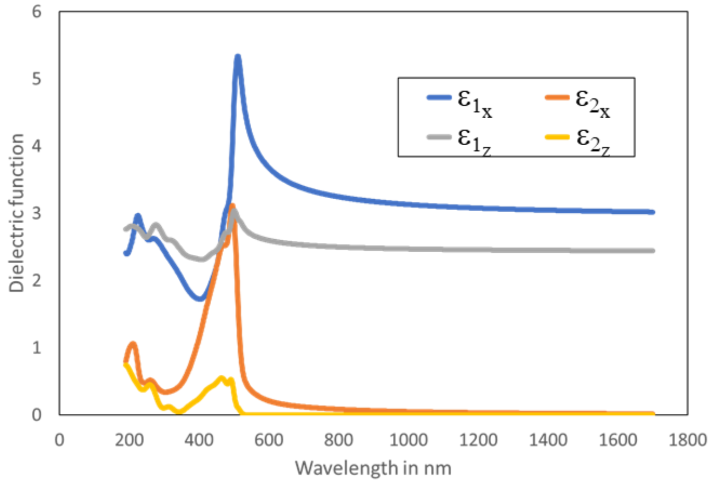

Supplementary Figure 5: MMSE data recorded in reflection and transmission for ACPCA thin films annealed at $T_{CD\ Max}$.

PFO: aza[*M*]H While there is no cross-polarization in the reflection MMSE, the circular polarization properties are witnessed in the Transmission MMSE curves (M_{41}, M_{32}, M_{23}, and M_{14}). The model curves are matched to both reflected and transmitted MMSE data (along with normal incidence intensity transmission) by describing both the uniaxial dielectric function and uniaxial optical activity of the PFO:aza[M]H with the determined film thickness of 56.6 nm

Reflection MMSE:

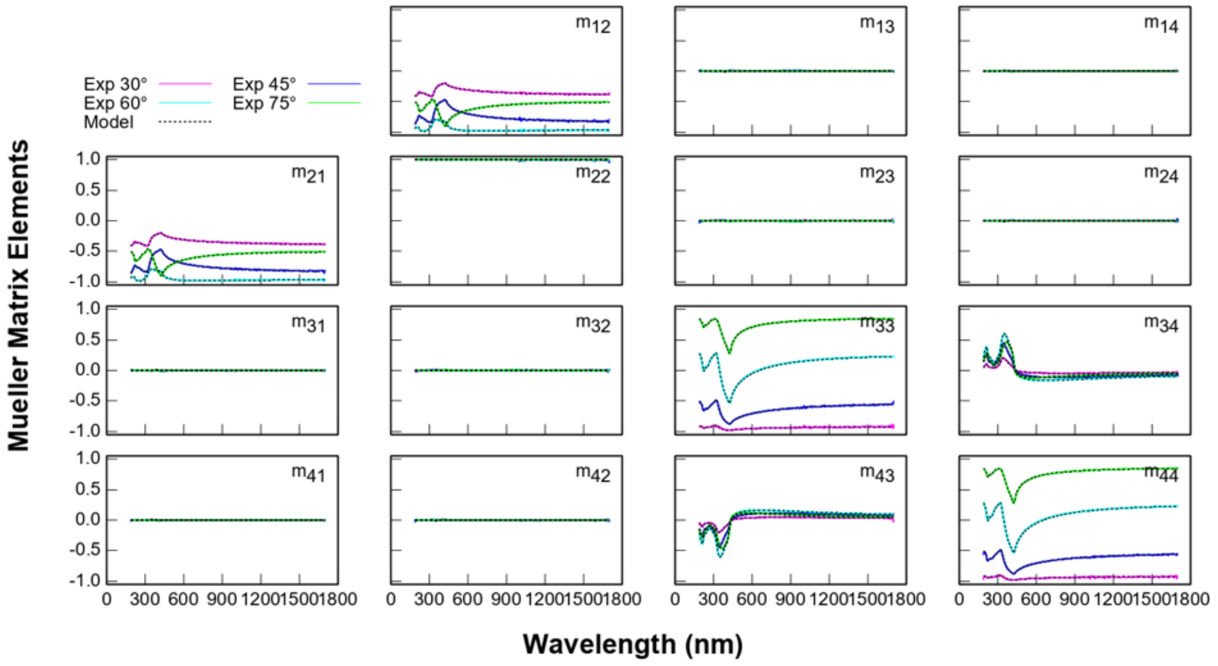

Transmission MMSE:

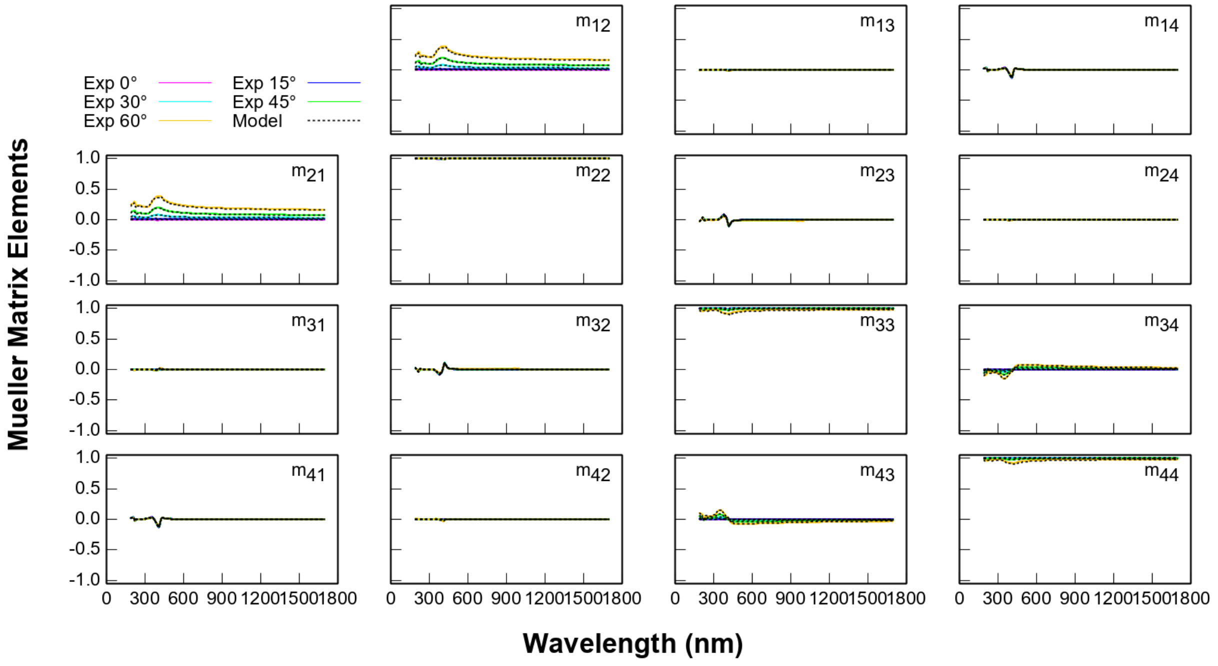

F8T2: aza[*M*]H While there is no cross-polarization in the reflection MMSE, the circular polarization properties are witnessed in the Transmission MM curves (M_{41}, M_{32}, M_{23}, and M_{14}). The model curves are matched to both reflected and transmitted MMSE data (along with normal incidence intensity transmission) by describing both the uniaxial dielectric function and uniaxial optical activity of the F8T2:aza[M]H with the determined film thickness of 129.7 nm

Reflection MMSE:

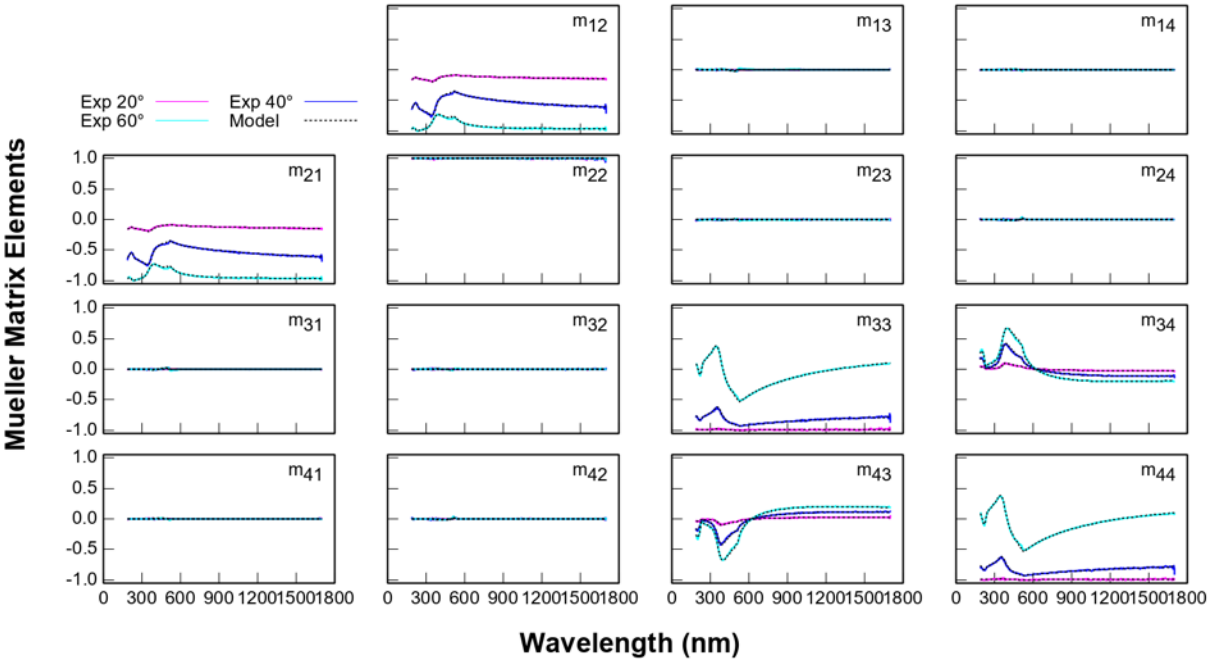

Transmission MMSE:

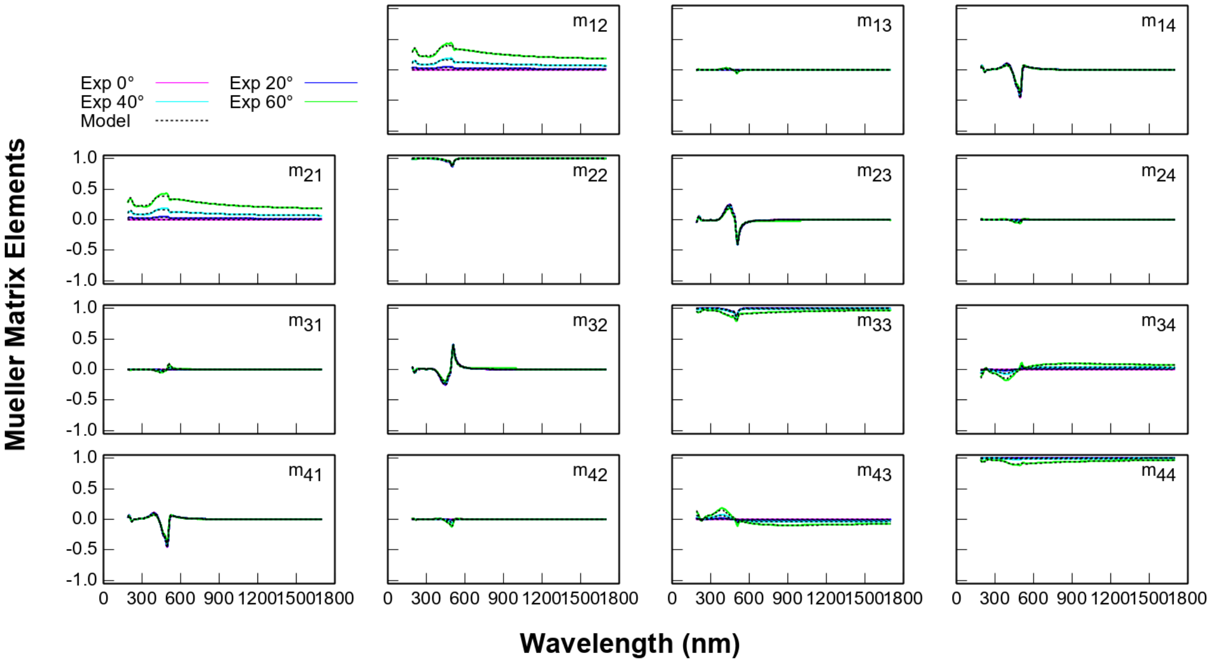

F8BT: aza[*M*]H While there is no cross-polarization in the reflection MMSE, the circular polarization properties are witnessed in the Transmission MM curves (M_{41}, M_{32}, M_{23}, and M_{14}). The model curves are matched to both reflected and transmitted MMSE data (along with normal incidence intensity transmission) by describing both the uniaxial dielectric function and uniaxial optical activity of the F8BT:aza[M]H with the determined film thickness of 133.1 nm

Reflection MMSE:

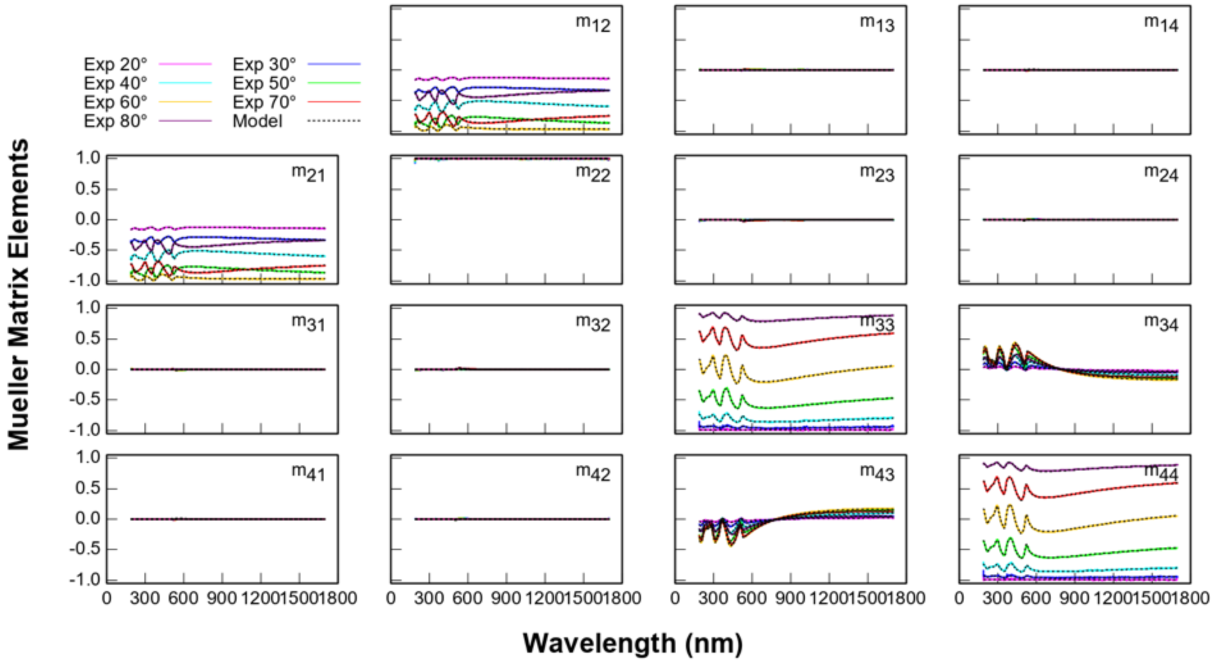

Transmission MMSE:

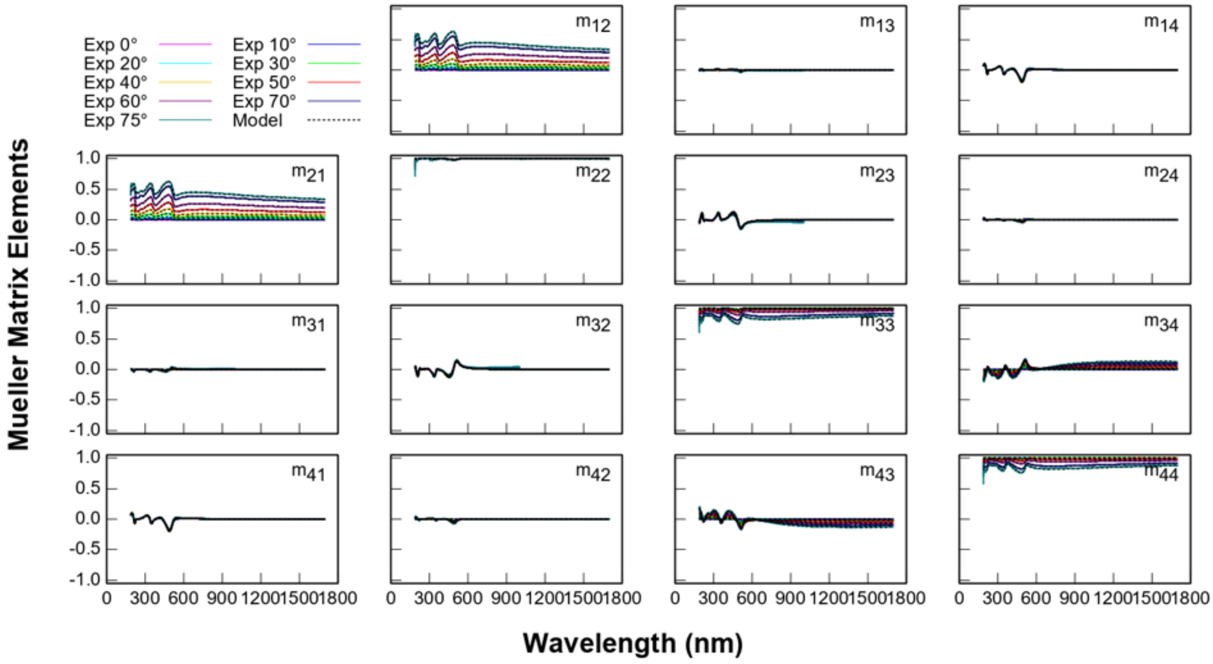

Supplementary Figure 6 MMSE data recorded in transmission and reflection for annealed, unaligned CSCP and ACPCA thin films.

Both data sets exhibit CD and CB effects along the antidiagonal MM elements in transmission but not in reflection.

PFO:aza[*M*] Transmission MMSE for ~ 56 nm thick film:

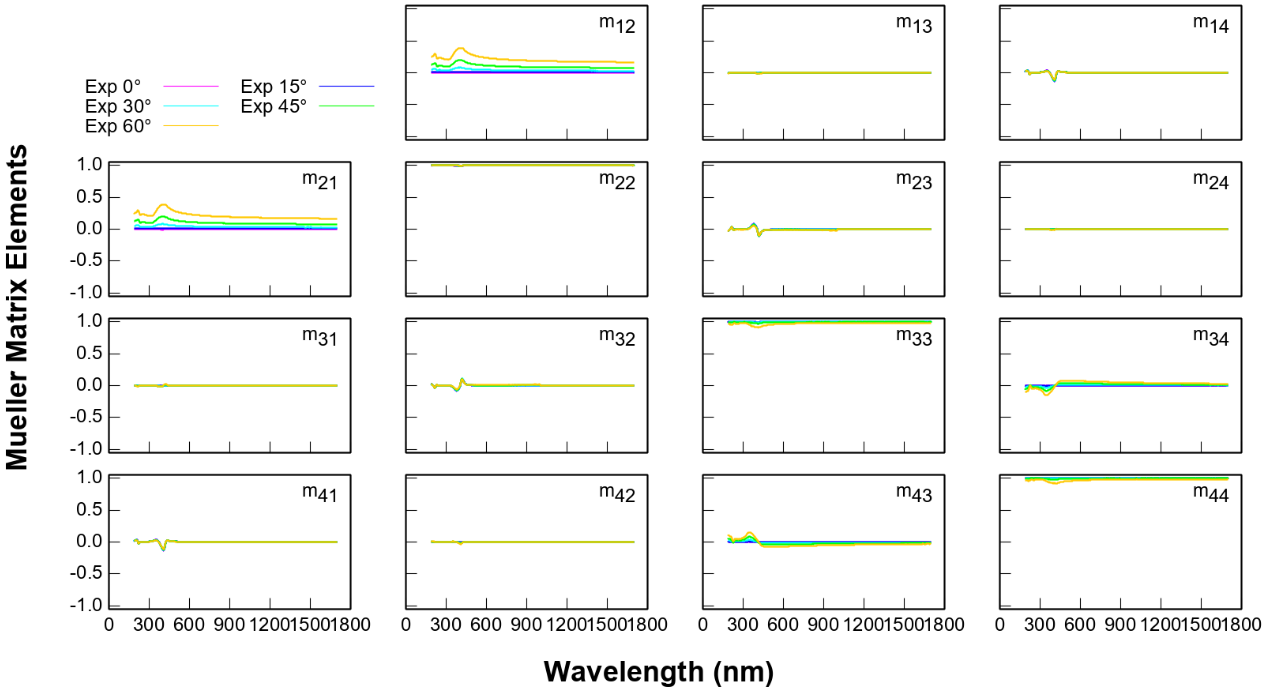

cPFO Transmission MMSE for ~ 52 nm thick film:

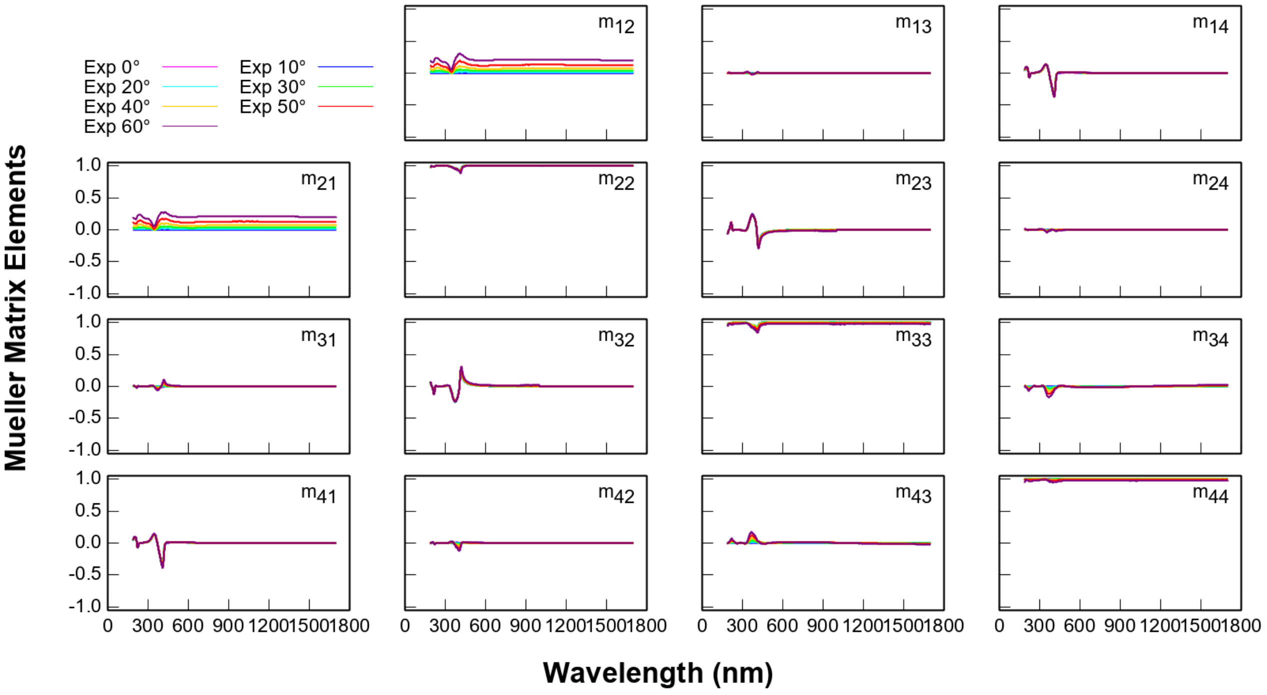

PFO:aza[*M*] Reflection MMSE for ~ 56 nm thick film:

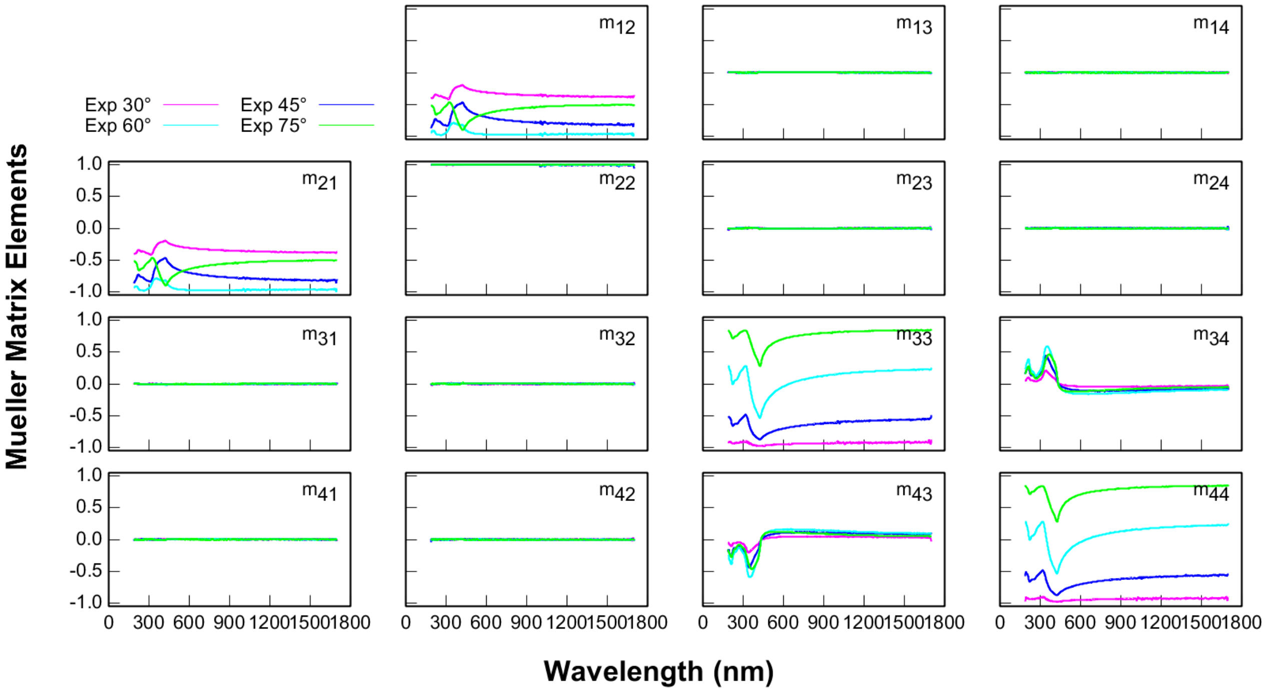

cPFO Reflection MMSE for ~ 52 nm thick film:

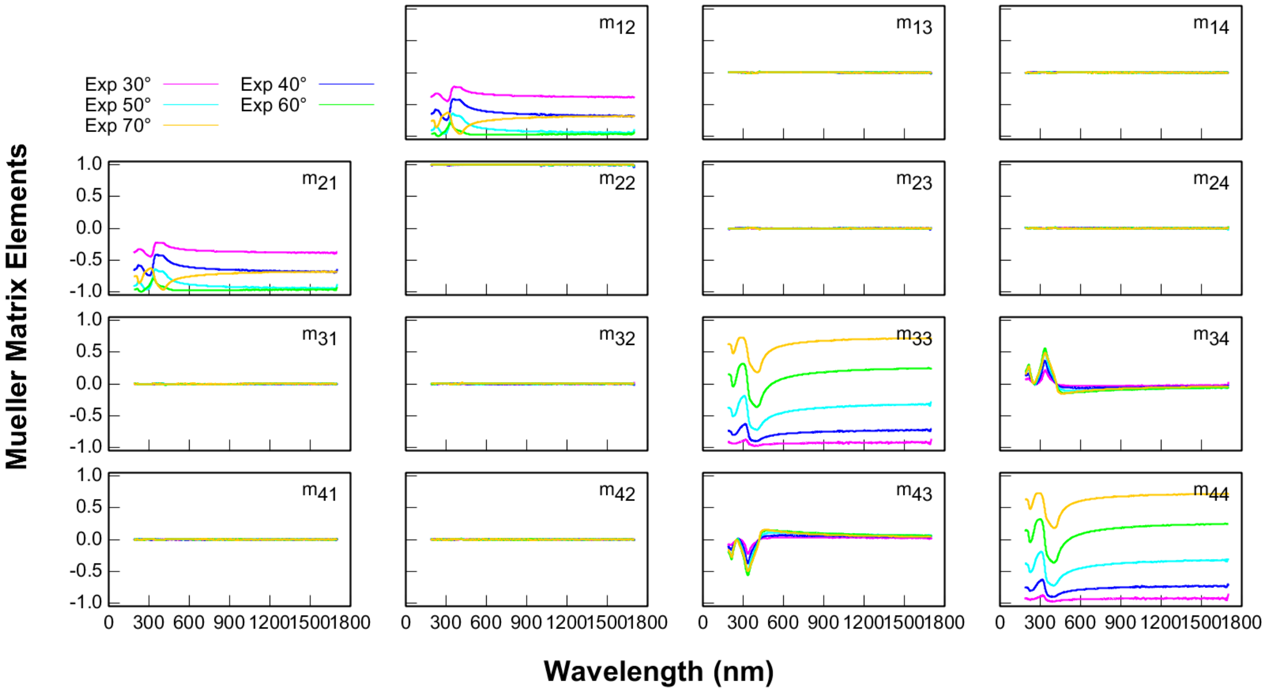

MM_{14} and MM_{23} recorded in reflection and transmission for annealed, unaligned CSPCs shows the strong CD and CB effects are confined to the transmission data and do not occur in the reflected MMSE data.

cPFO film of ~ 52 nm thickness

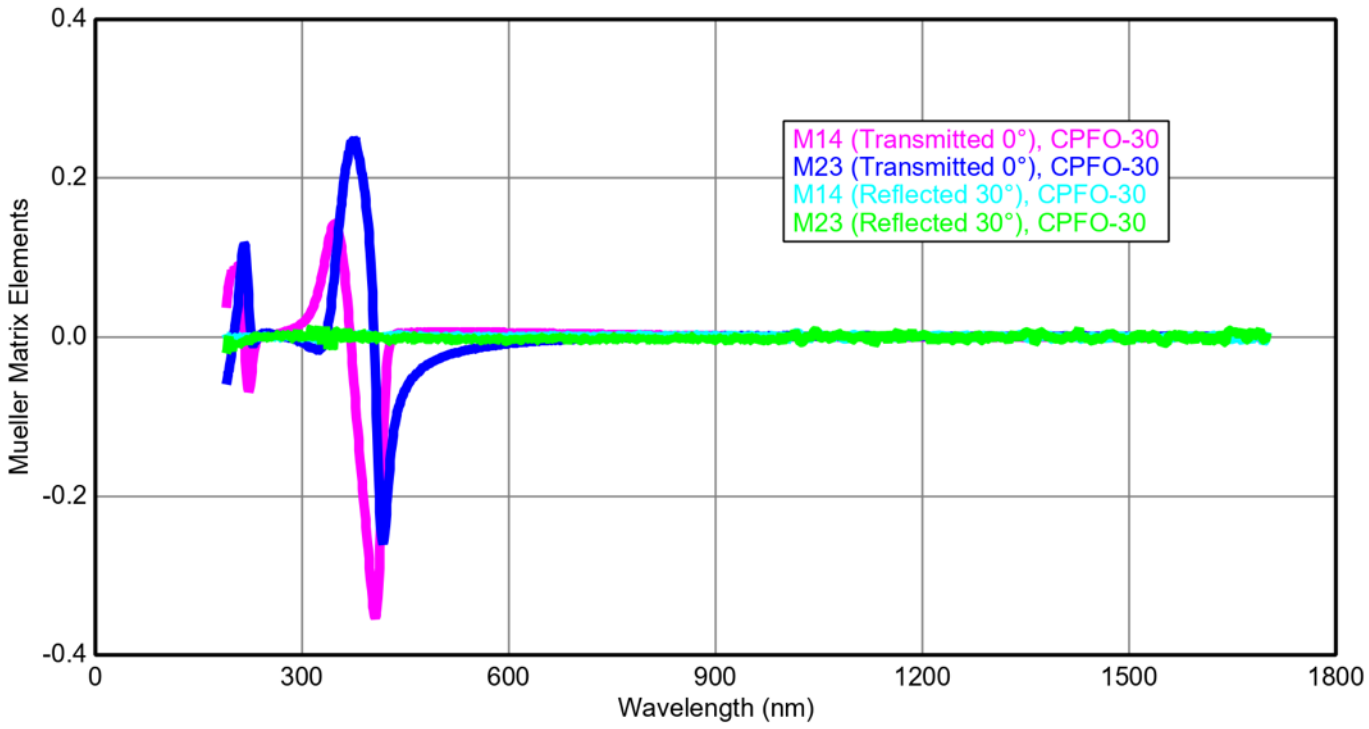

Supplementary Figure 7: Optical models generated for a cholesteric-like structure for ACPCA thin films

Supplementary Figure 7.1. Optical models generated for a cholesteric-stack like structure for F8T2:aza[6]H.

Transmitted Intensity and MM-Transmission data at normal incidence measured from a 130 nm thick F8T2:aza[6]H film were modeled using a cholesteric-stack like structure. The fit parameters included the extraordinary optical function while the ordinary optical function remained the same as from a neat polymer (a). The uniaxial optical axis was tilted into the sample surface plane and then allowed to rotate between the bottom and top of the film. The total rotation in degrees was fit to match the circular features in the data. For this purpose, only the M_{22}, M_{33}, M_{14}, M_{41}, M_{23}, and M_{32} elements were used for fitting (b). The final fit result is shown below: 162° of twist and strong uniaxial anisotropy was able to match the Transmitted data sets.

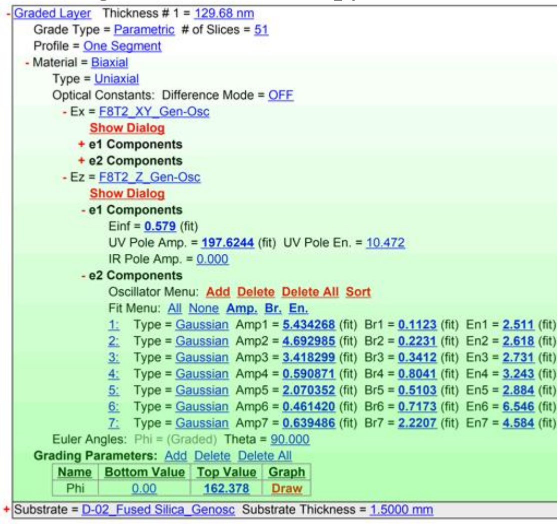

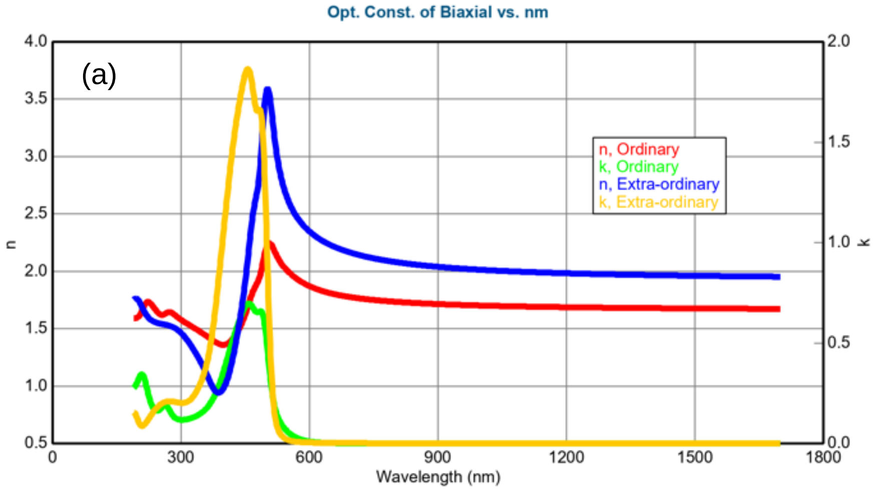

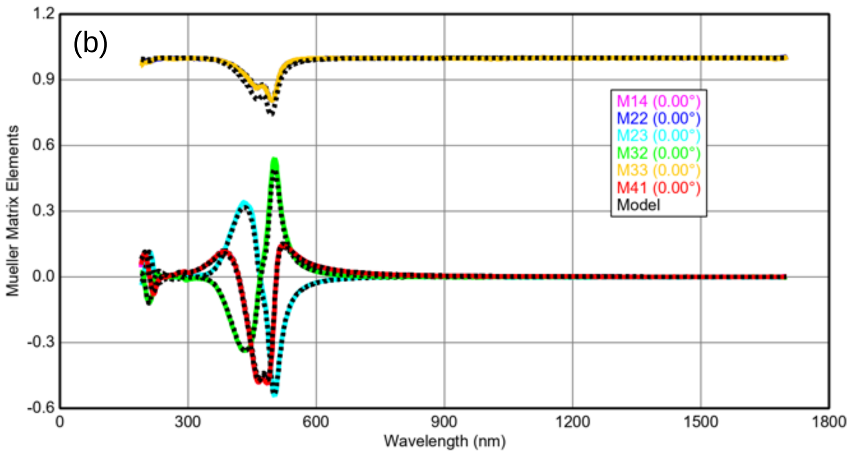

Supplementary Figure 7.2 F8T2:aza[6]H MM-Transmission data and the cholesteric-like optical model at normal incidence.

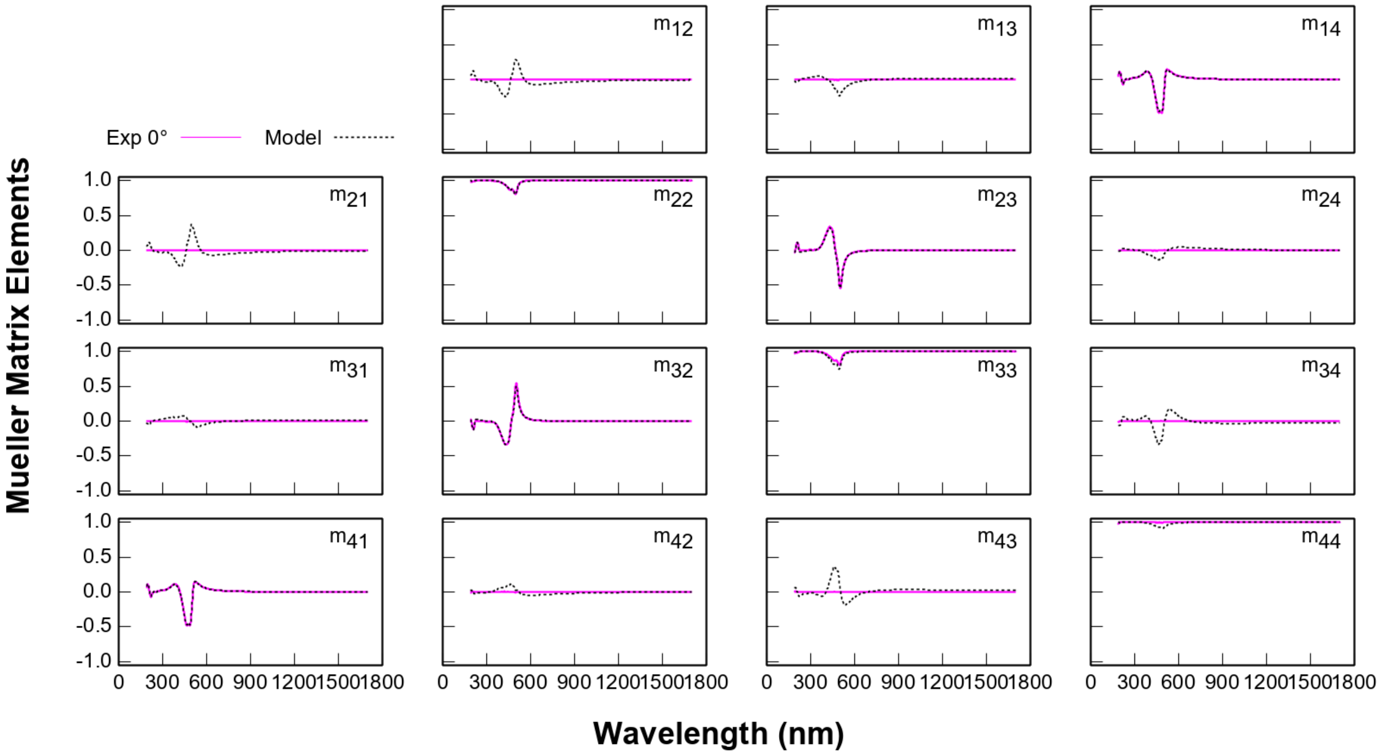

The cholesteric stack structure that can effectively match the circular optical effects (CD and CB) would also produce some linear effects as seen in M_{12}, M_{21}, M_{13}, M_{31}, M_{24}, M_{42}, M_{34}, M_{43}, and even M_{44}. As seen, the measured data does NOT have any of these features in the linear MM elements. Thus, a "twisted" model does not correctly describe the entire MM.

Supplementary Figure 7.3 F8T2:aza[6]H measured MM data in transmission (a) and reflection (b) corresponding to CD (M_{14}) and CB (M_{23}) and a model-calculated match to the MM transmission data assuming the source of the CD and CB is structural.

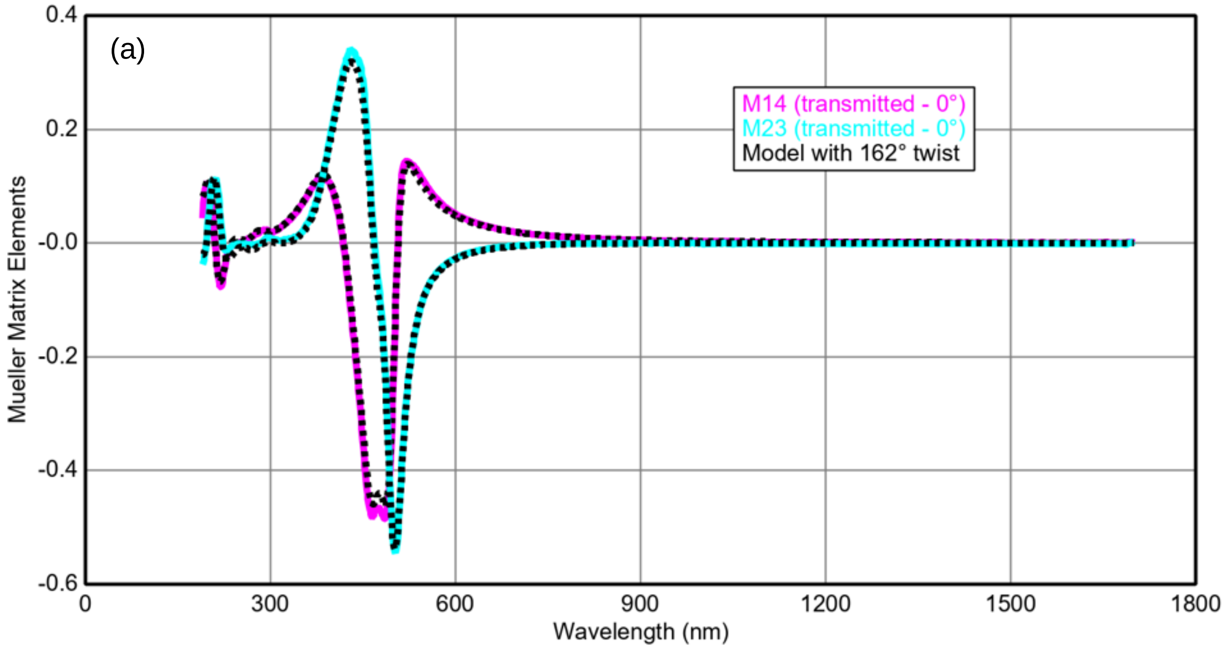

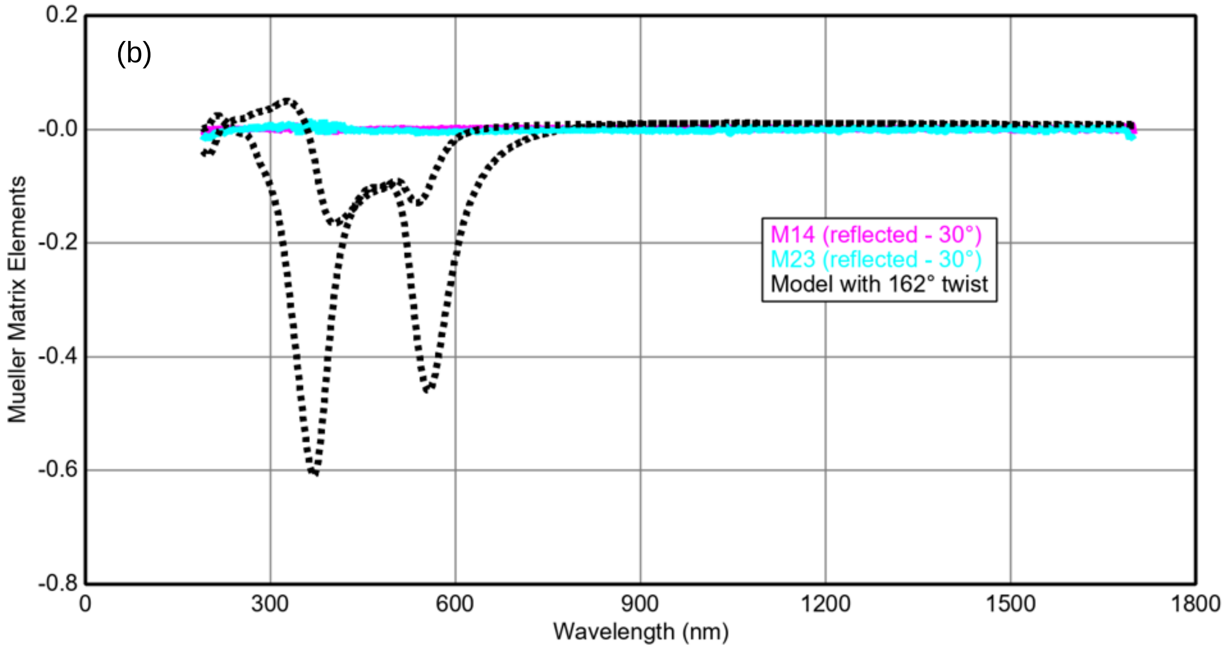

The model uses the ordinary optical functions for F8T2 from the neat polymer and allows the extraordinary values to be fit along with the total twist in the helical structure (modeled here as 0.45 twists). This model is then used to calculate and compare to the reflected MM values from the same sample. As can be seen, the chiral model produces strong CD and CB behavior while our measured data only exhibits a very weak response in reflection.

Supplementary Figure 7.4. Optical models (a, b) generated for a cholesteric-stack like structure for a F8BT:aza[6]H film, following a similar method to Supplementary Figure 4.

- Graded Layer Thickness # 1 = 131.42 nm
  Grade Type = Parametric # of Slices = 51
  Profile = One Segment
  + Material = Biaxial
  Grading Parameters: Add Delete Delete All

**Name**	**Bottom Value**	**Top Value**	**Graph**
Phi	0.00	**154.772**	**Draw**

+ Substrate = EMA Substrate Thickness = 1.5000 mm

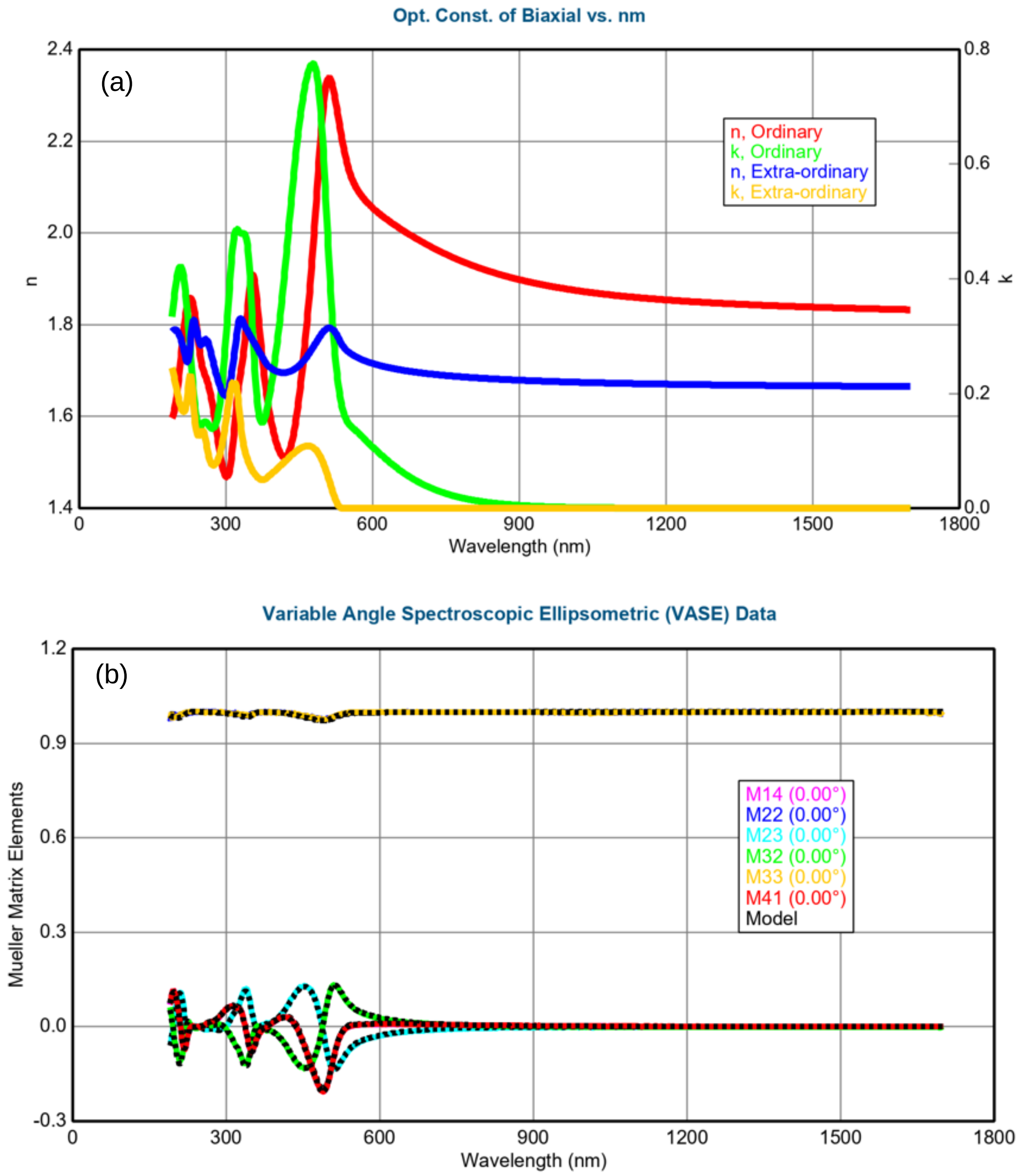

Supplementary Figure 7.5. F8BT:aza[6]H MM-Transmission data and the cholesteric-like optical model at normal incidence.

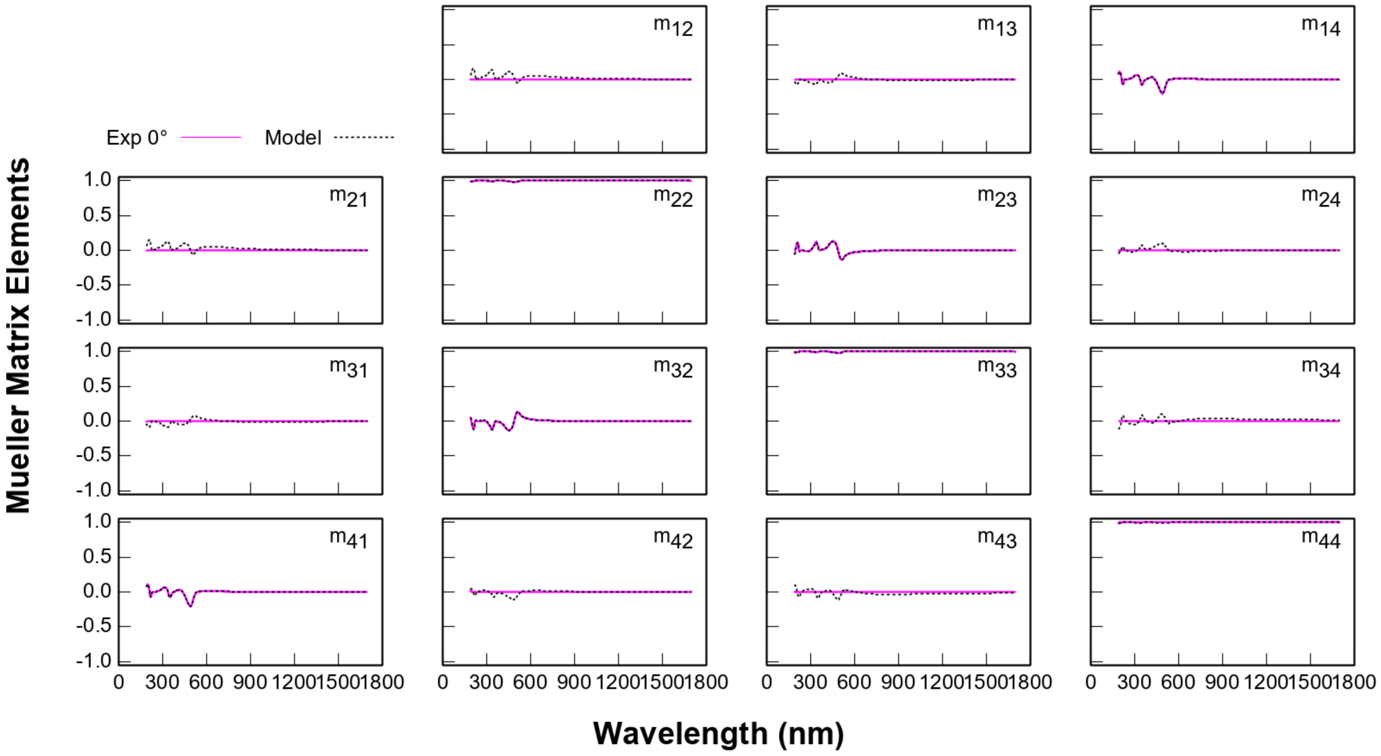

The cholesteric-like stack structure that can effectively match the circular optical effects (CD and CB) would also produce some linear effects as seen in M_{12}, M_{21}, M_{13}, M_{31}, M_{24}, M_{42}, M_{34}, M_{43}, and even M_{44}. As seen, the measured data does not have any of these features in the linear MM elements.

Supplementary Figure 7.6 F8BT:aza[6]H measured MM data in transmission (a) and reflection (b) corresponding to CD (M_{14}) and CB (M_{23}) along with a model-calculated match to the MM transmission data assuming the source of the CD and CB is structural (155° total twist).

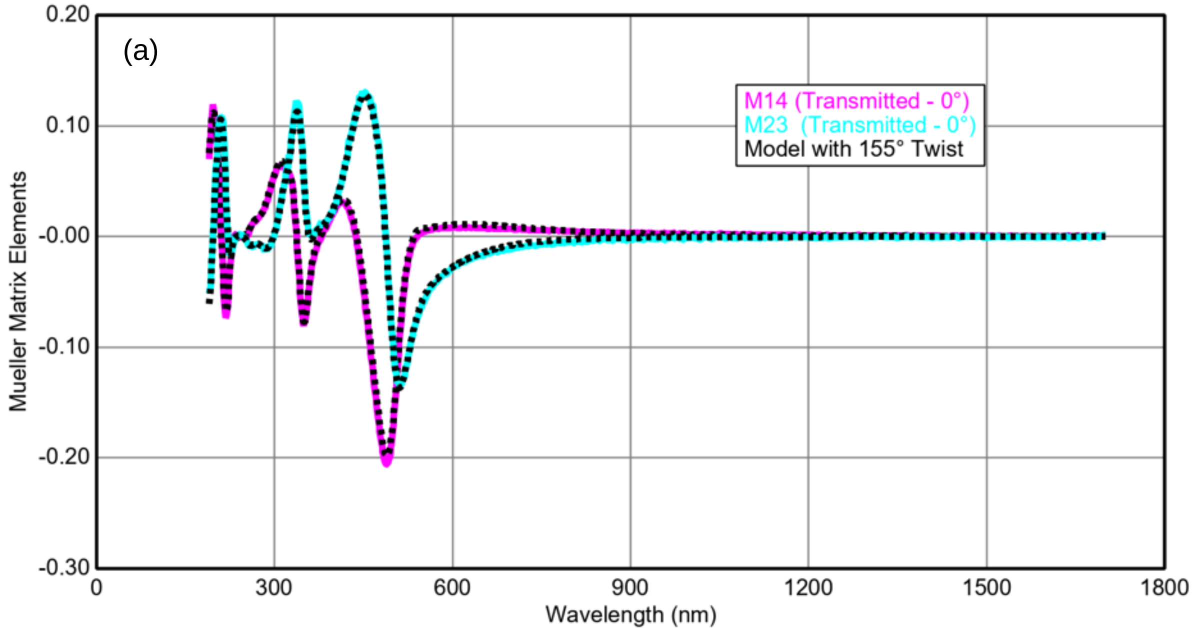

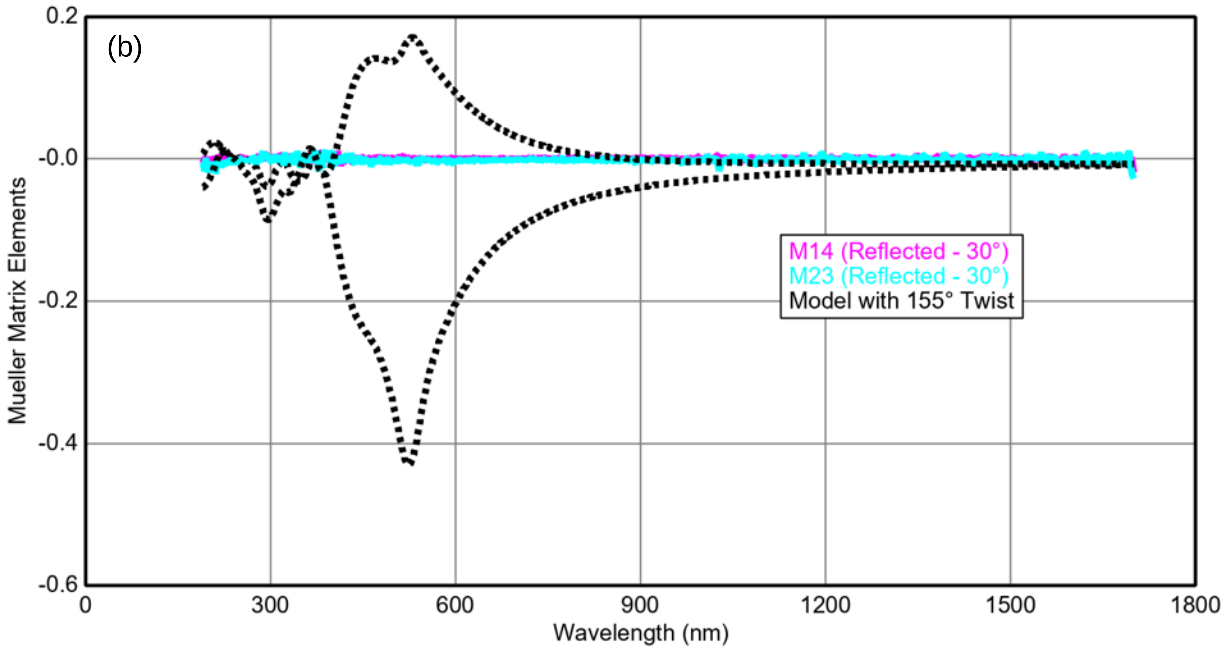

This model is then used to calculate and compare to the reflected MM values from the same sample. The cholesteric stack model produces strong CD and CB behavior while our measured data only exhibits a very weak response in reflection.

Supplementary Figure 7.7. Optical models generated for a cholesteric-stack like structure for a film of PFO:aza[6]H.

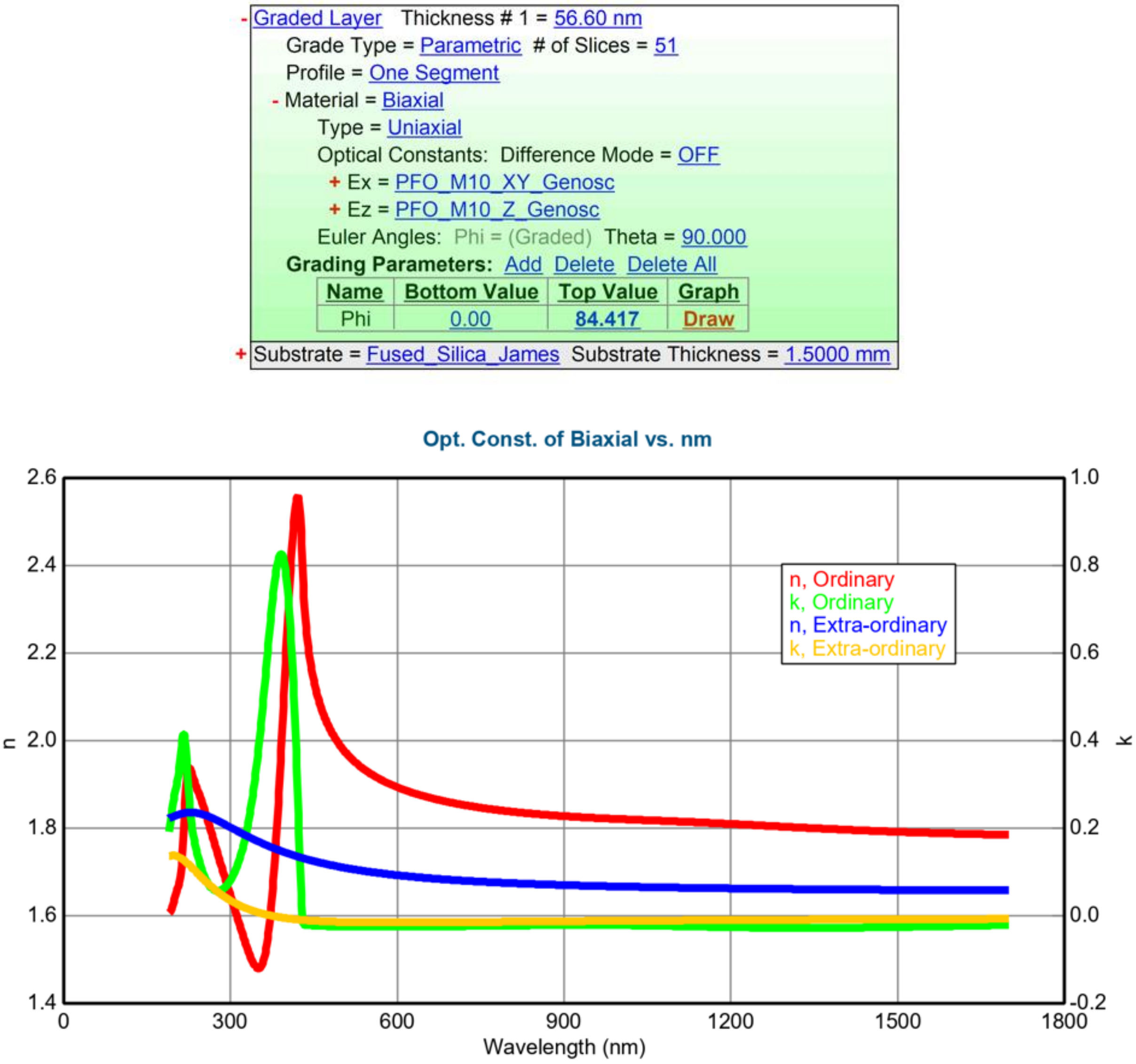

Supplementary Figure 7.8. PFO:aza[6]H MM-Transmission data and the cholesteric-like optical model at normal incidence.

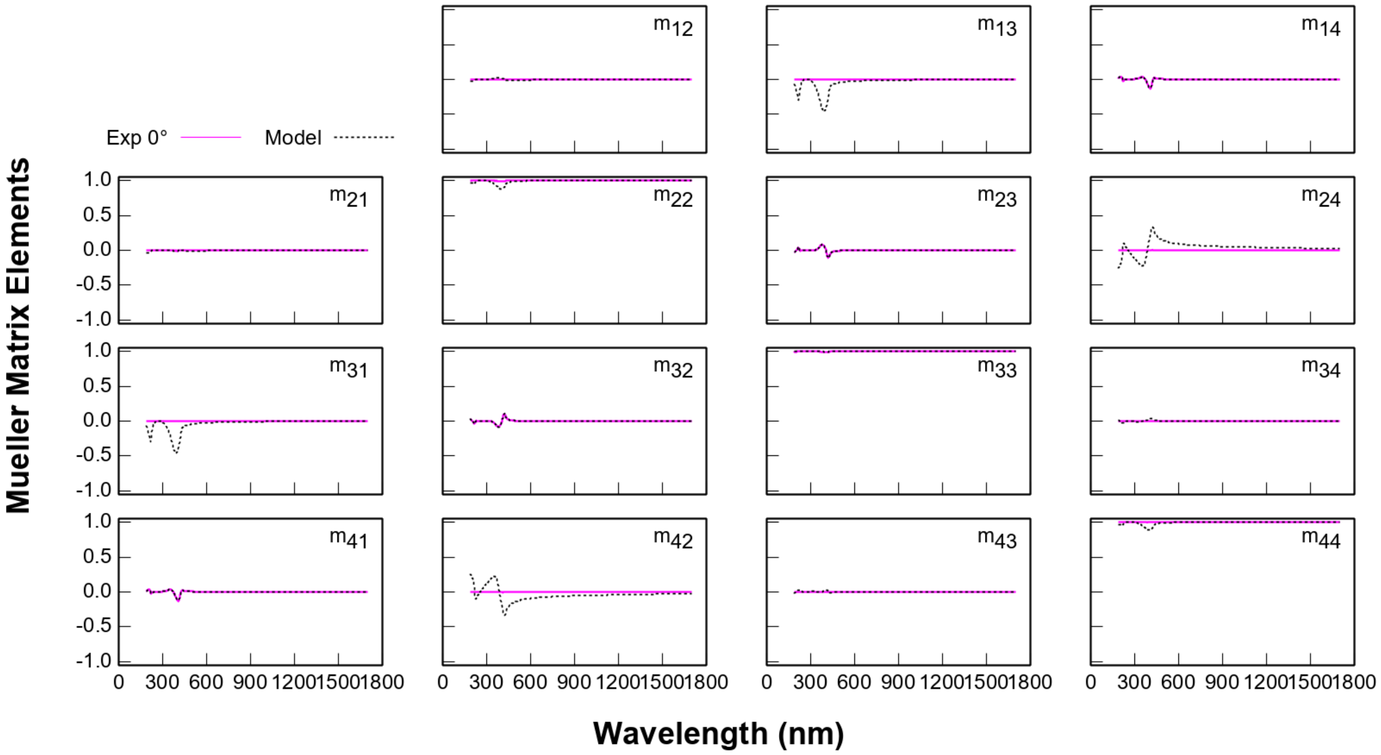

The chiral structure that can effectively match the circular optical effects (CD and CB) would also produce some linear effects as seen in M_{12}, M_{21}, M_{13}, M_{31}, M_{24}, M_{42}, M_{34}, M_{43}, and even M_{44}. As seen, the measured data does NOT have any of these features in the linear MM elements.

Supplementary Figure 7.9 PFO:aza[6]H measured MM data corresponding to CD (M_{14}) and CB (M_{23}) along with a model-calculated match to the MM transmission (a) and reflection (b) data assuming the source of the CD and CB is structural (85° total twist).

This model is then used to calculate and compare to the reflected MM values from the same sample. The cholesteric stack model produces strong CD and CB behaviour while our measured data only exhibits a very weak response in reflection.

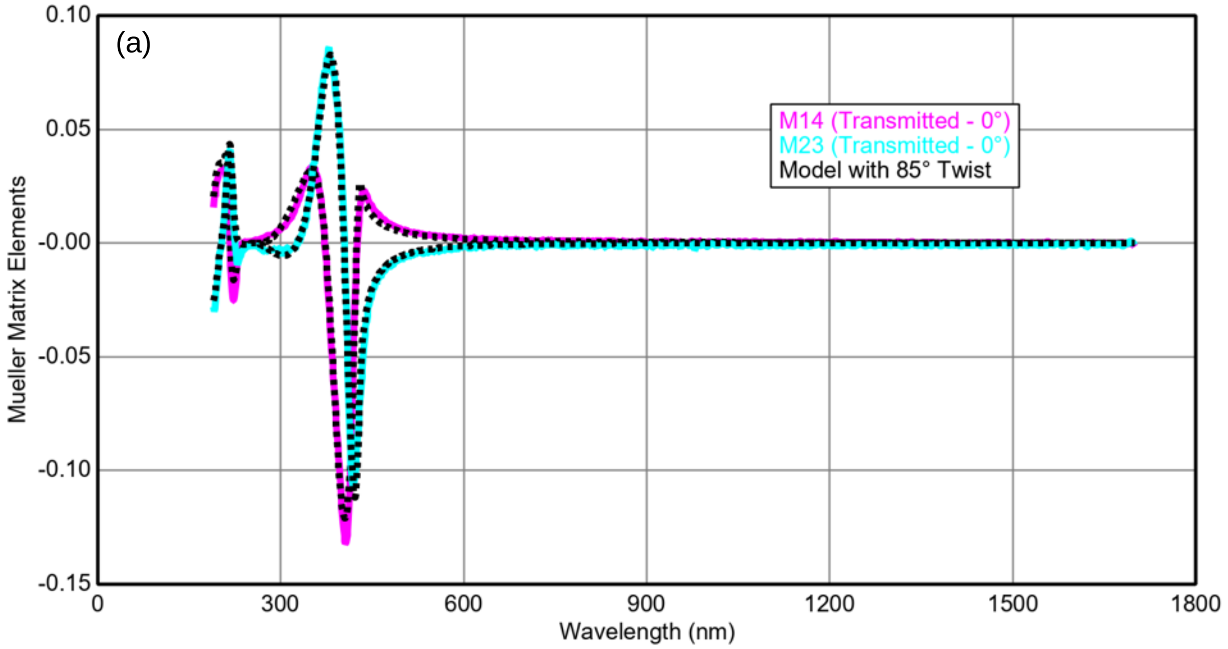

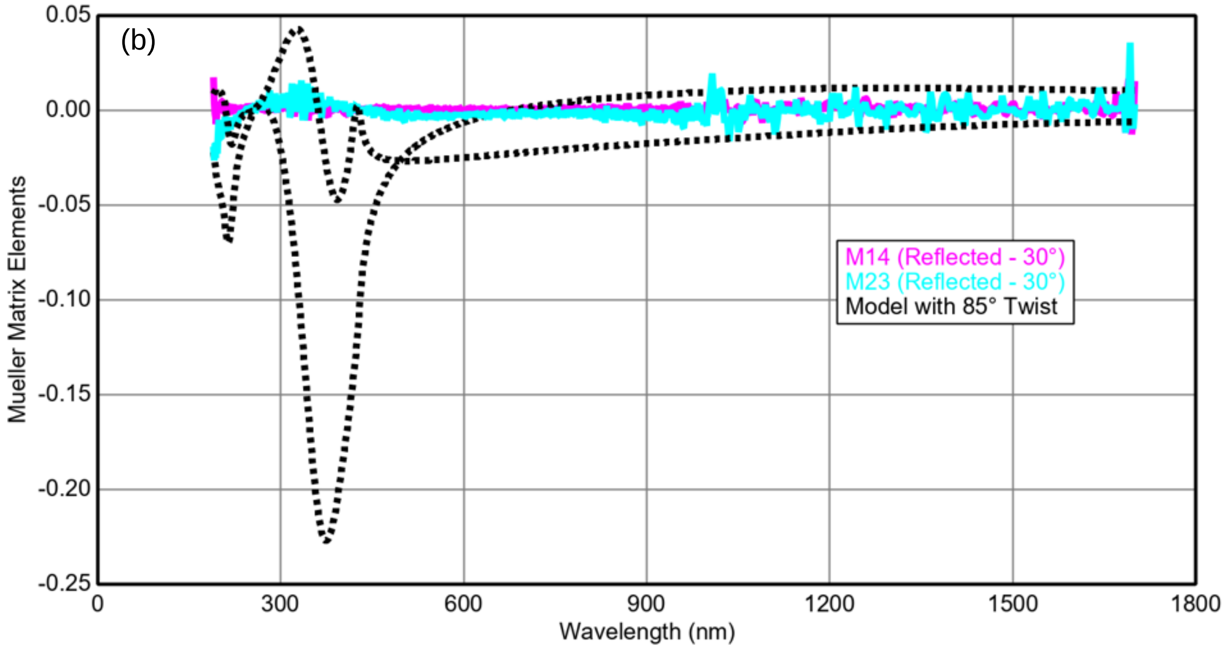

Supplementary Discussion 2 Multi-domain cholesteric model

Alongside investigating the optical response of a single domain cholesteric model, two multi-domain models were generated, and the Mueller Matrix evaluated as an (1) an incoherent (domains > the wavelength of light) and (2) a coherent (domains < the wavelength of light) superposition. We should note, at present there are no experimental control samples to validate the outputs of either model. We find that the coherent superposition (2) of randomly oriented, sub-micron, partially formed cholesteric domains (i.e. not fully complete twists) **can** result in MMSE spectra without circular elements, but only at certain angles of incidence. The model does not hold when multiple angles of incidence are considered, and we see no evidence of such grains through spatially resolved MMSE/CD. Therefore, we do not believe that a multi-domain cholesteric structure is present in the unaligned polyfluorene systems considered here.

Incoherent superposition of 'large' domains

To establish whether a multi-domain or 'mosaic' cholesteric liquid crystal structure could be responsible for the strong chiroptical phenomena recorded here, a model was created that allowed mixing of multiple domains with ability to vary their 1) starting orientation, 2) amount of twisting, and 3) tilt angle. Of these three effects, calculations with multiple "starting orientations" can effectively minimize the linear response for transmitted measurements, while maintaining the circular response.

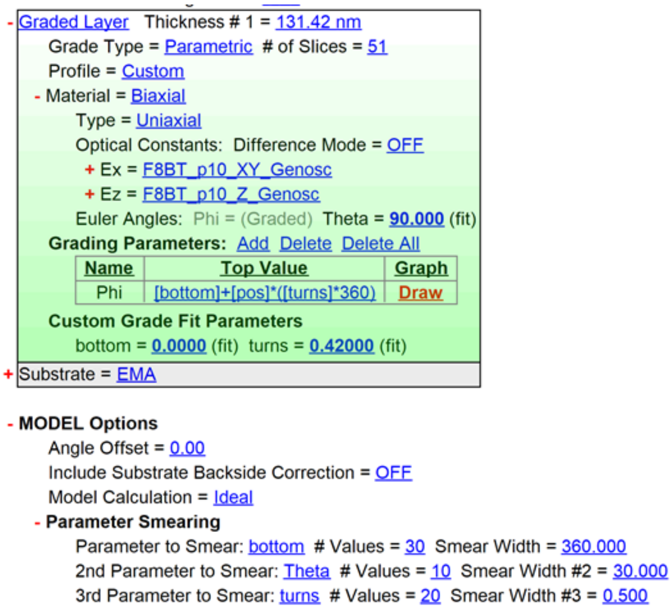

Details of the multi-domain model that modifies for starting orientation.

The multi-domain model uses an incoherent summation of calculated Mueller matrices (M_k) corresponding to individual systems with varied properties. For example, smearing of the bottom orientation was demonstrated by calculating 30 different Mueller matrices, each with a different starting molecular orientation (Phi Euler angle $\phi(n)$), with a total range of 360°. These 30 different Mueller matrices are combined incoherently. Our model also calculated the variation of two additional model parameters– the tilt axis of the liquid crystal and the total number of twists. The detected Mueller matrix (M_{det}) is a weighted summation of the individual Mueller matrices (Supplementary Equation 8):

$$M_{det} = \sum_k i_k \ddot{M}_k \qquad \text{Supplementary Equation 8}$$

$$\sum_k i_k = 100\%$$

The mixing of multiple Mueller matrices with different values inherently leads to considerable depolarization, which would have been measured from a "mosaic" multi-domain structure with domain sizes larger than the coherence length of the light source (~ domains larger than the wavelength).

MMSE spectra recorded in transmission and a model that makes use of a (a) single- and (b) multi-domain cholesteric stack with 15 different starting angles.

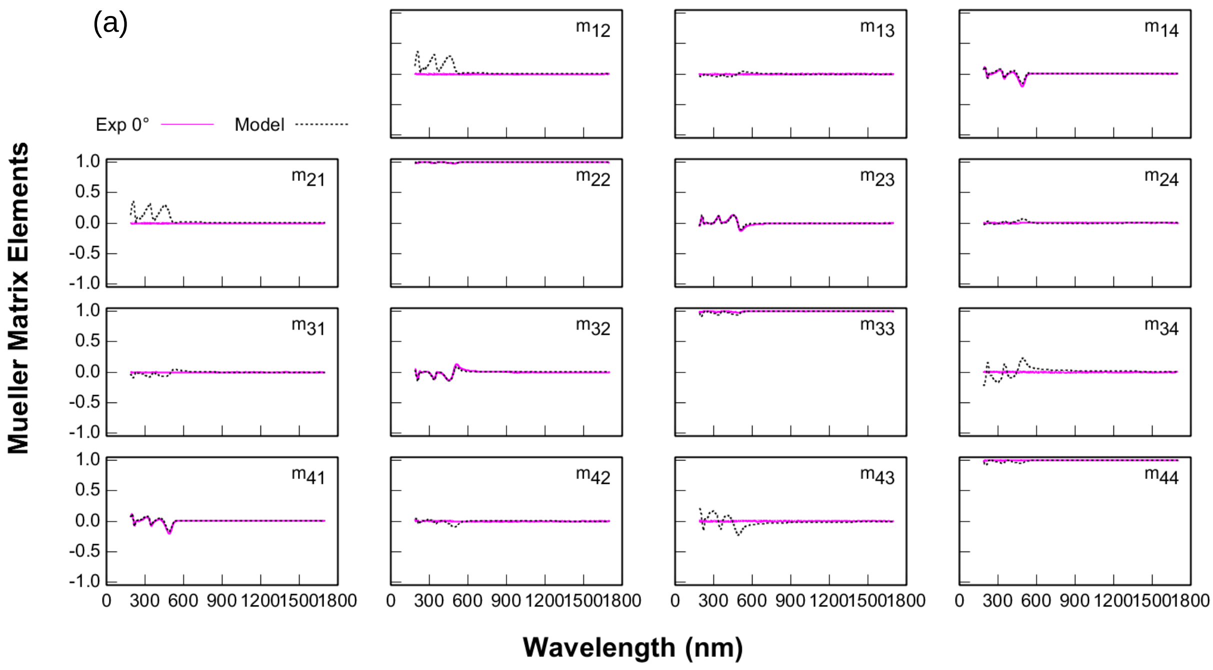

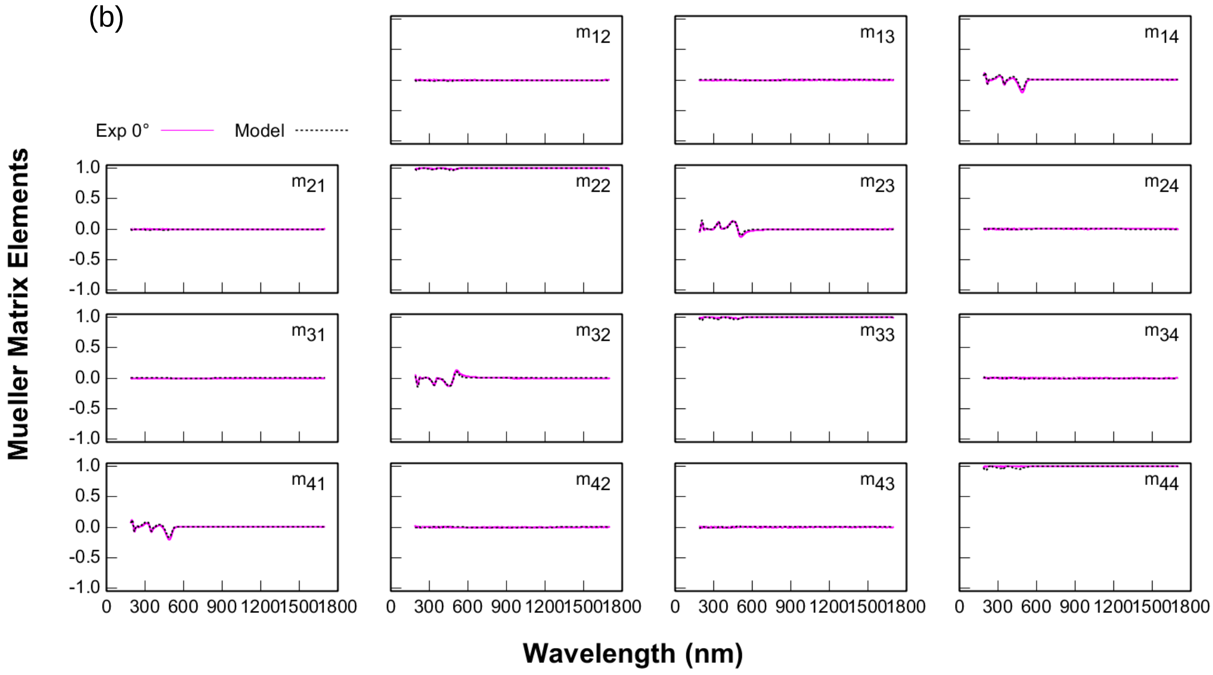

Measured **transmission depolarisation index** (pink line) and the predicted depolarisation index for single (black line) and multi-domain (blue line) systems.

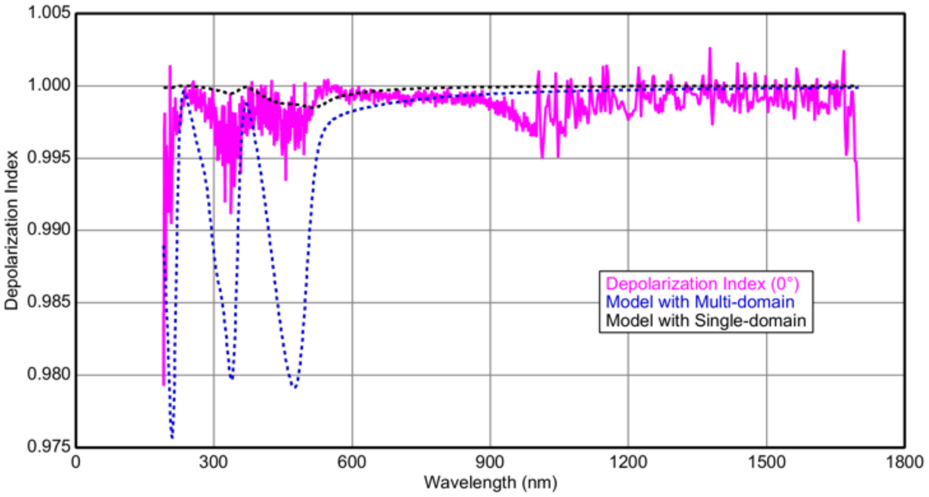

Whilst a multi-domain model that only makes use of multiple starting orientations can minimise the linear terms of transmission measurements, it does not suppress the circular response of the reflection spectra. Instead, we found a combination of all three (i.e. a calculation that allows for starting orientation, amount of twist, and tilt angle) ***can*** suppress the circular response. However, it would suppress the circular response for both transmitted and reflected beams and would lead to significant depolarization.

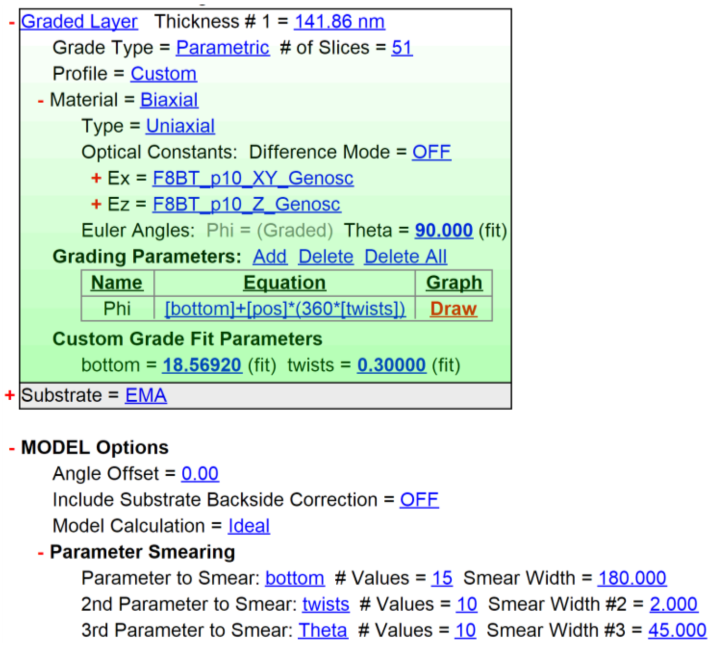

Details of the multi-domain model that modifies for starting orientation, amount of twist and tilt angle.

Reflection M14 (CD) and M23 (CB) terms predicted using the multi-domain model.

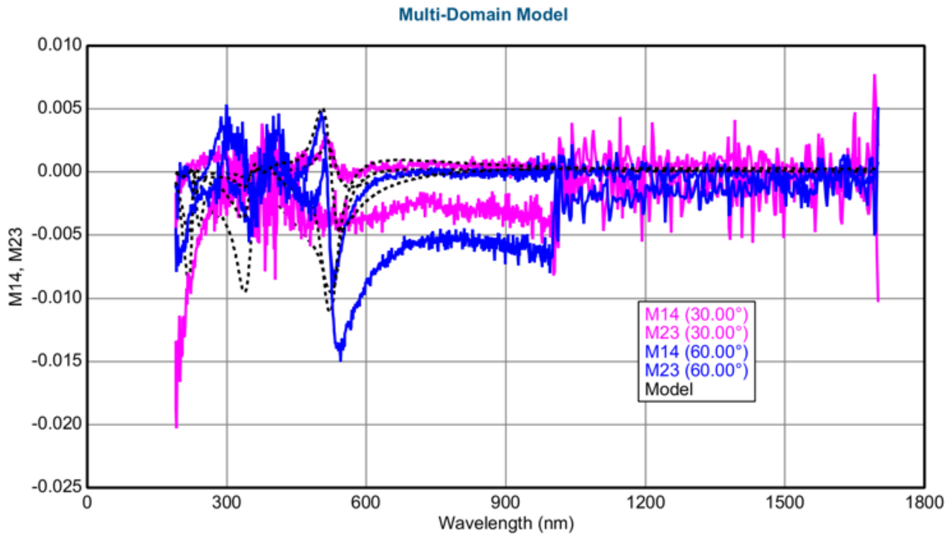

Measured **reflection depolarisation index** (pink and blue lines) and the modelled depolarisation index for a single (a) and multi-domain (b) cholesteric stack.

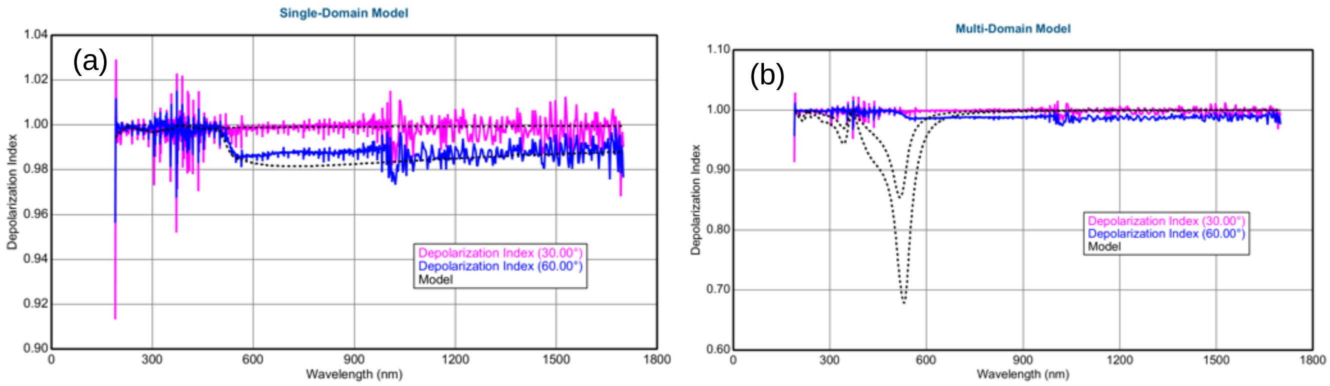

Transmission MMSE spectra recorded for annealed F8BT:aza[M] films and the predicted spectra from a model that controls for the 1) starting orientation, 2) amount of twist, and 3) tilt angle.

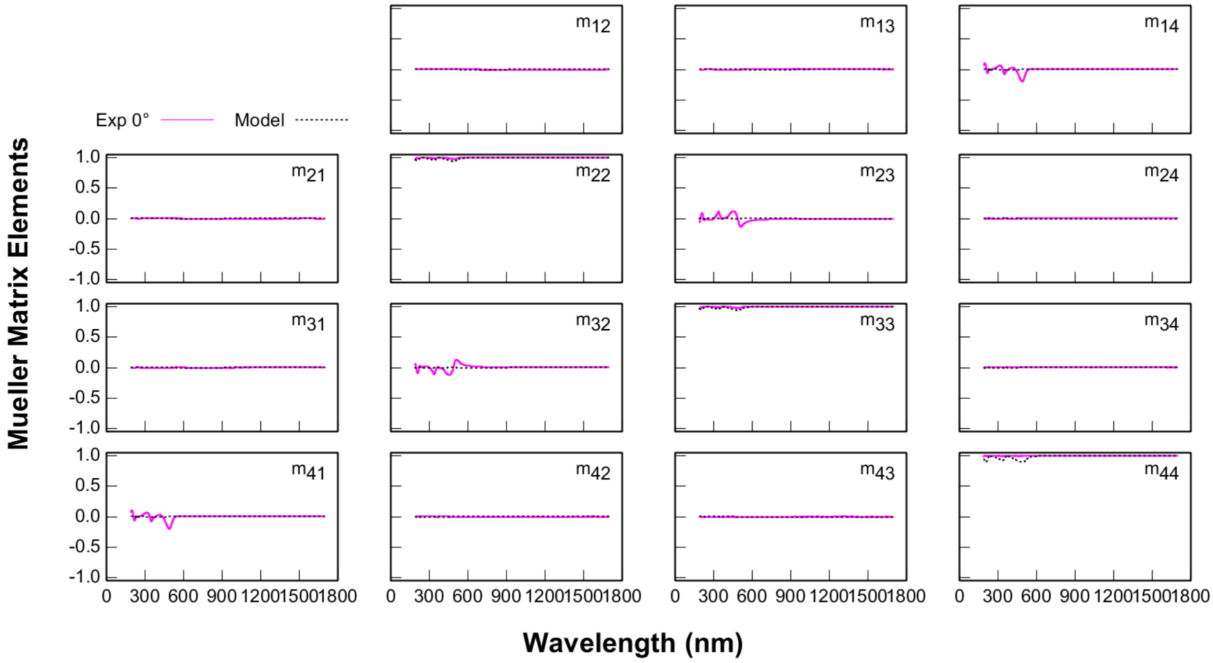

In summary, we have not been able to find a model based on a multi-domain system that simultaneously satisfies all the experimental observations: 1) suppress the **linear anisotropic responses** that would be obtained in transmission MMSE measurements, 2) suppress the **circular anisotropic responses** that would be obtained in reflection MMSE measurement, 3) maintain the chiroptical response in transmission (not only at normal incidence but also at oblique incidence) and 4) not introducing **significant depolarization**.

Coherent superposition of 'small' domains

Having failed to successfully simulate the optical response using a multi-domain cholesteric model, a model was created that describes the optical response of a coherent superposition of multiple grains of not fully developed twisted systems (i.e. cholesteric stacks of heights much smaller than a full pitch) with random orientations (see right).

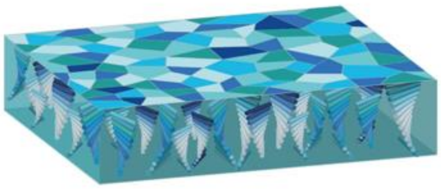

A cartoon of the sub-micron multi-domain cholesteric system considered in this model, which involves a coherent superposition of randomly oriented grains.

The model makes use of the Woollam WVASE® General Multi-model Patterning Layer which allows either coherent or incoherent mixing of multiple models (which refers to the individual grains). The coherent mixing of multiple grains is described by the weighted sum of multiple (up to 5) Jones matrices:

$$J = \begin{pmatrix} j_{11} & j_{12} \\ j_{21} & j_{22} \end{pmatrix} = \sum_n c_n \begin{bmatrix} r_{pp_n} & r_{sp_n} \\ r_{ps_n} & r_{ss_n} \end{bmatrix}$$

Supplementary Equation 9

where c_n is a weighting factor associated with each individual grain (n^{th} Jones matrix) and r_{pp}, r_{sp}, r_{ps}, and r_{ss} represent the complex Fresnel coefficients for the nth model.

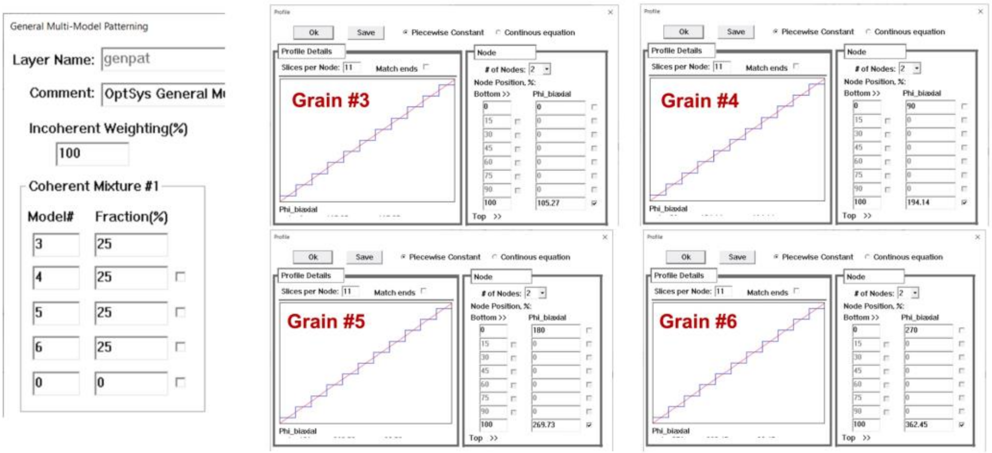

4	Graded (biaxial)	111.304 nm
3	biaxial	0.000 nm
2	GenOsc_Z_p10	0.000 nm
1	GenOsc_XY_p10	0.000 nm
0	fused silica	1.5 mm

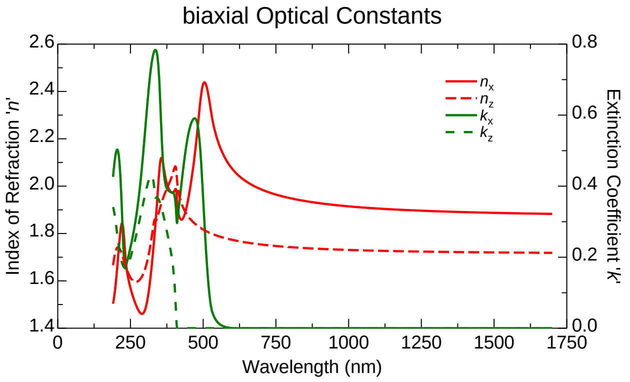

Above we describe the general approach that underpin this multi-domain analysis; the coherent mixing of 4 grains – where each model represents a grain with a twisted anisotropic structure and a different starting orientation. For each grain, the total amount of twist can vary along with film thickness and anisotropic optical constants to fit the MM data in reflection (25°) and transmission (0°). Anisotropic optical constants were allowed to vary in both directions but constrained by Kramers-Kronig consistent oscillator summations.

Below we show the MMSE data collected in transmission (MMt, angle of incidence 0°) and reflection (MMr angle of incidence 25°) for a F8BT:aza[*M*] film alongside the coherent multi-domain cholesteric model proposed above. We then focus on the **transmitted** (a) and **reflected** (b) circular responses (M_{14} and M_{23}) for a single model (grain) and the situation when four grains are combined coherently. Finally, we consider the circular response (M_{14} and M_{23}) in reflection for a single model (grain) using the same total twist (105°) but with different starting orientations. If the data averaged more grains of one orientation than another, then cancellation would not be complete, and we would see a preferential circular response in reflection.

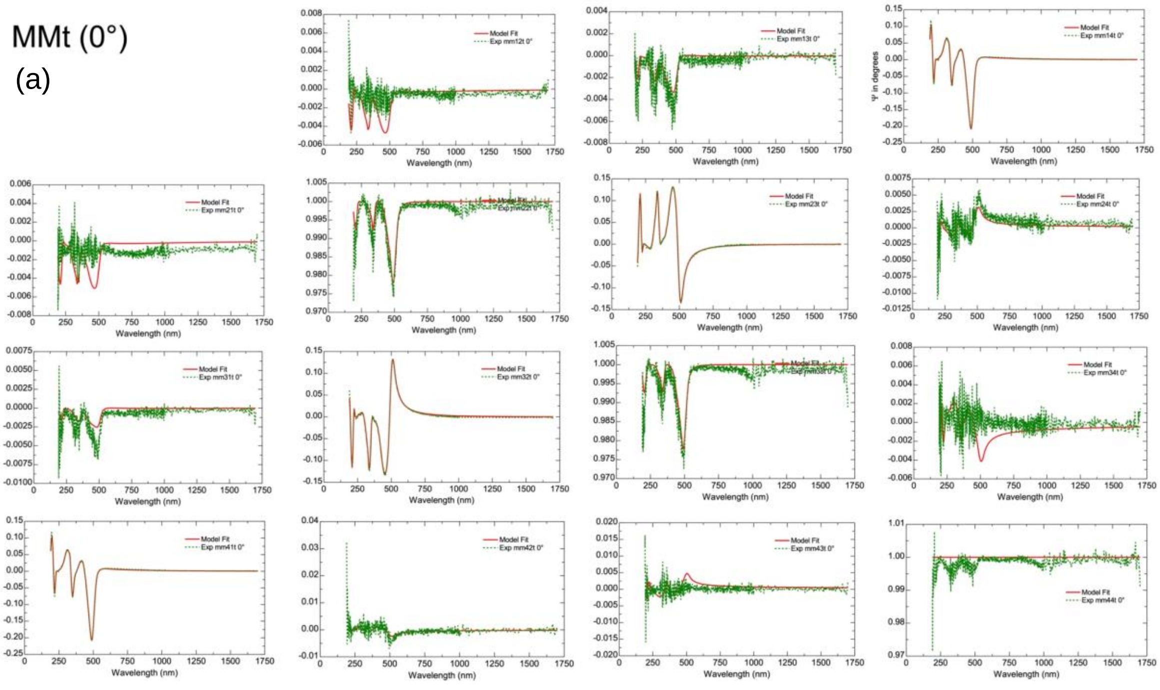

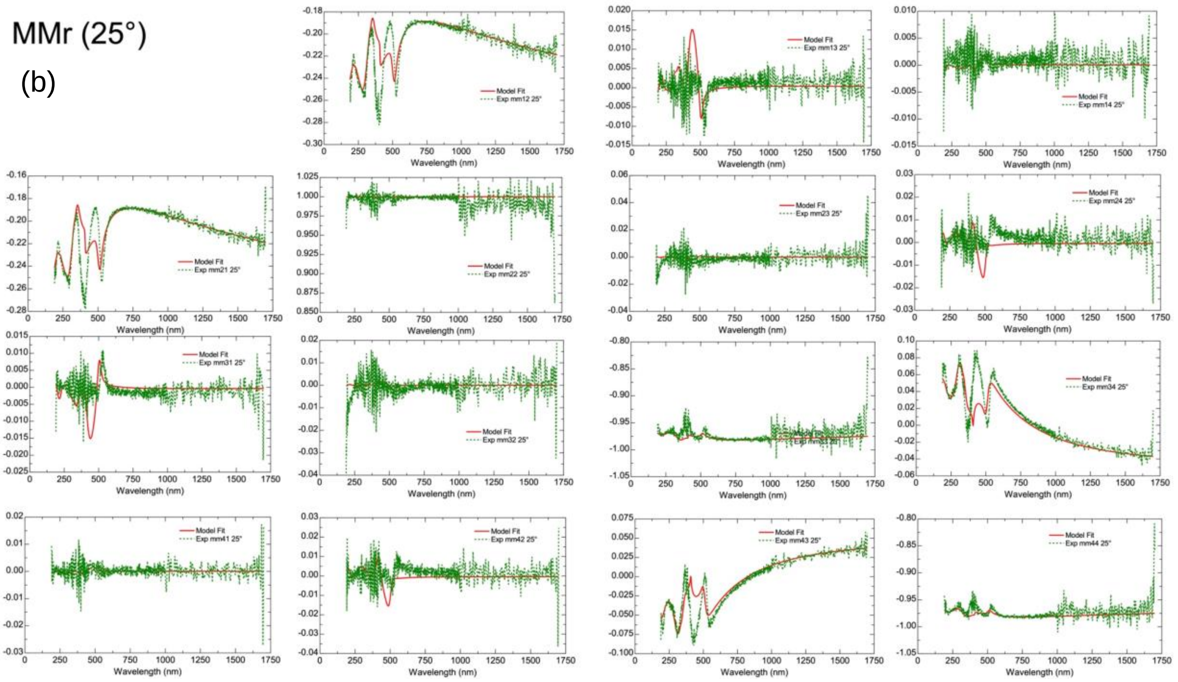

Transmitted and reflected circular response for individual ('model 3') and coherently combined ('model 3 + 6') grains.

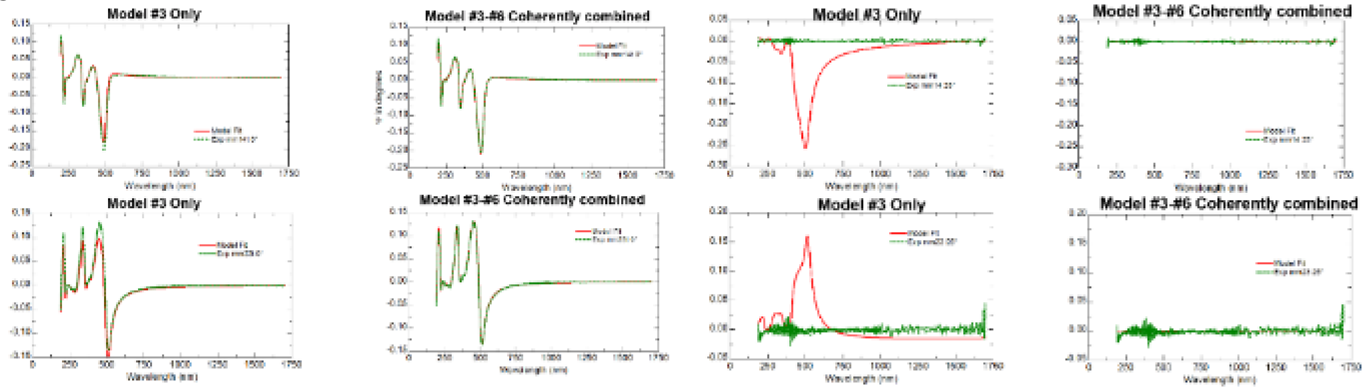

Transmitted circular response (M_{14} and M_{23}) **Reflected circular response (M_{14} and M_{23})**

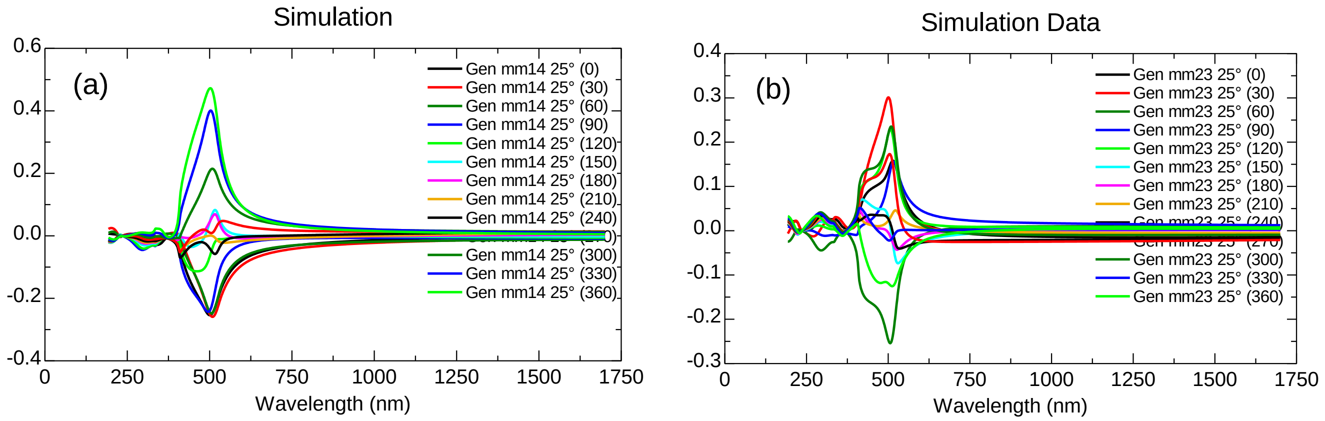

The circular response (M_{14}, (a) and M_{23}, (b)) in reflection for a single grain.

Based on this data, we can draw the following conclusion. For the cancellation to be effective, the twisted structures need to "balance" each other out with complementary starting orientations. For example, a starting

orientation of 0° can be balanced with a starting orientation of 90°. It is very unlikely that there are a small number of starting orientations – especially since the in-plane anisotropy of this twisted model also produces linear optical responses which need to be smeared out (in addition to the circular responses). It is more likely that the coherent models would be a collection of random orientations and possibly random tilt angles relative to the surface.

Informed by the coherent multi-domain model described above, we compared the simulated and experimental transmission data at variable angles (transmission, MMt; 0°, 30° and 60° and reflection, MMr; 25° and 60°). If multiple angles of incidence are considered; the coherent multi-domain model fails to fit all data together. This implies that this multidomain model is not compatible with the overall uniaxial response seen on the sample.

Experimental and simulated transmission (a) and reflection (b) MMSE spectra acquired at multiple angles of incidence

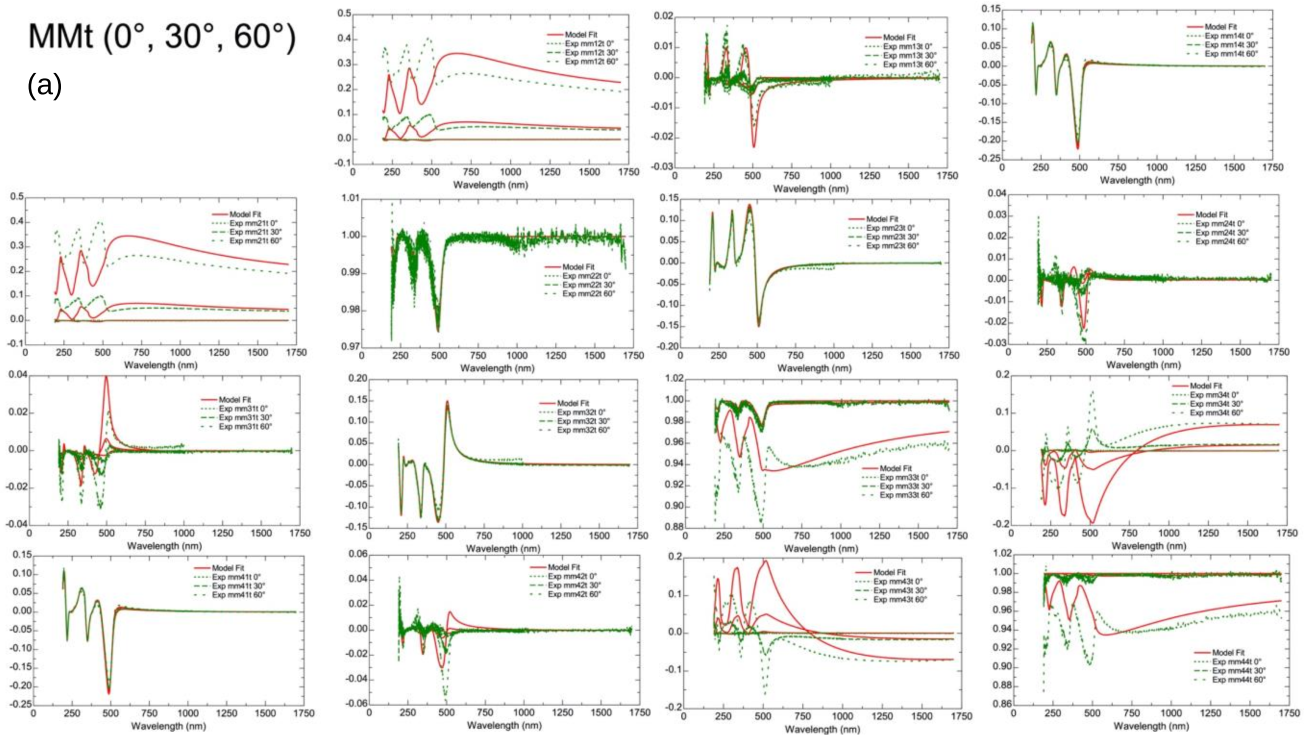

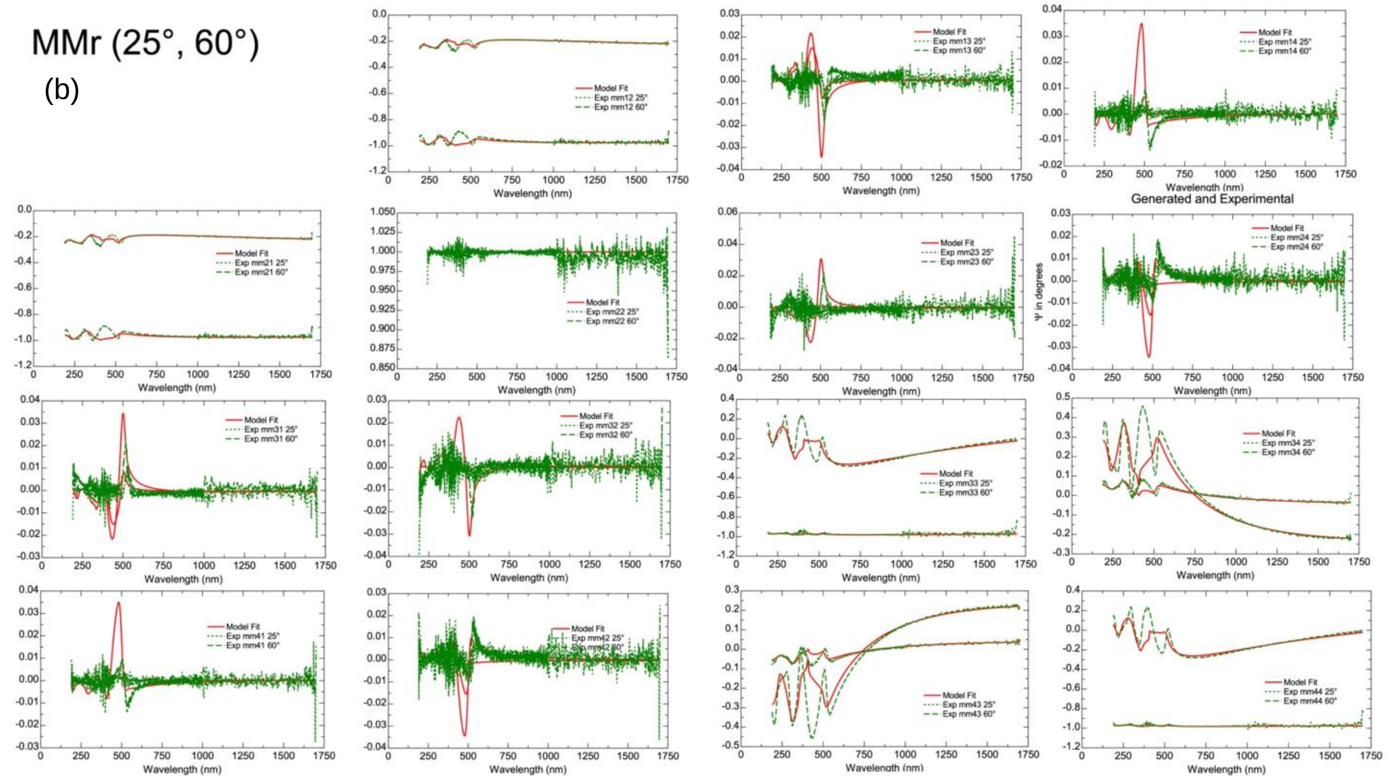

In order to further experimentally investigate whether the optical response from such a multi-domain structure exists within our samples, we mapped the uniformity of the circular terms in reflectance (65°) using a 40 μm beam over 1200 points on a 0.8 mm × 0.8 mm area. Whilst this is by no means a sub-micron measurement, if the grain sizes are in the micron-range then such a focused measurement should show varying circular effects when not perfectly “cancelled”. The grey circles (the vertical line along x = 0) were selected to compare spectra.

As can be seen below, there is no indication of large grains such as those described above (the circular response of single grains) where the circular response would not maintain “balance” at different locations. In other words, any proposed multi-grain model would need to contain a large number of smaller grains to smear-out the circular reflected response in a similar manner at each of these points.

Supplementary Figure 8 *Spatially resolved circular terms measured in reflectance*

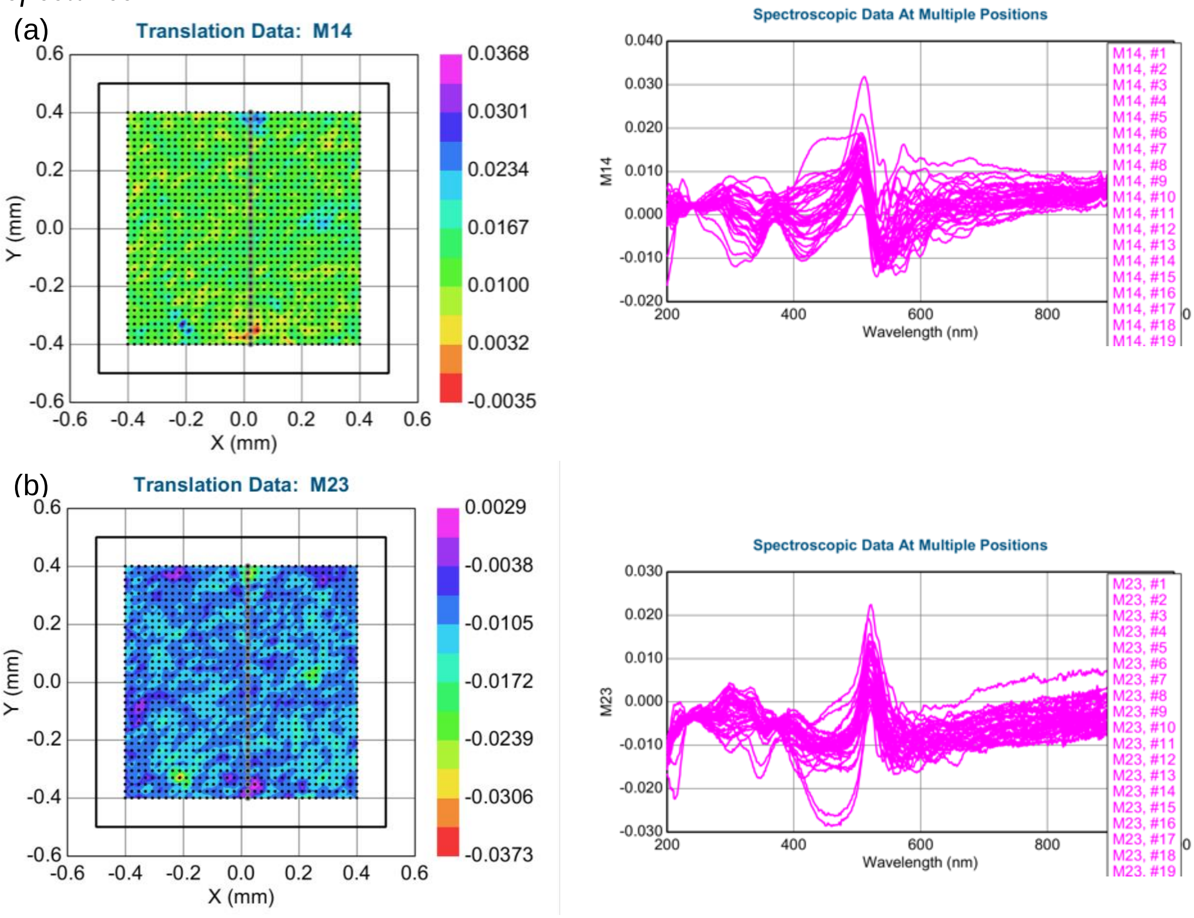

Spatially resolved circular terms (M14, (a) and M23, (b)) measured in reflectance for an F8BT:aza[M] thin film.

Comparable to the spatially resolved CD spectra acquired at the Diamond Light Source (Supplementary Figure 2), the response is incredibly uniform. In fact, the new, high resolution, spatially resolved spectroscopic data above perfectly fits the 6 × 6 model described in Equation 2 which incorporates magneto-electric coupling (i.e., natural optical activity).

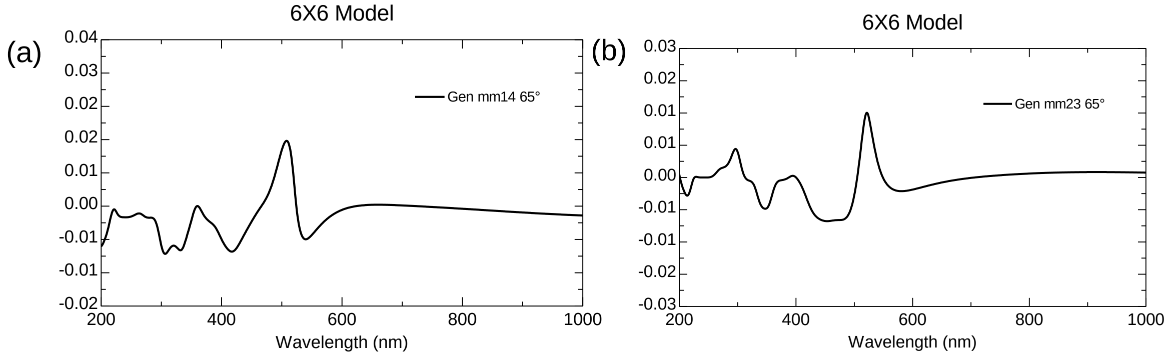

Simulated circular terms (a, MM_{14}, B, MM_{23}) spectra in reflection (65°) using the 6 × 6 magneto-electric coupling model

We have also measured transmission and reflection MMSE data for ultra-thick films (915 nm), which should be closer to a ‘Bragg reflector’ (Figure 10). Even at these thicknesses, there is no circular selective response in reflection.

(a) Transmission (0°) **(b) Reflection (15°)**

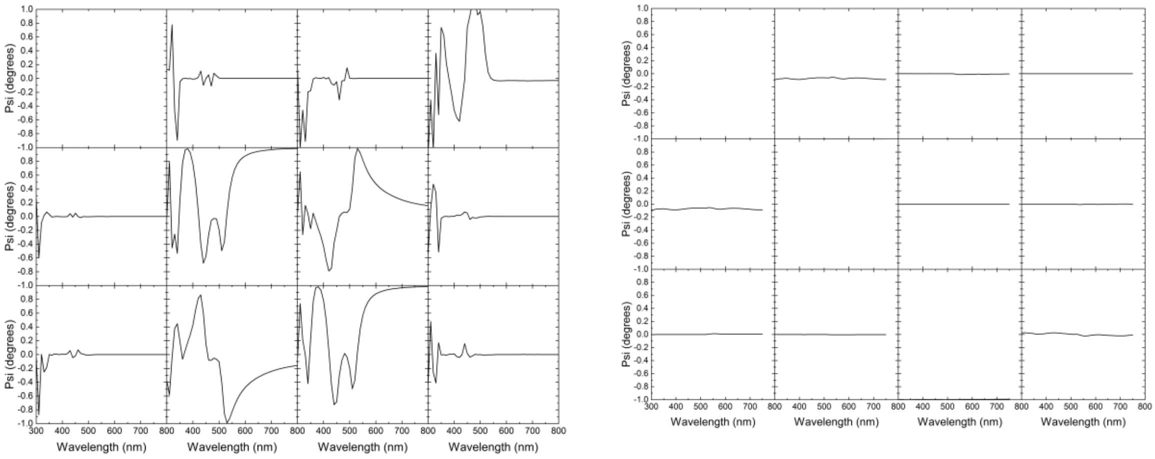

Transmission (a) and reflection (b) MMSE spectra recorded for a 915 nm thick F8BT:aza[6]H (P) film

Supplementary Figure 9 Anisotropic gyrotropic terms for annealed ACPCA thin films

We note that a model based on an isotropic optical activity tensor (i.e. $\alpha_x \equiv \alpha_z$) cannot explain well the results obtained at oblique angles. This fact becomes especially evident when trying to fit simultaneously transmission measurements at both normal incidence (0°) and at the most oblique measurement angle (60°) as shown below:

PFO:aza[*M*]H:

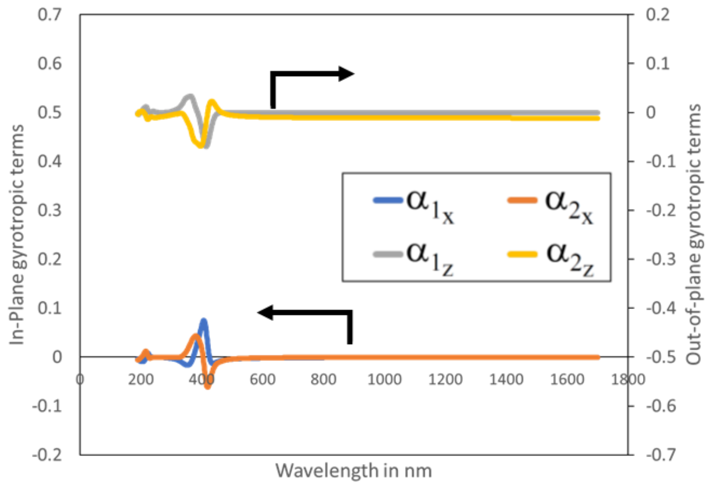

F8BT:aza[*M*]H:

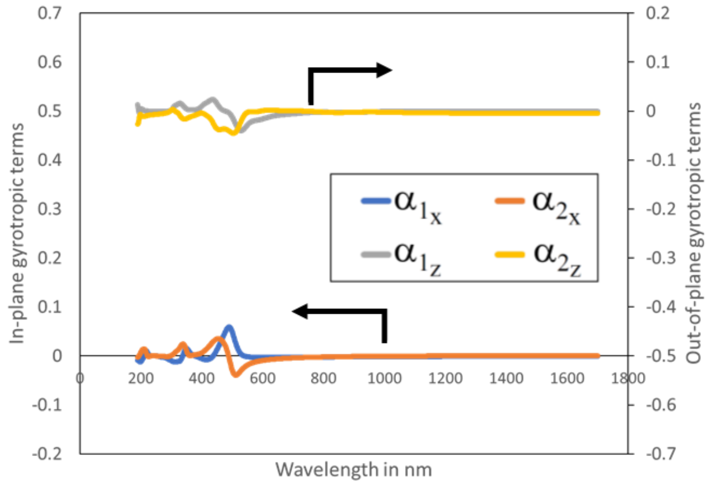

F8T2:aza[*M*]H:

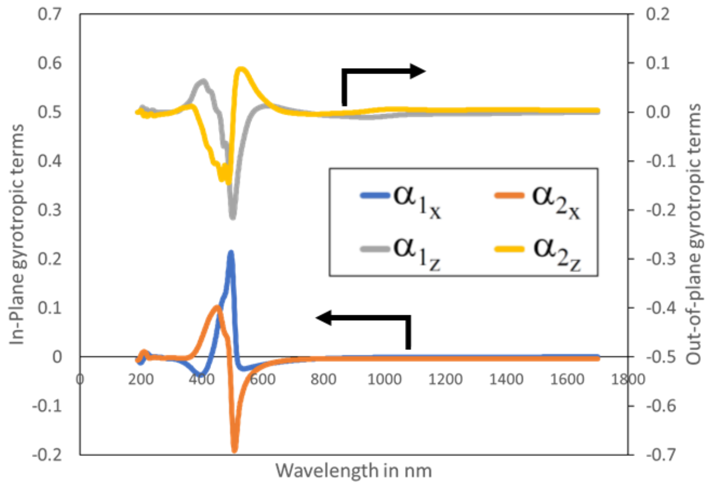

Supplementary Figure 9.1 Measured MMSE data in transmission for 90 nm thick F8T2:aza[M]H film on glass with model-fits assuming (a) isotropic optical activity and (b) uniaxial optical activity.

Variable angle MMSE in transmission helps gain sensitivity to the uniaxial nature of the optical activity.

(a) Fit to M41 and M23 using isotropic alpha

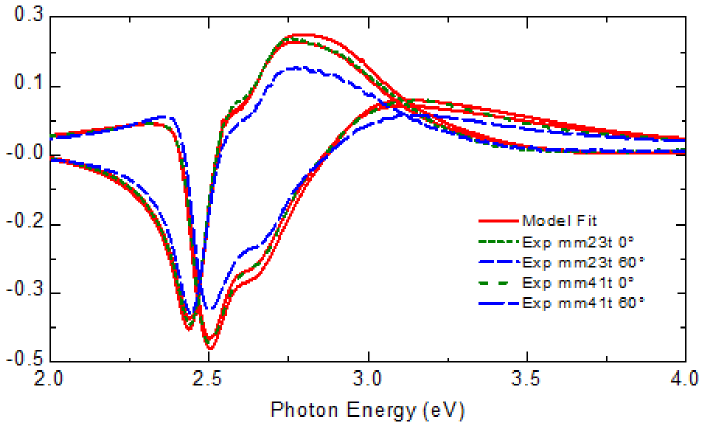

(b) Fit to M41 and M23 using uniaxial alpha

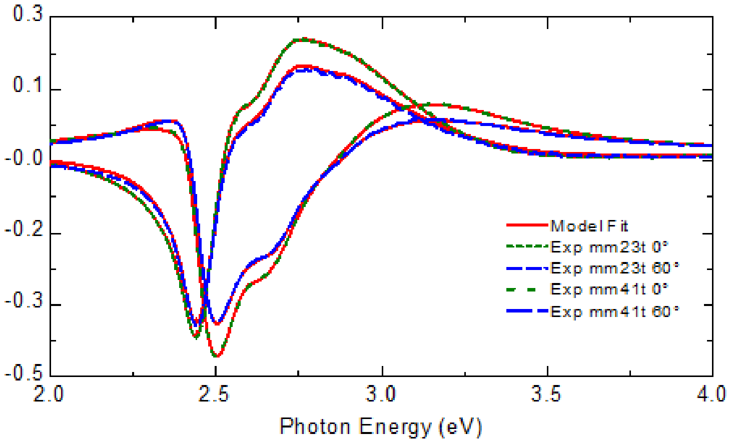

Supplementary Figure 10 MMSE data recorded in transmission and reflection at three different sample orientations (0, 45 and 90°) for annealed and aligned films of cPFBT.
Transmission MMSE: The film now exhibits circular effects and linear effects, the latter which move around within the MM elements upon sample rotation.

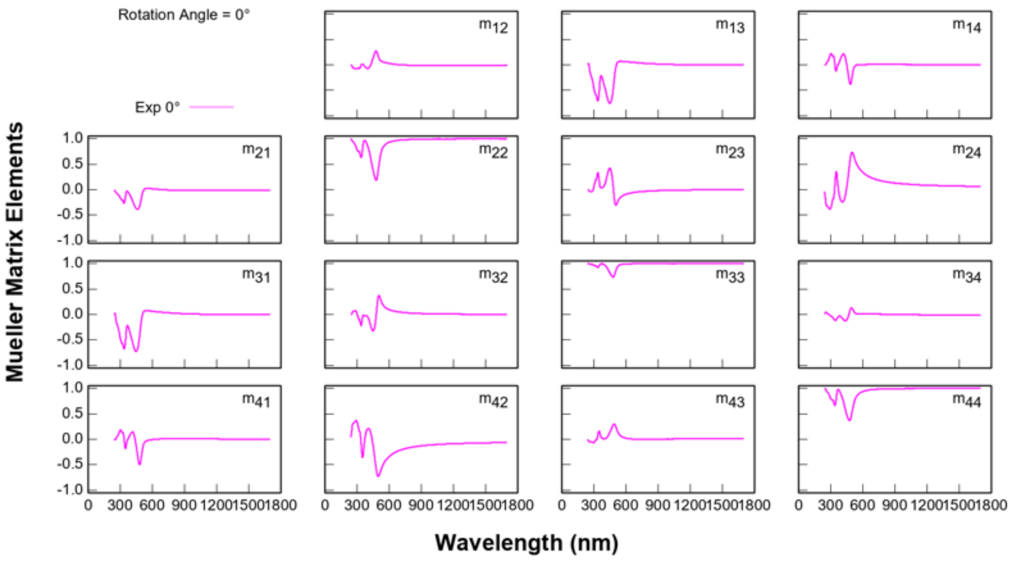

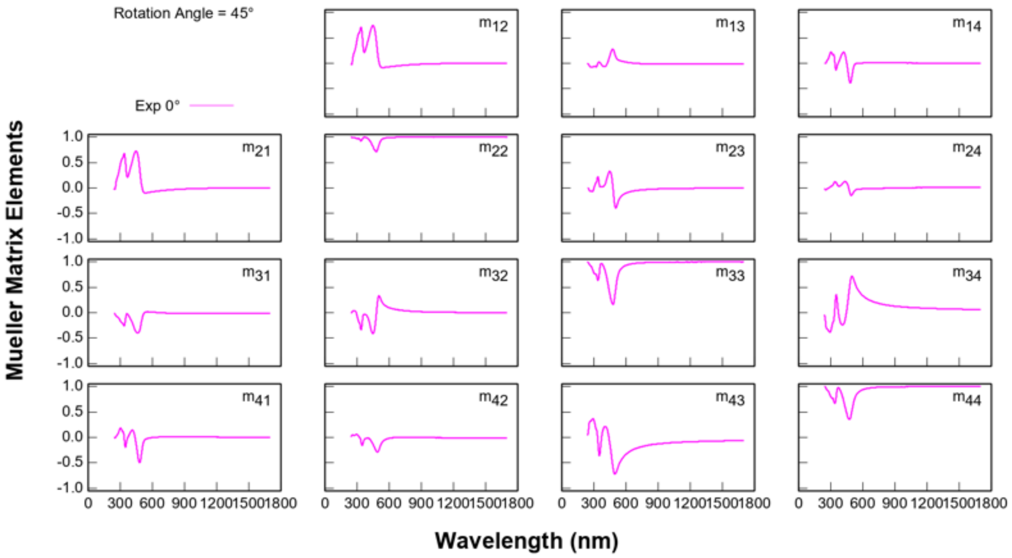

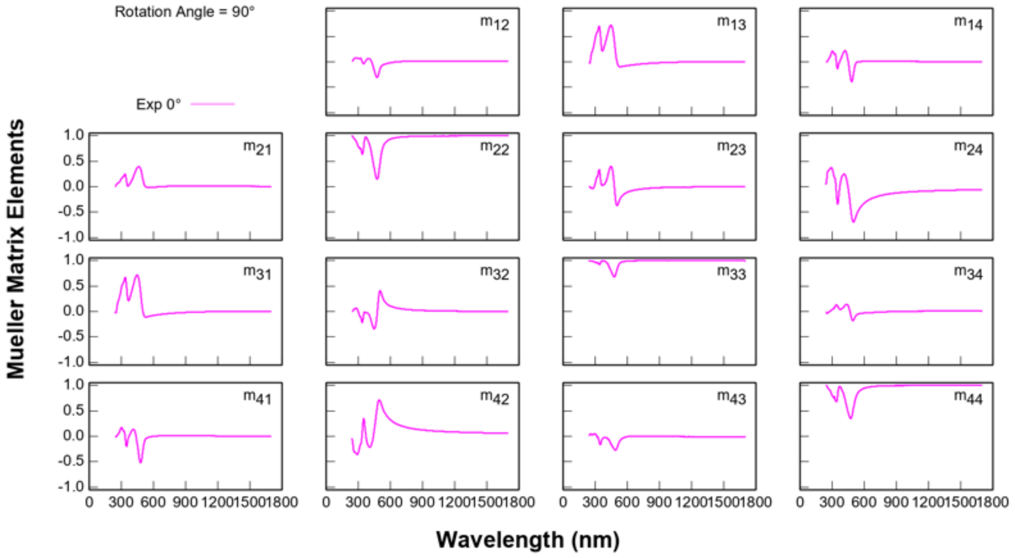

1

Reflection MMSE: There are clear circular and linear effects in the MM, where the combination in reflection rotates with the orientation of the sample.

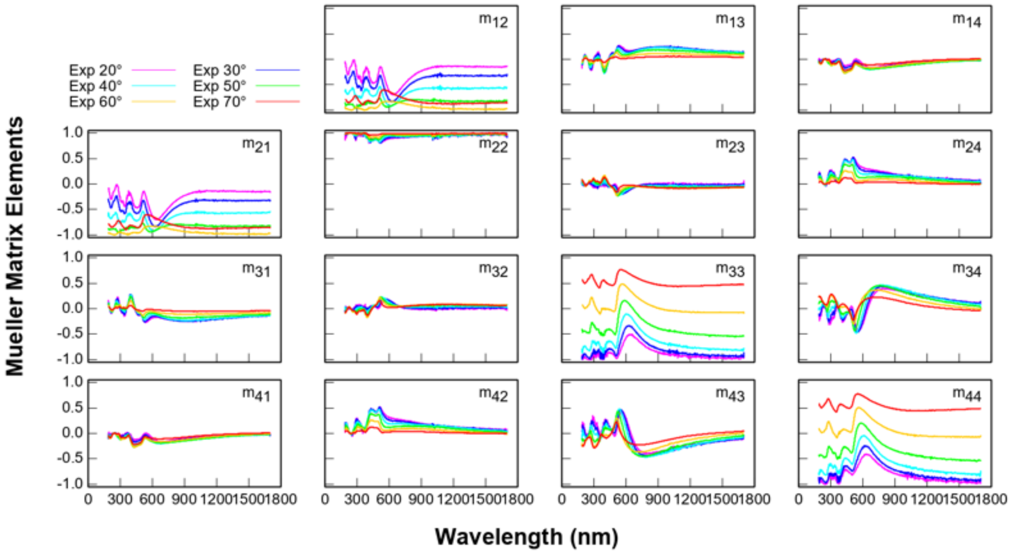

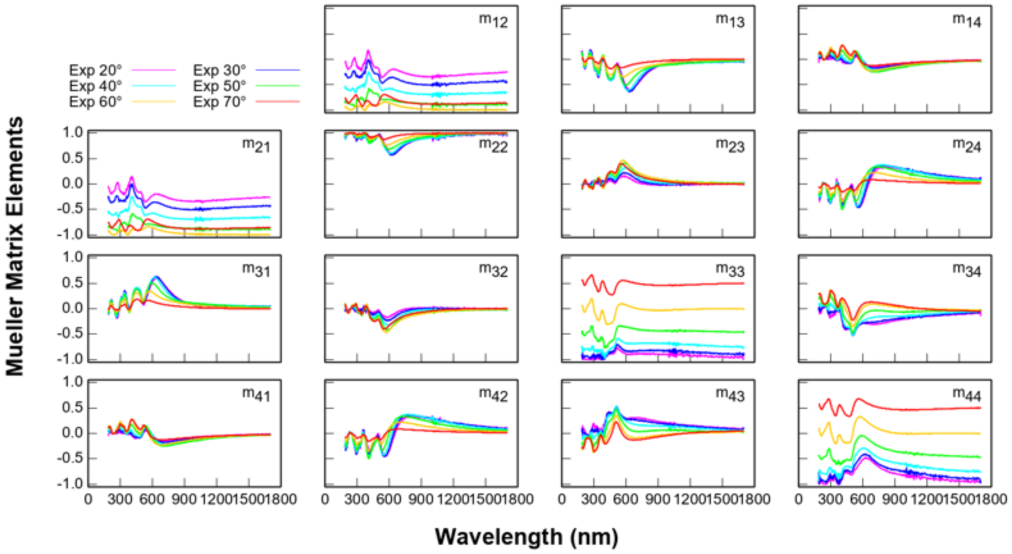

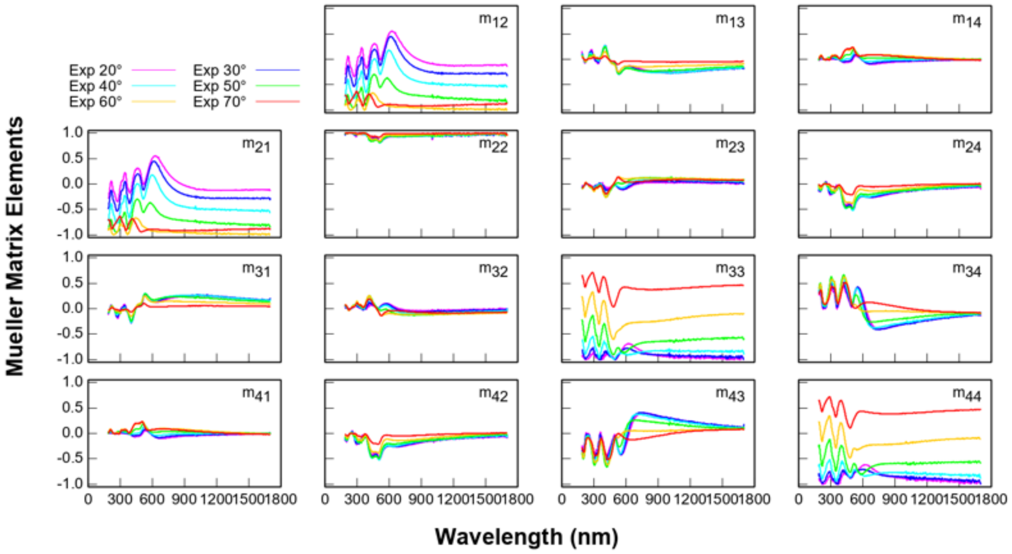

Supplementary Figure 11 Attempting to fit the reflected MMSE data recorded from aligned cPFBT films using an optical model with only uniaxial anisotropy where the optical axis is tilted into the sample plane, but is not allowed to twist.

Reflection, linear uniaxial model

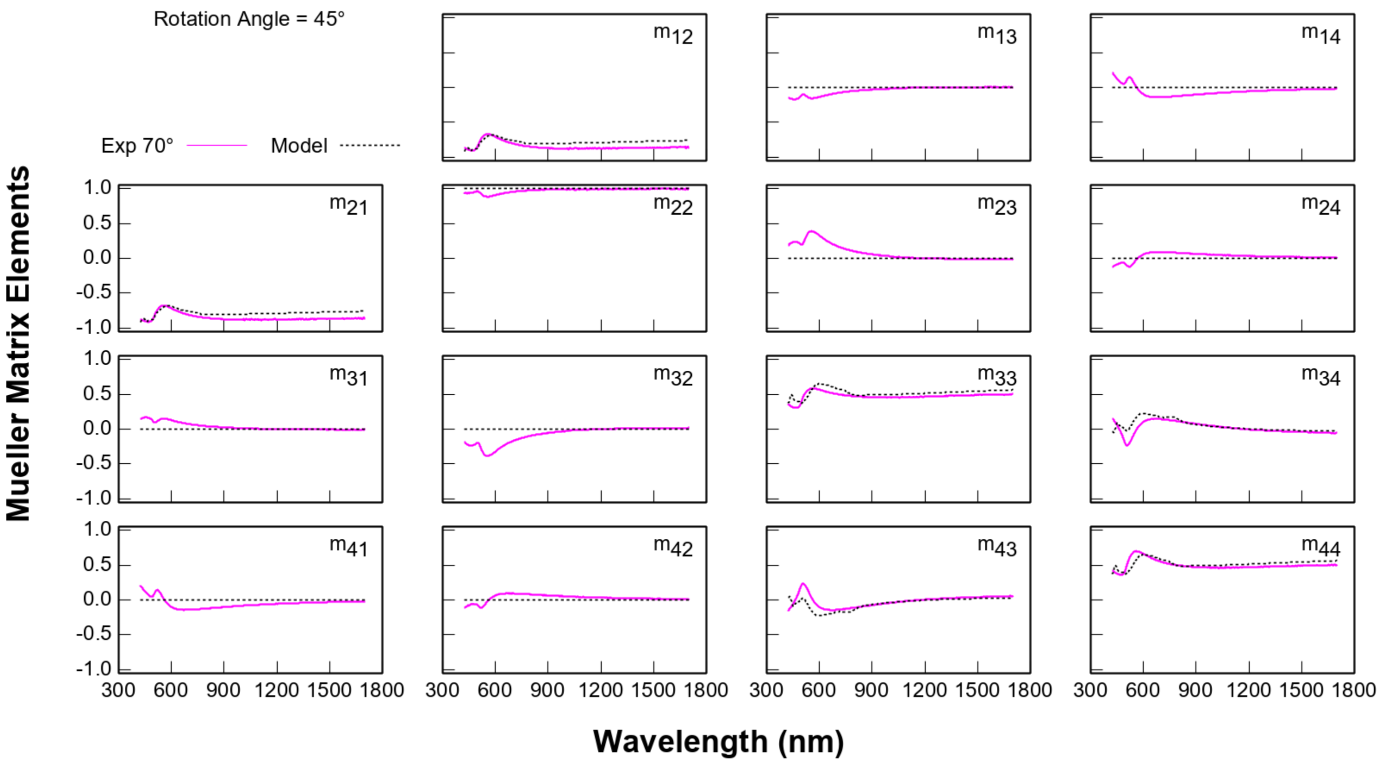

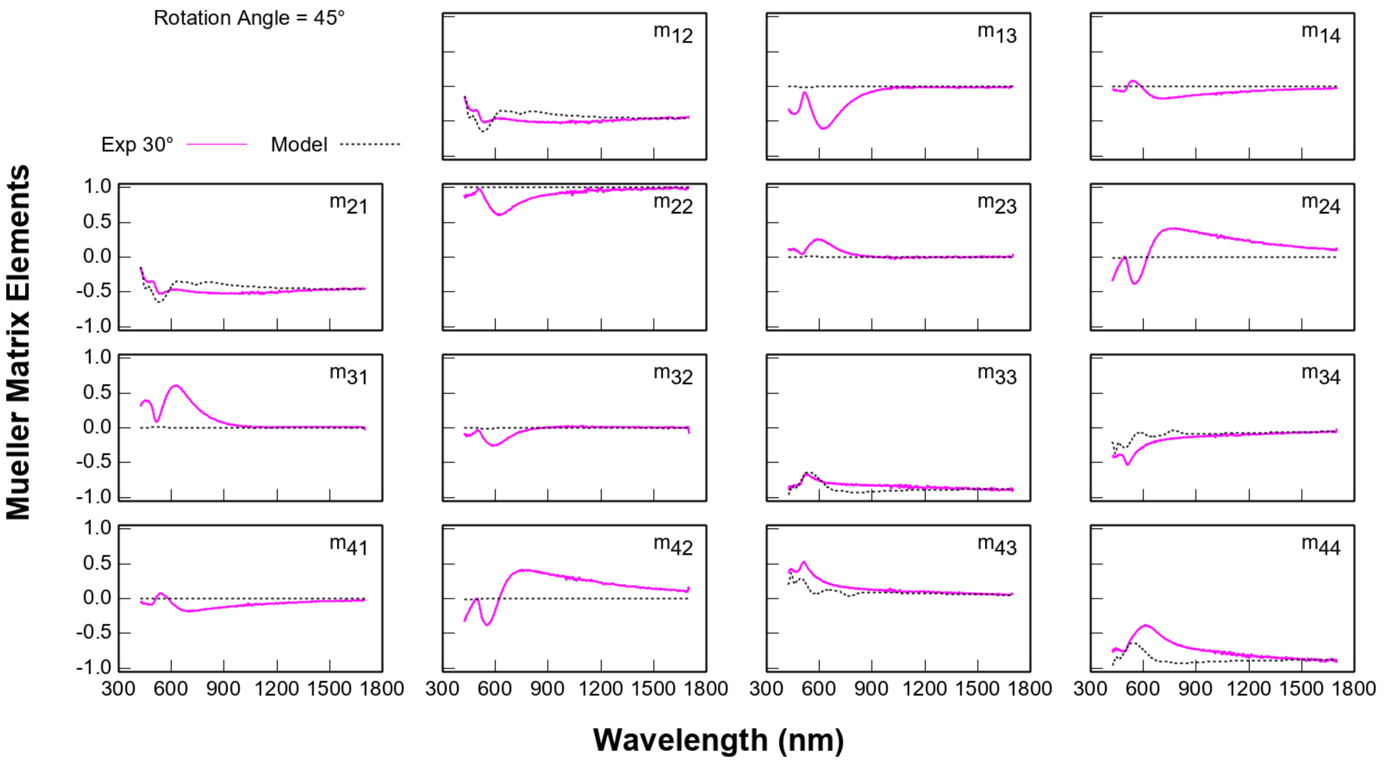

Transmission, linear uniaxial model

Attempting to fit the transmission MMSE data recorded from aligned cPFBT films using an optical model with only uniaxial anisotropy where the optical axis is tilted within the sample plane, but is not allowed to twist.

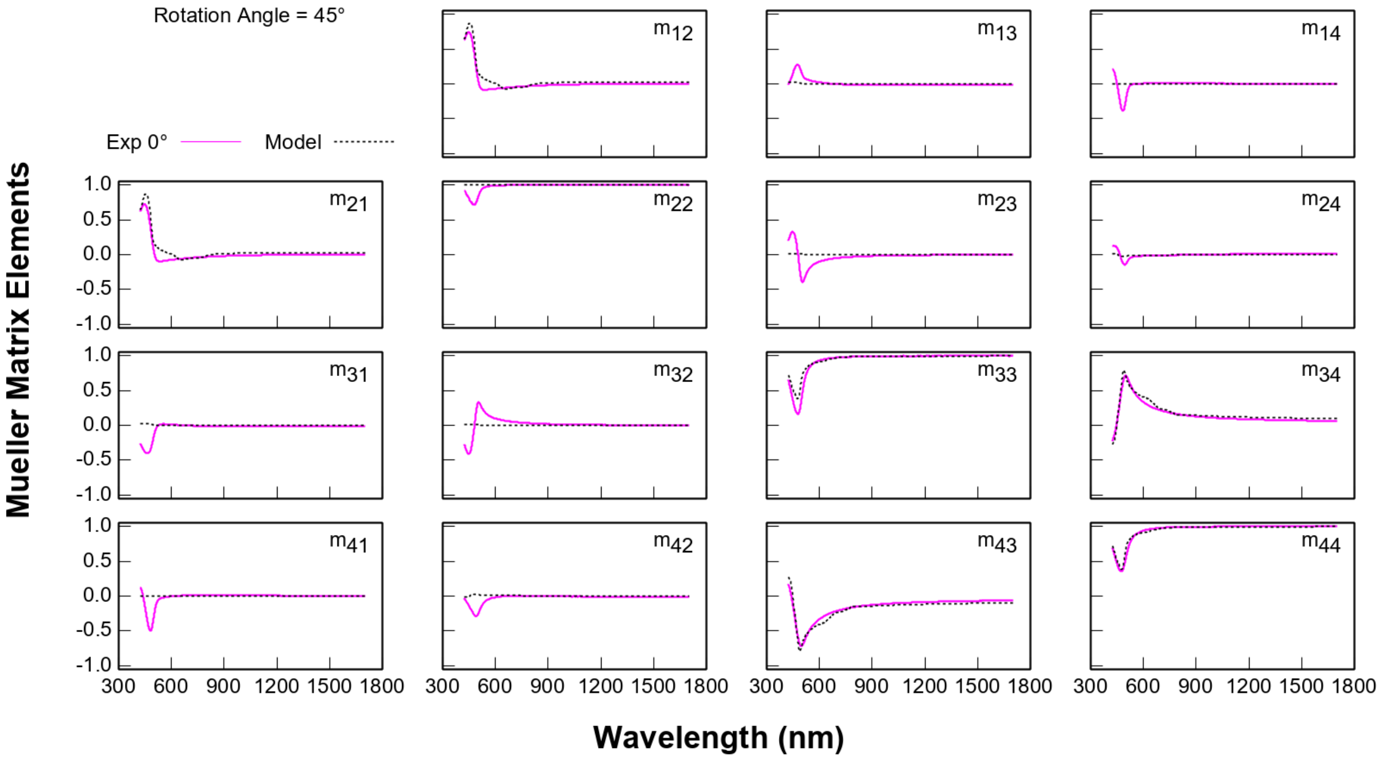

Reflection, linear uniaxial model as a function of sample rotation angle (incident: 70°)

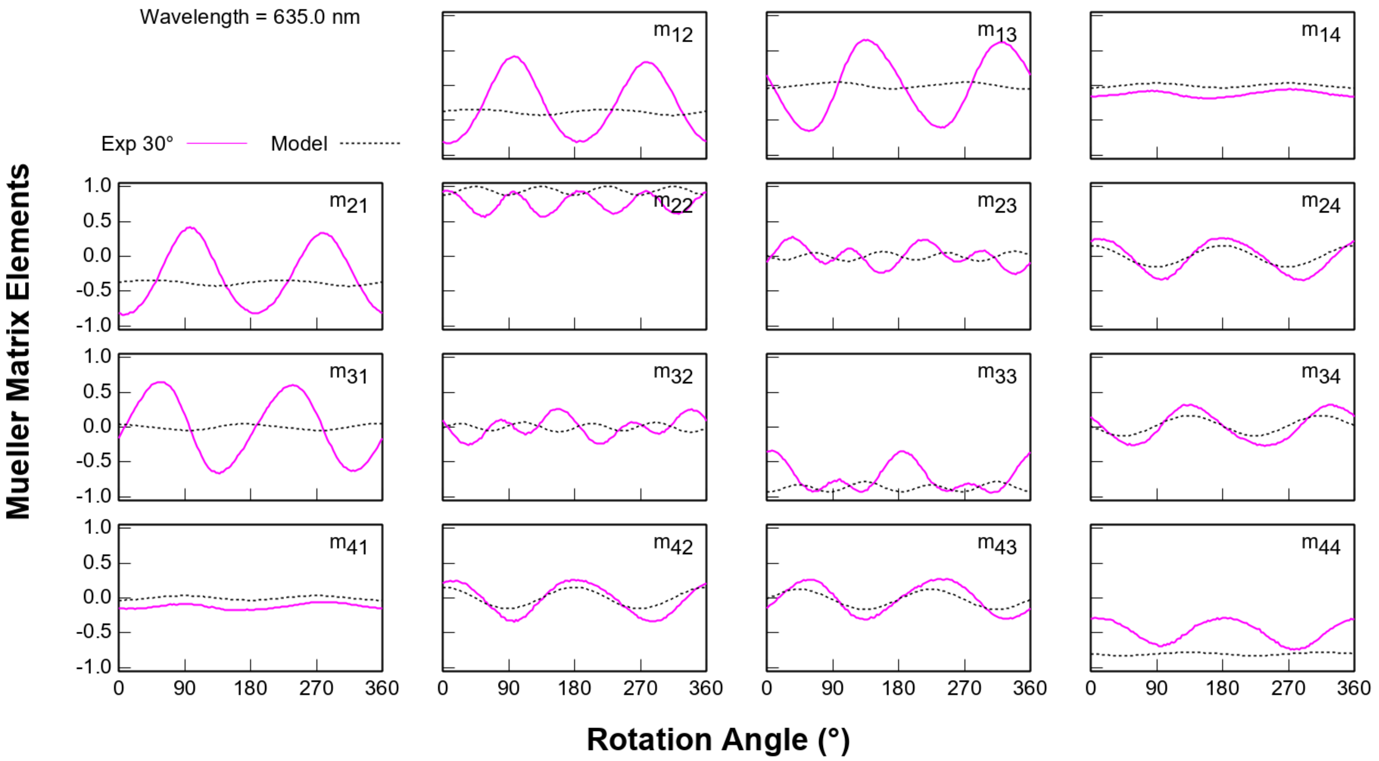

Reflection, linear uniaxial model as a function of sample rotation angle (incident: 30°)

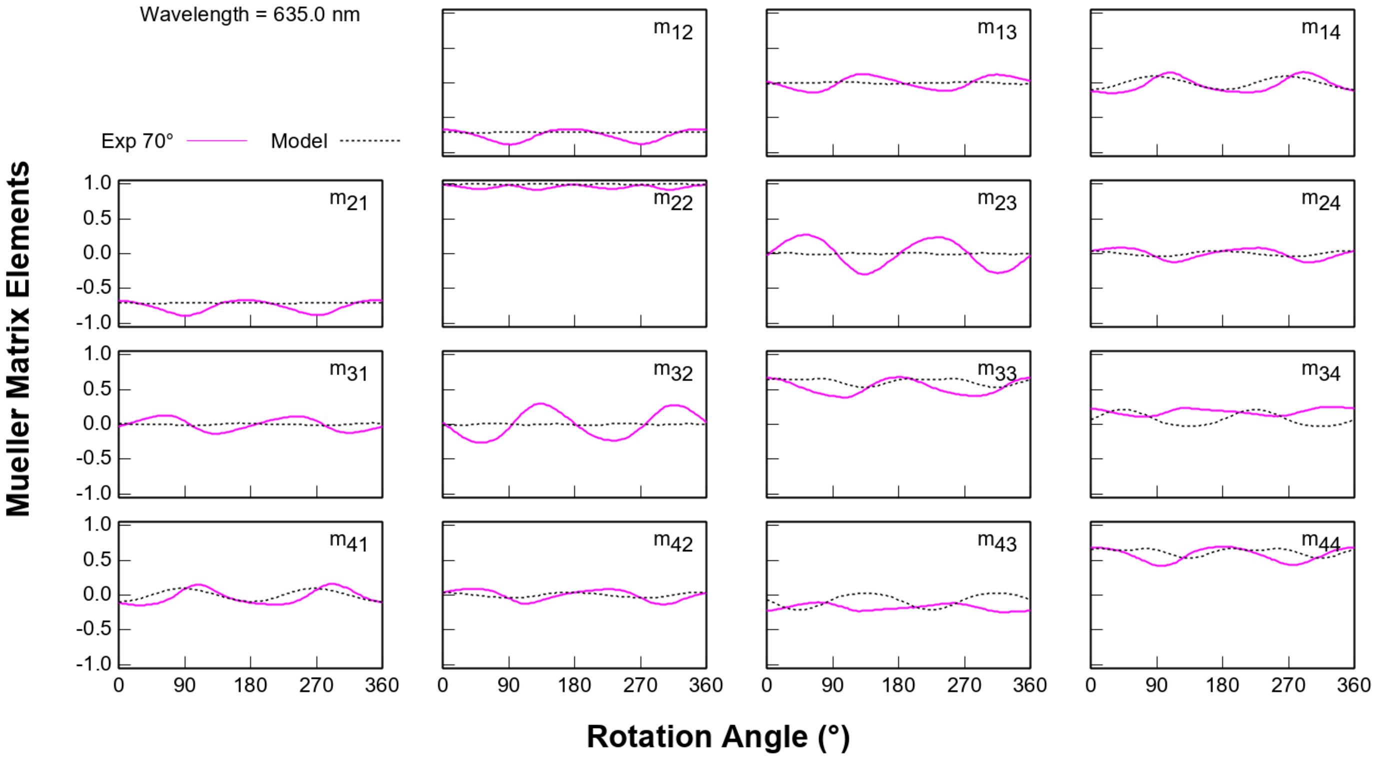

Transmission, linear uniaxial model, as a function of sample rotation angle

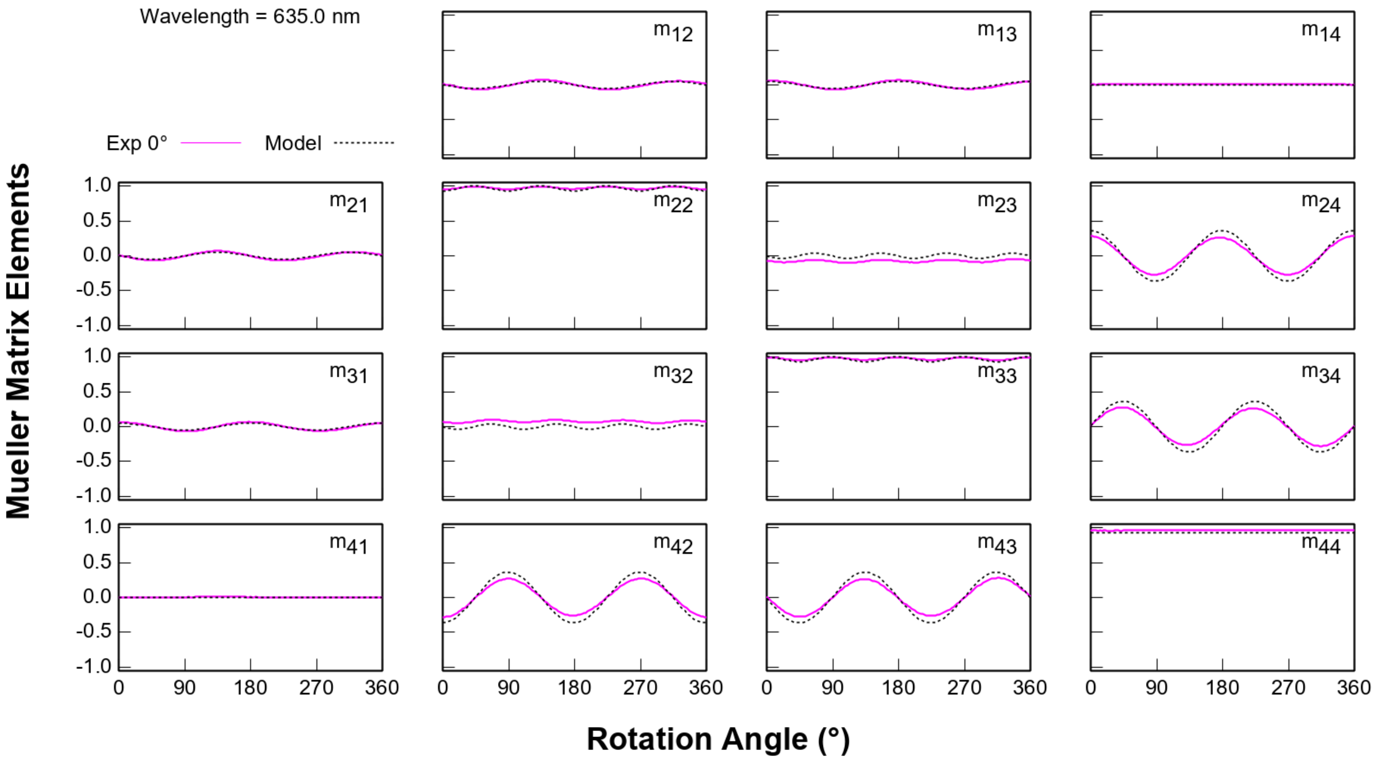

Supplementary Figure 12 Proposed optical model for aligned CSCP thin films

Uniaxial Anisotropy (linear) with about 85° of Counter-Clockwise twist.

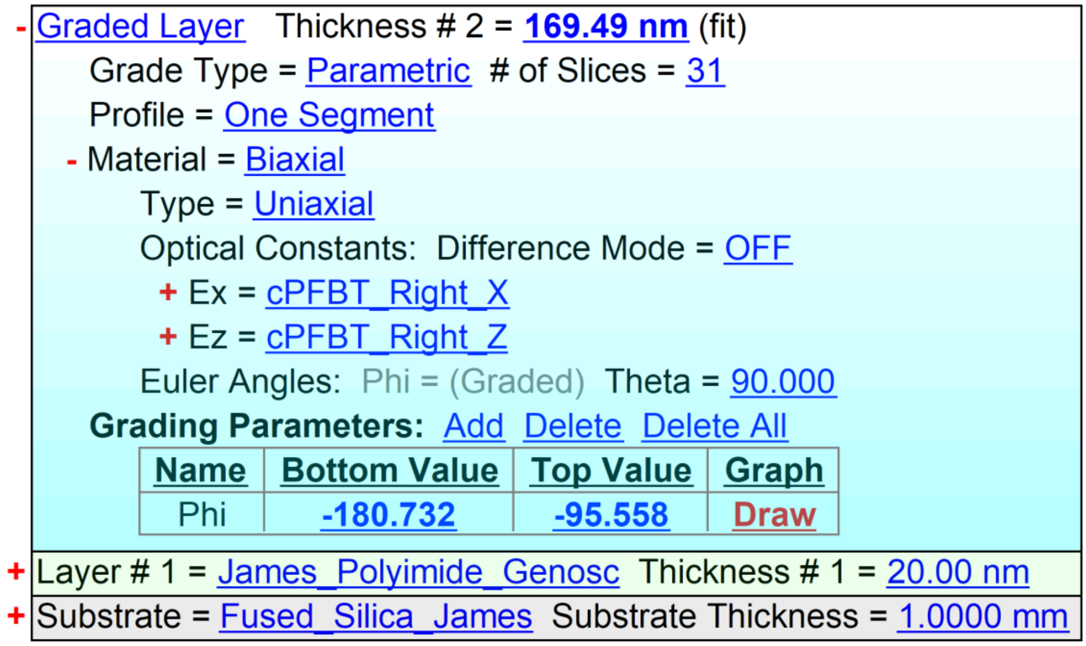

Model fits to the transmitted MMSE data recorded at three different sample orientations from aligned cPFBT films using an optical model that makes use of structural chirality.

Transmitted MMSE

Model fits to the transmitted MMSE data recorded at three different sample orientations (0, 45 and 90°) from aligned cPFBT films using an optical model that makes use of structural chirality.

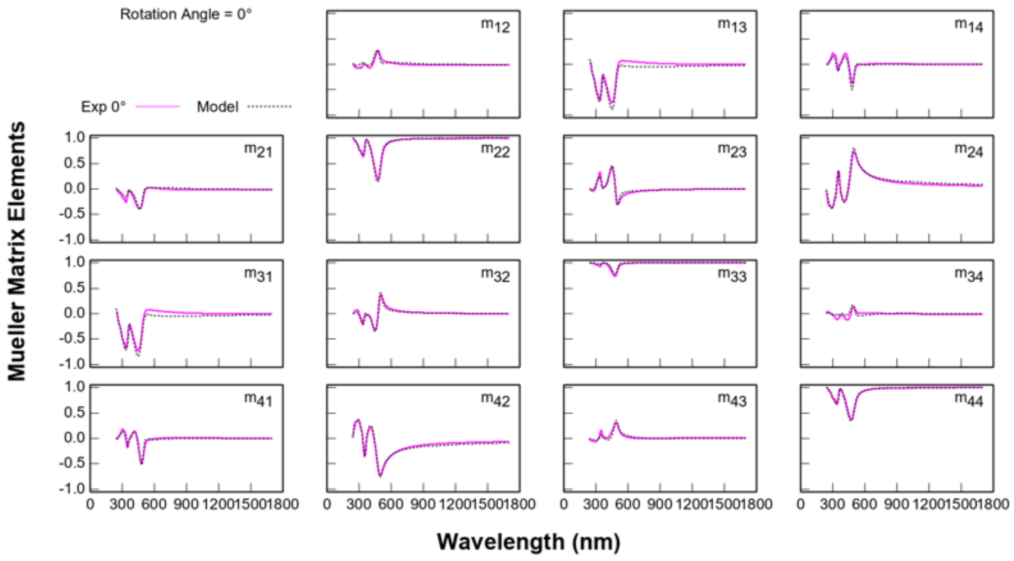

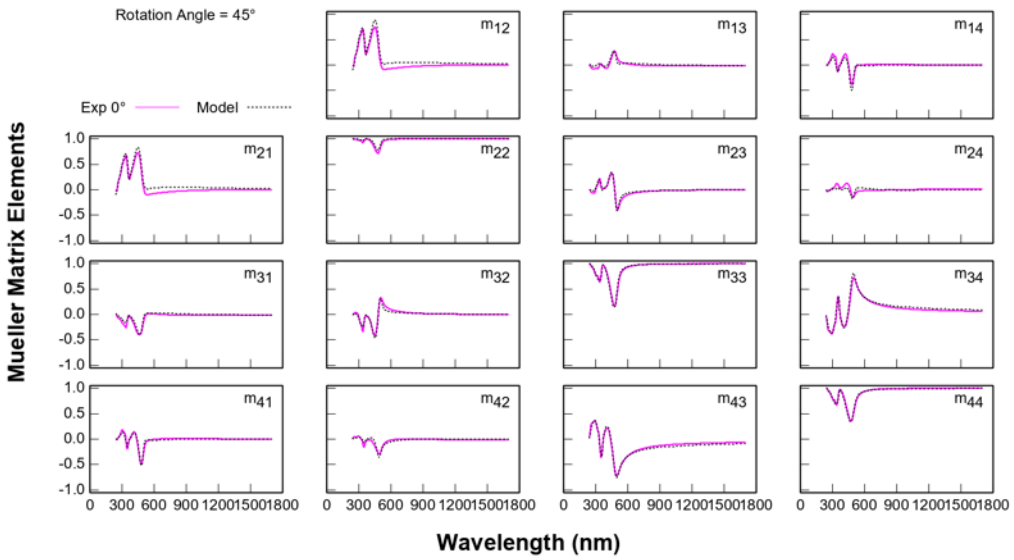

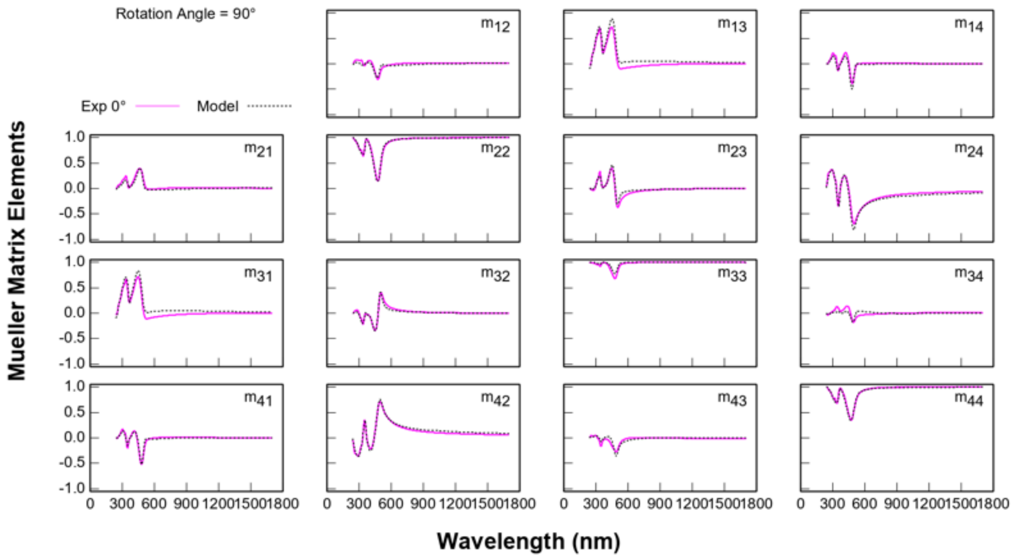

Reflected MMSE

Model fits to the reflected MMSE data at 30° angle recorded from 3 different sample orientations (0, 45 and 90°) of the aligned cPFBT films using an optical model that makes use of structural chirality.

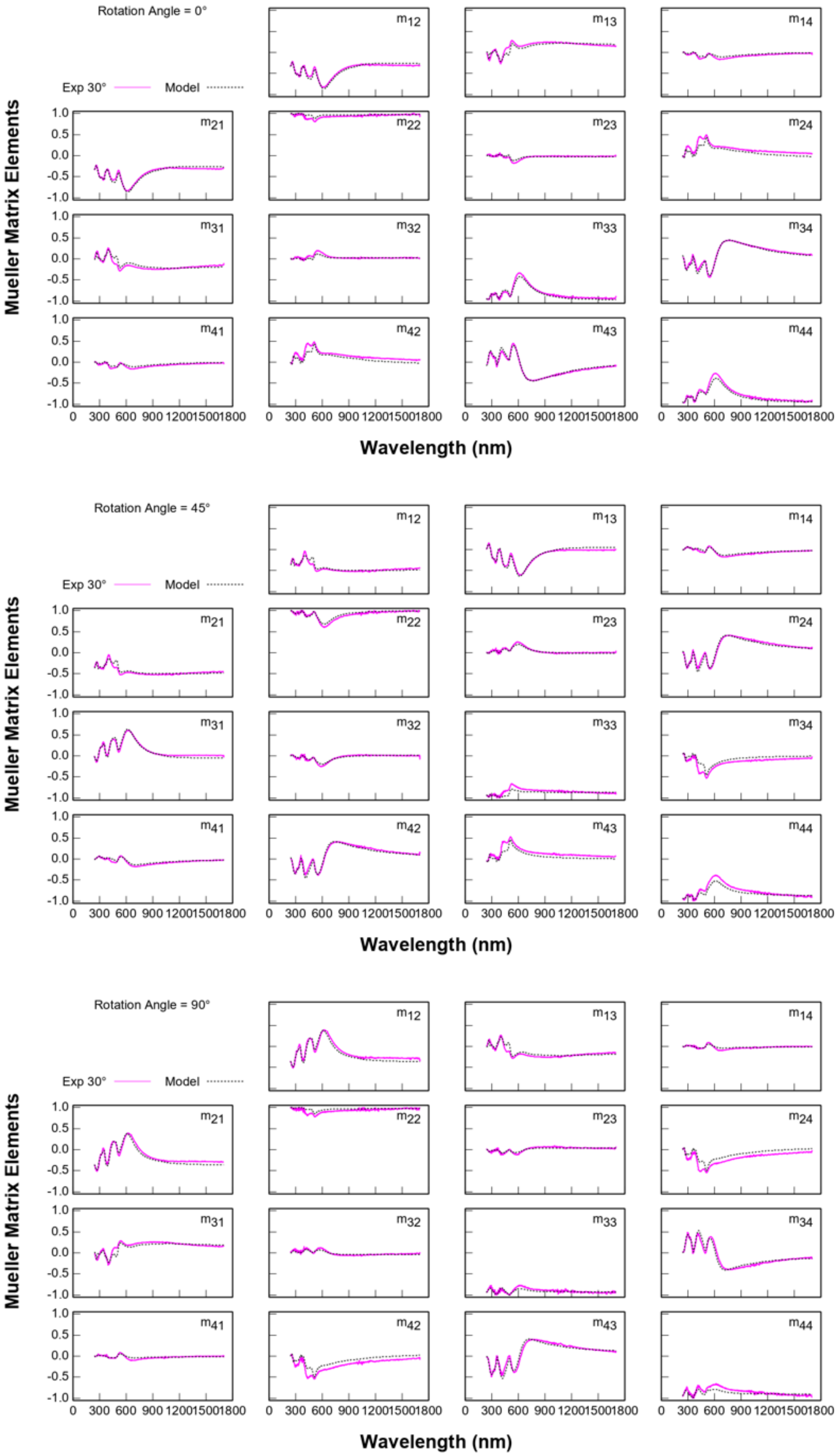

Fits to the reflected MMSE data recorded at 70° angle from three different orientations (0, 45 and 90°) of the aligned cPFBT films using an optical model that makes use of structural chirality.

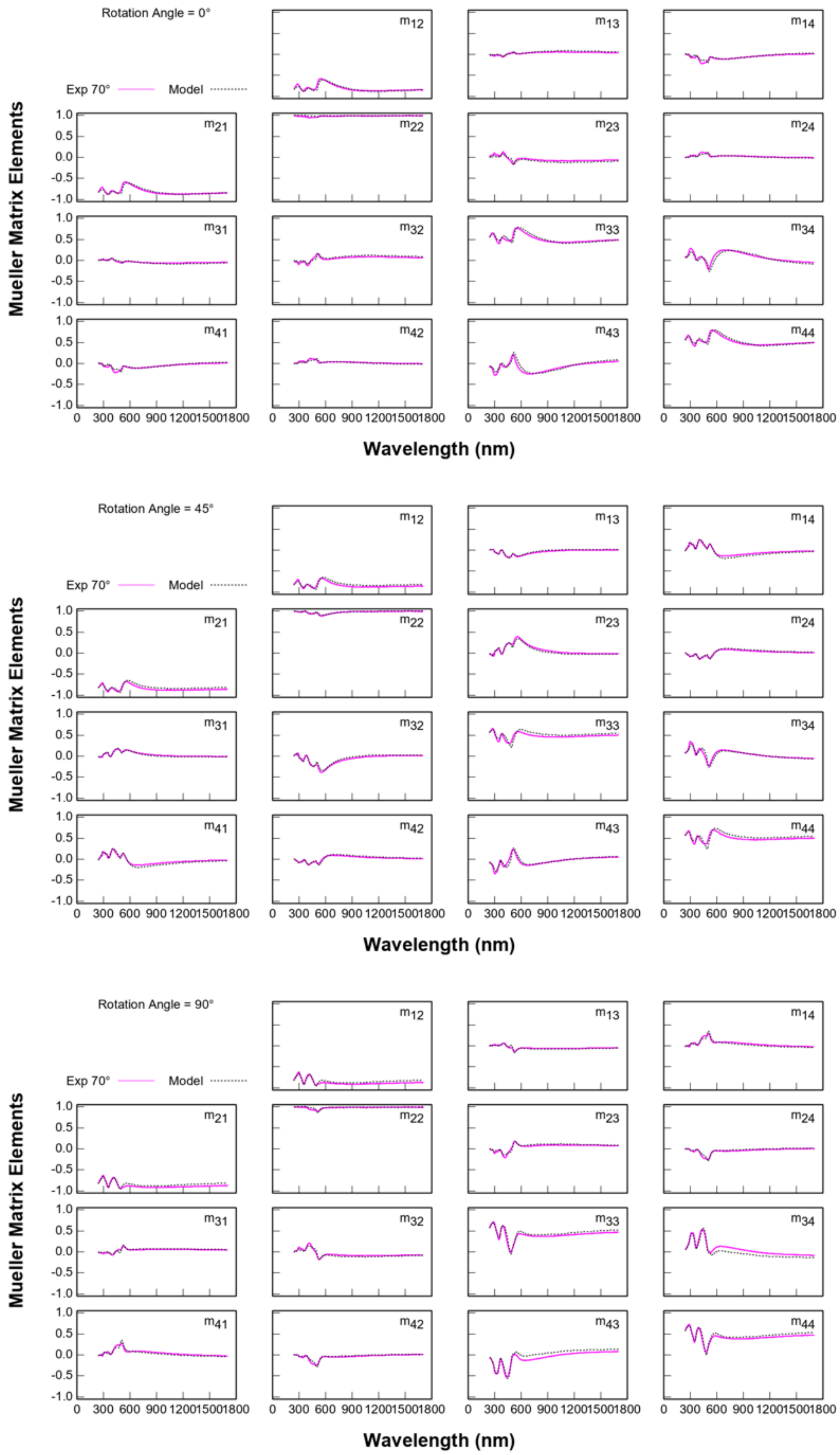

The fit utilizing structural chirality is also able to reproduce the reflected MMSE data recorded at 30° angle of incidence from aligned cPFBT films as a function of sample rotation.

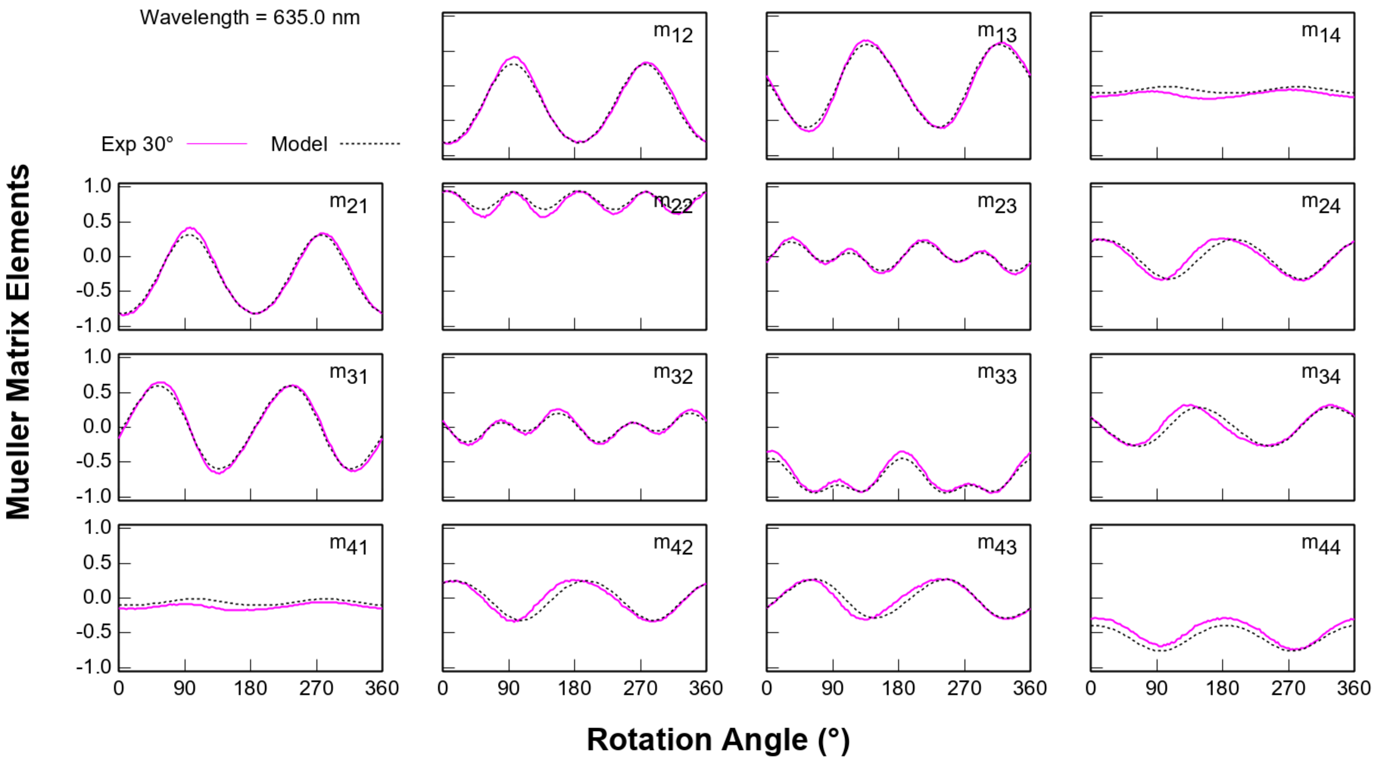

The fit utilizing structural chirality is also able to reproduce the reflected MMSE data recorded at 70° angle of incidence from aligned cPFBT films as a function of sample rotation.

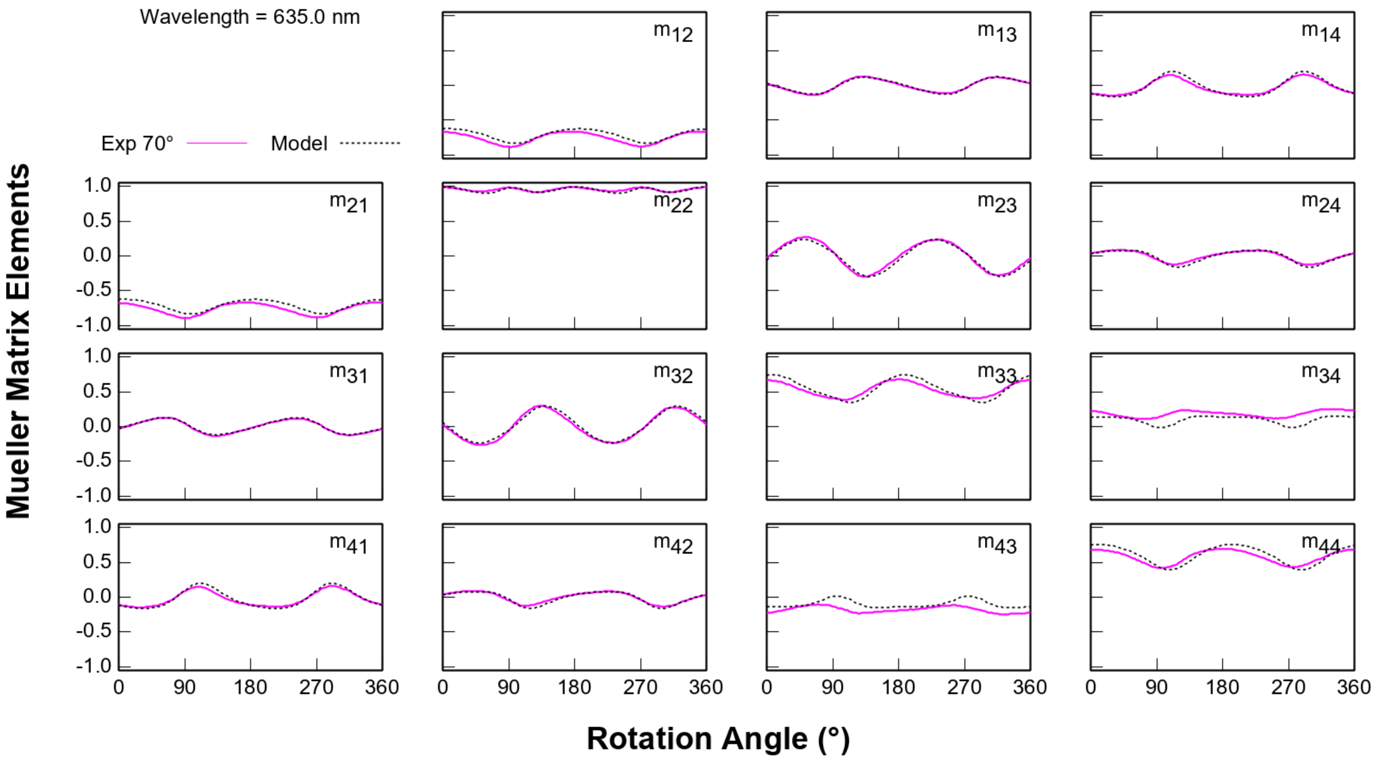

Transmitted MMSE: The fit utilizing structural chirality is mostly able to reproduce the transmitted MMSE data recorded at normal incidence (0°) from aligned cPFBT films as a function of sample rotation.

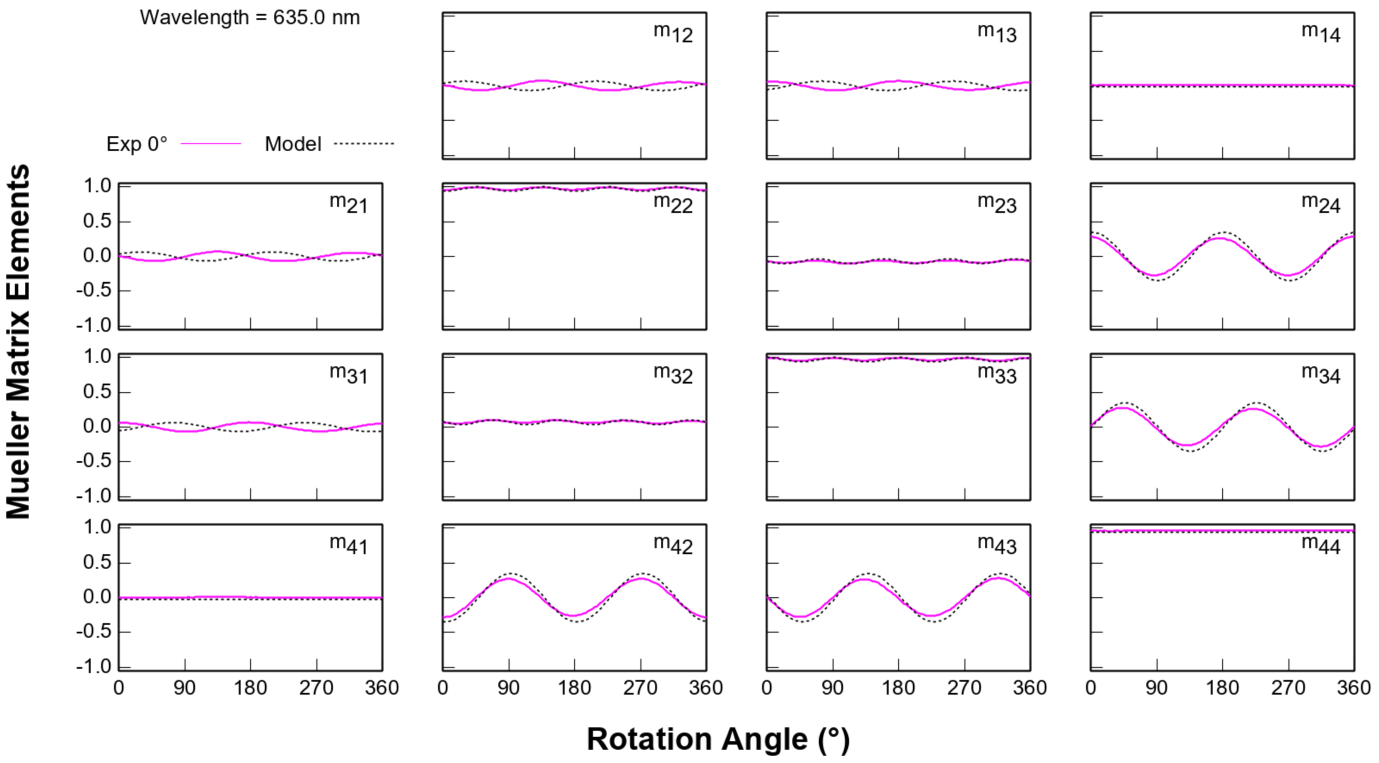

Full model

The final fit utilizing biaxial anisotropy and structural chirality was matched to MMSE data in both transmission and reflection versus angle of incidence and at three different sample orientations.

Reflection MMSE (sample orientation = 0 °)

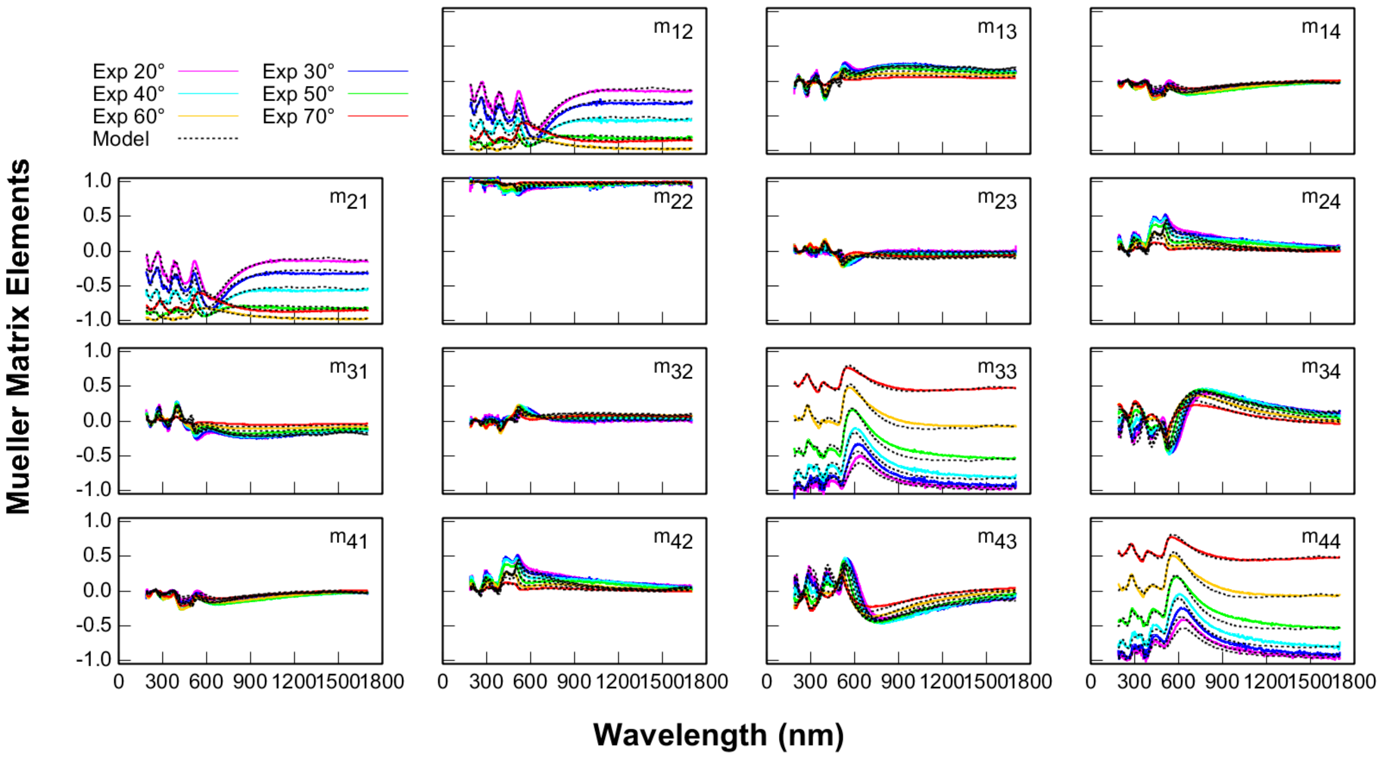

Transmission MMSE (sample orientation = 0 °)

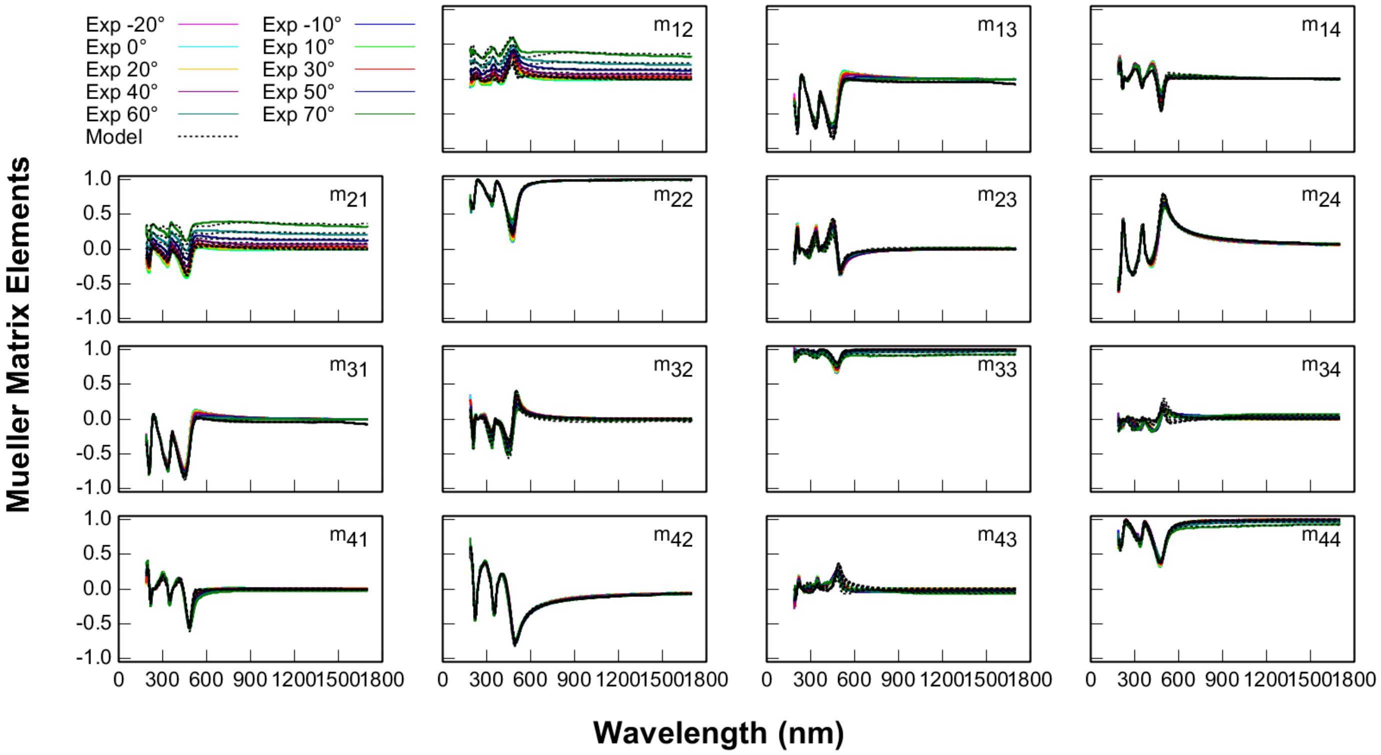

Reflection MMSE(sample orientation = 45 °)

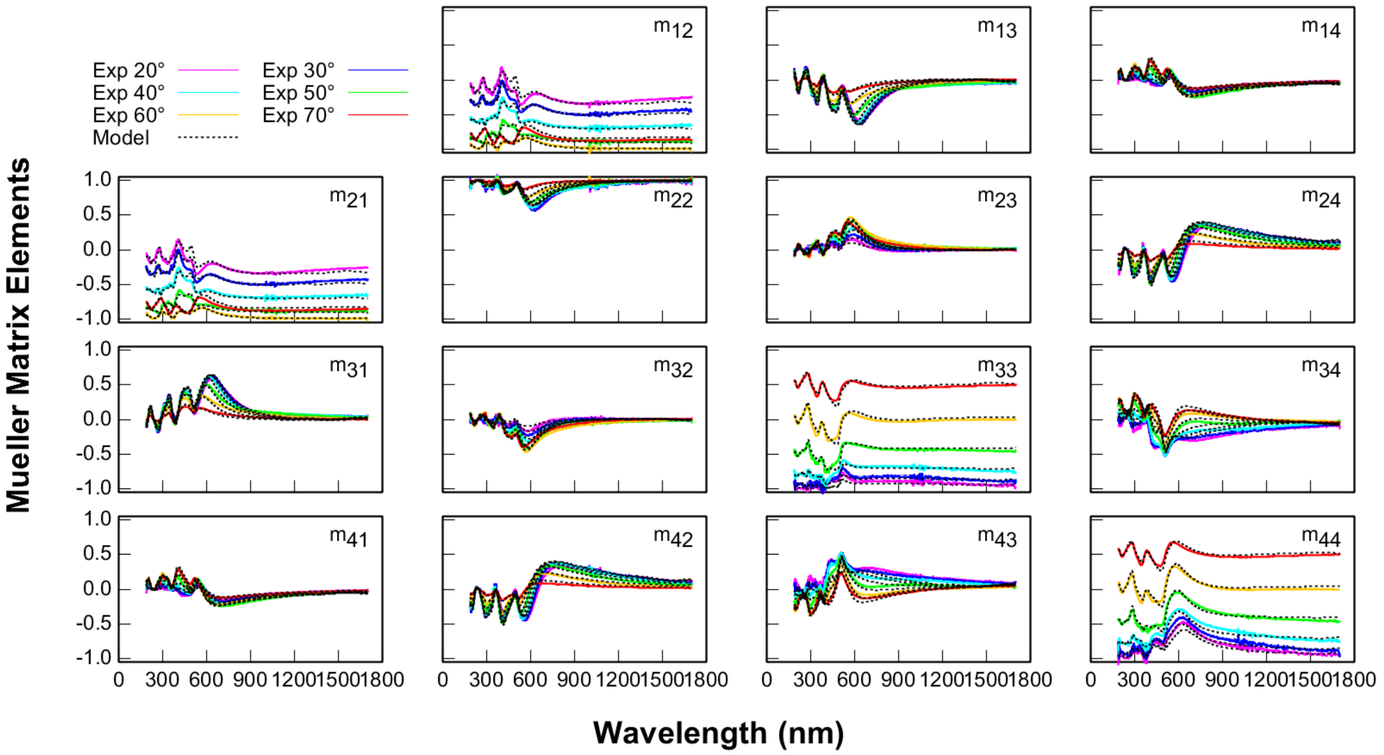

Transmission MMSE (sample orientation = 45 °)

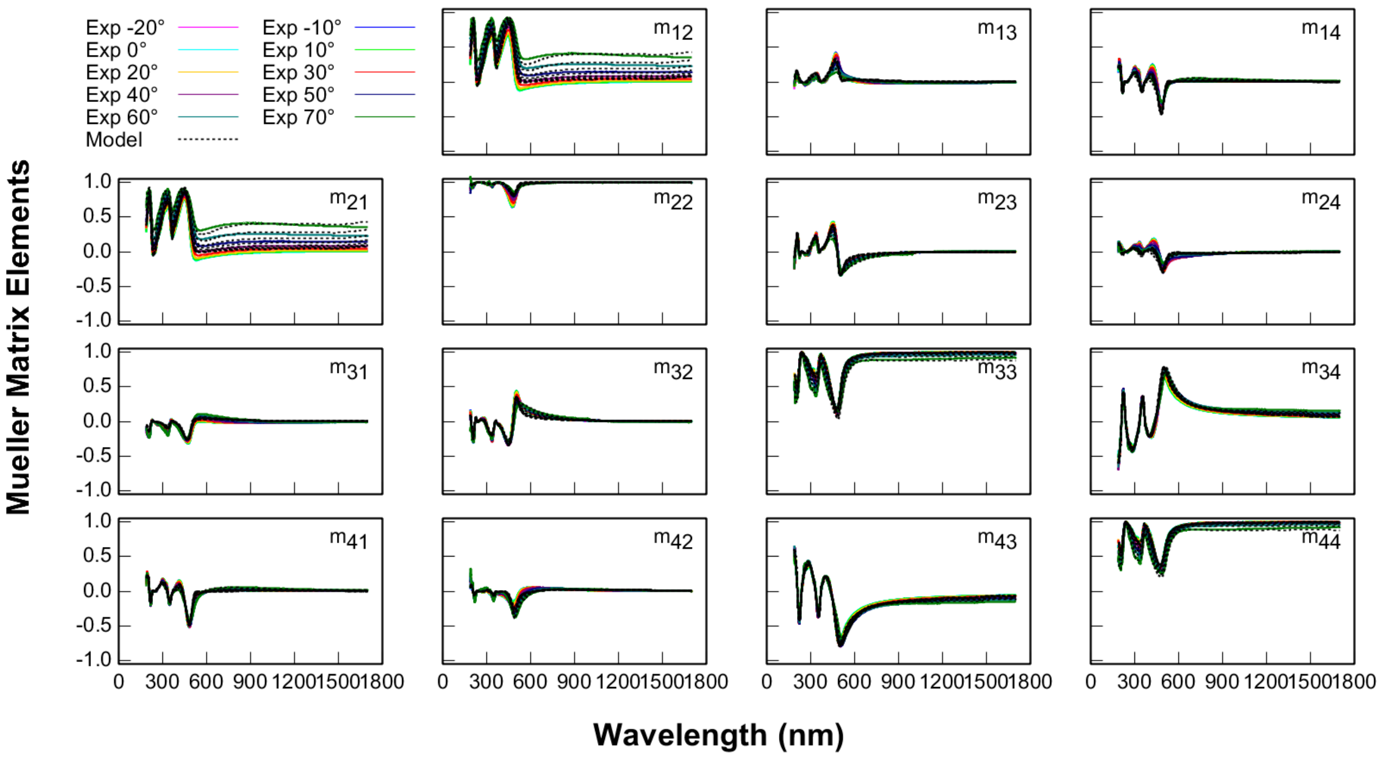

Reflection MMSE (sample orientation = 90 °)

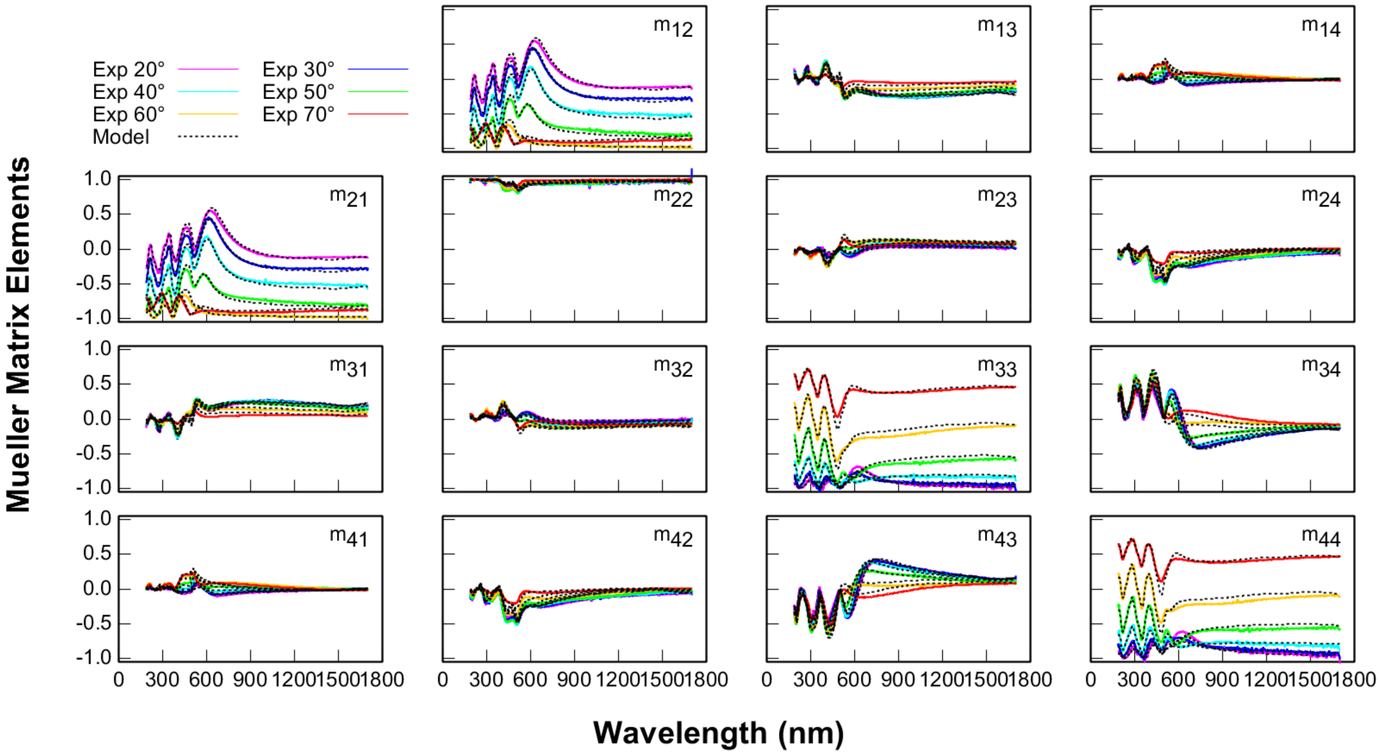

Transmission MMSE (sample orientation = 90 °)

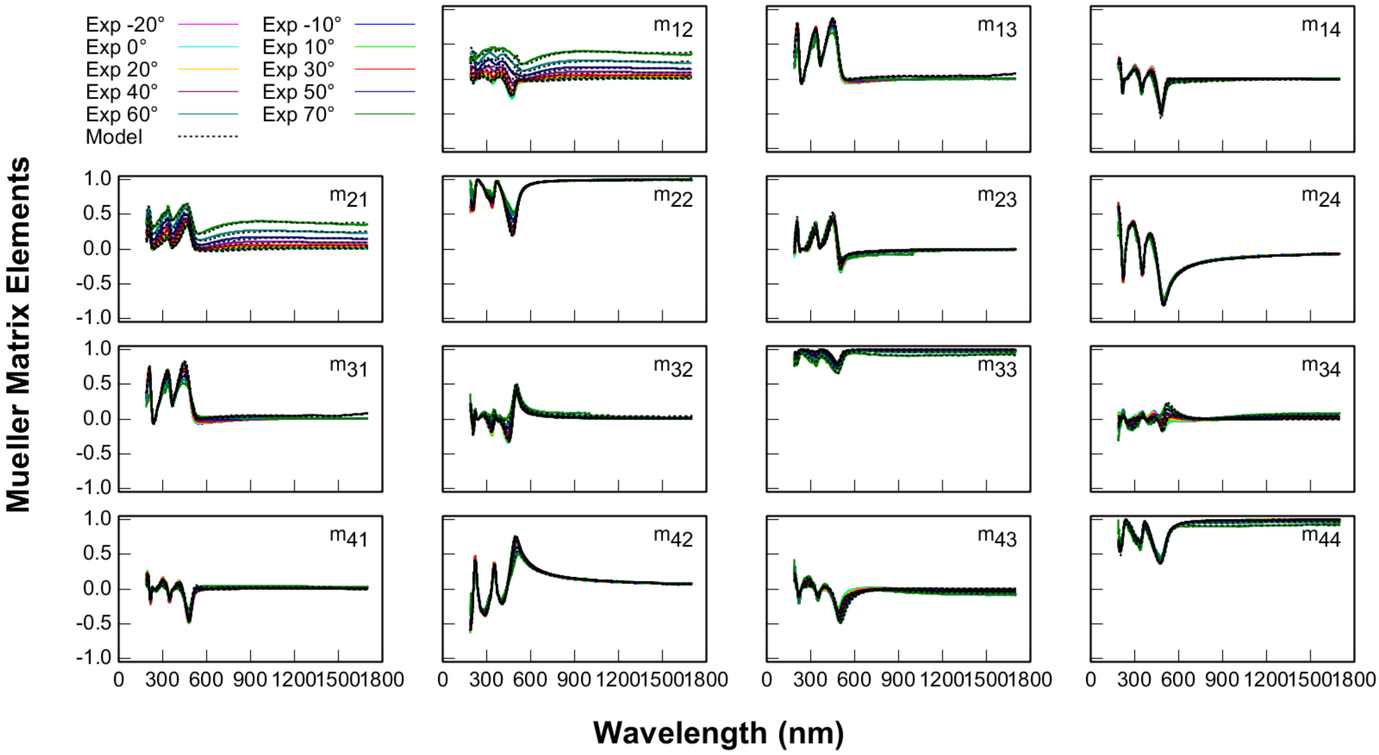

The uniaxial anisotropy from the final fit is shown here.

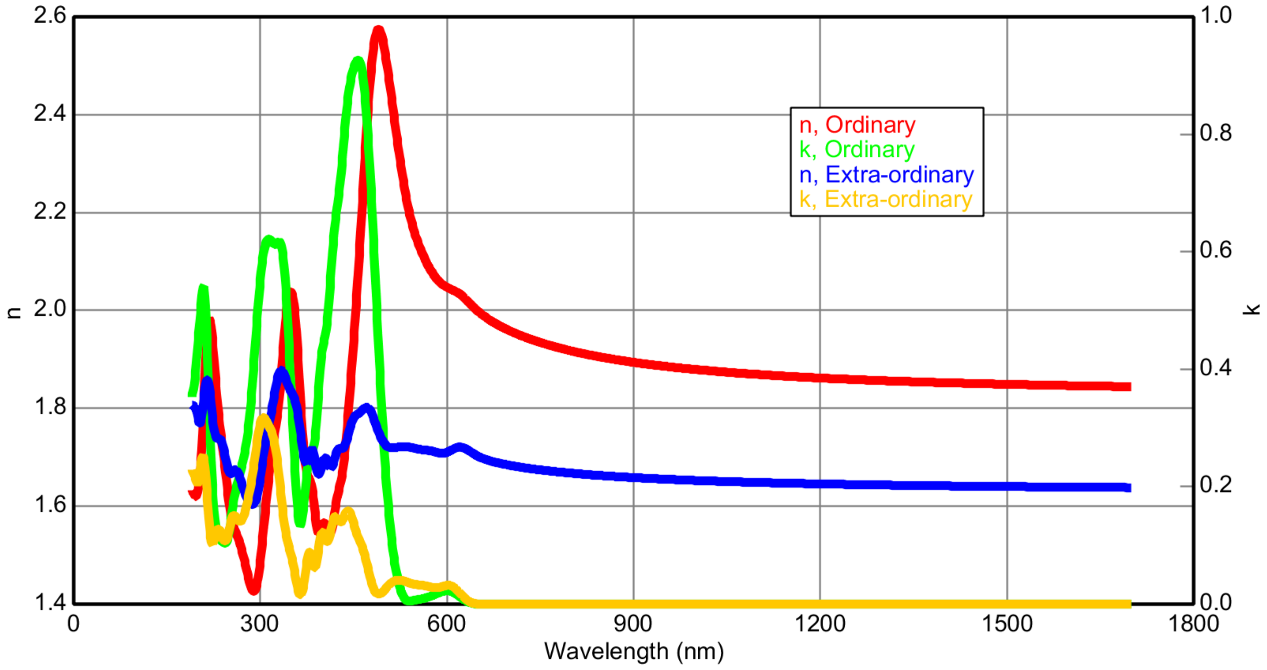

Supplementary Figure 13 Mueller matrix data recorded in transmission and reflection for annealed aligned films of F8BT:aza[M]H.

Transmission MMSE:

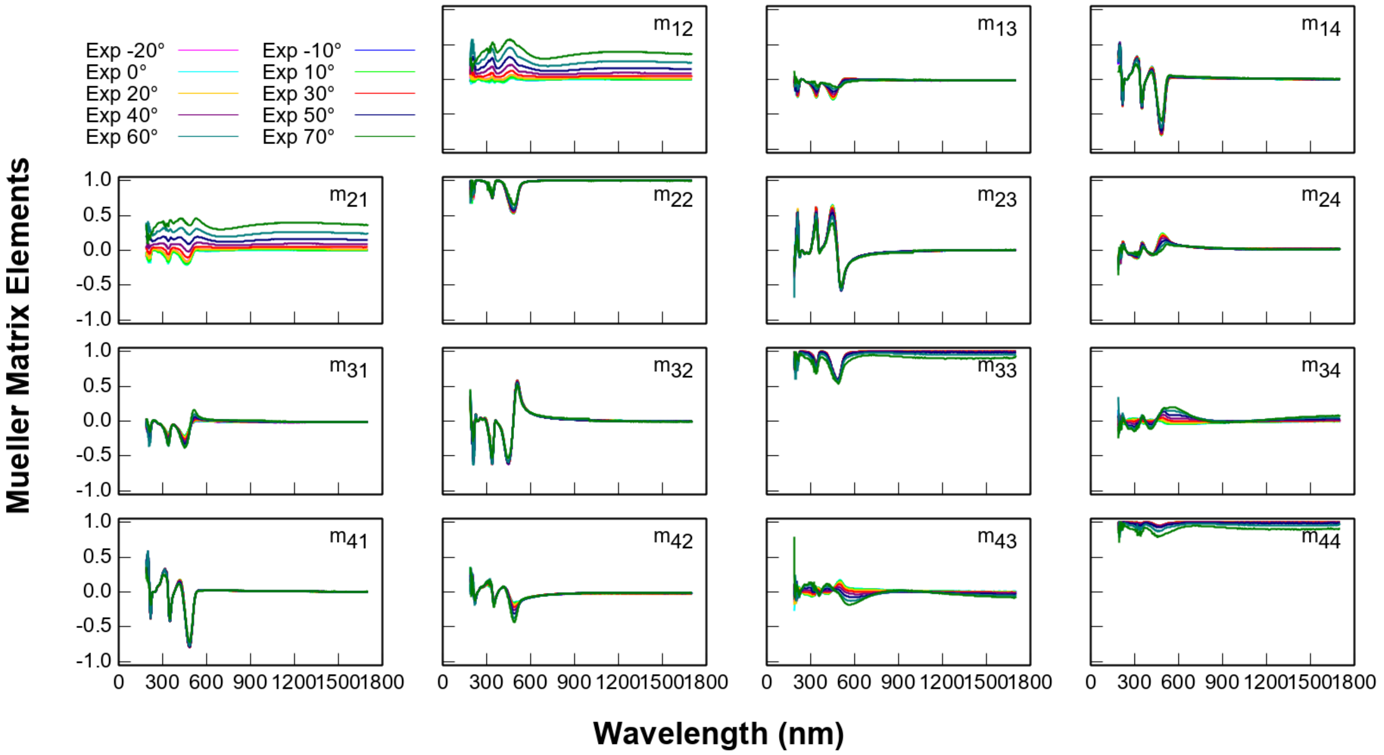

Reflection MMSE:

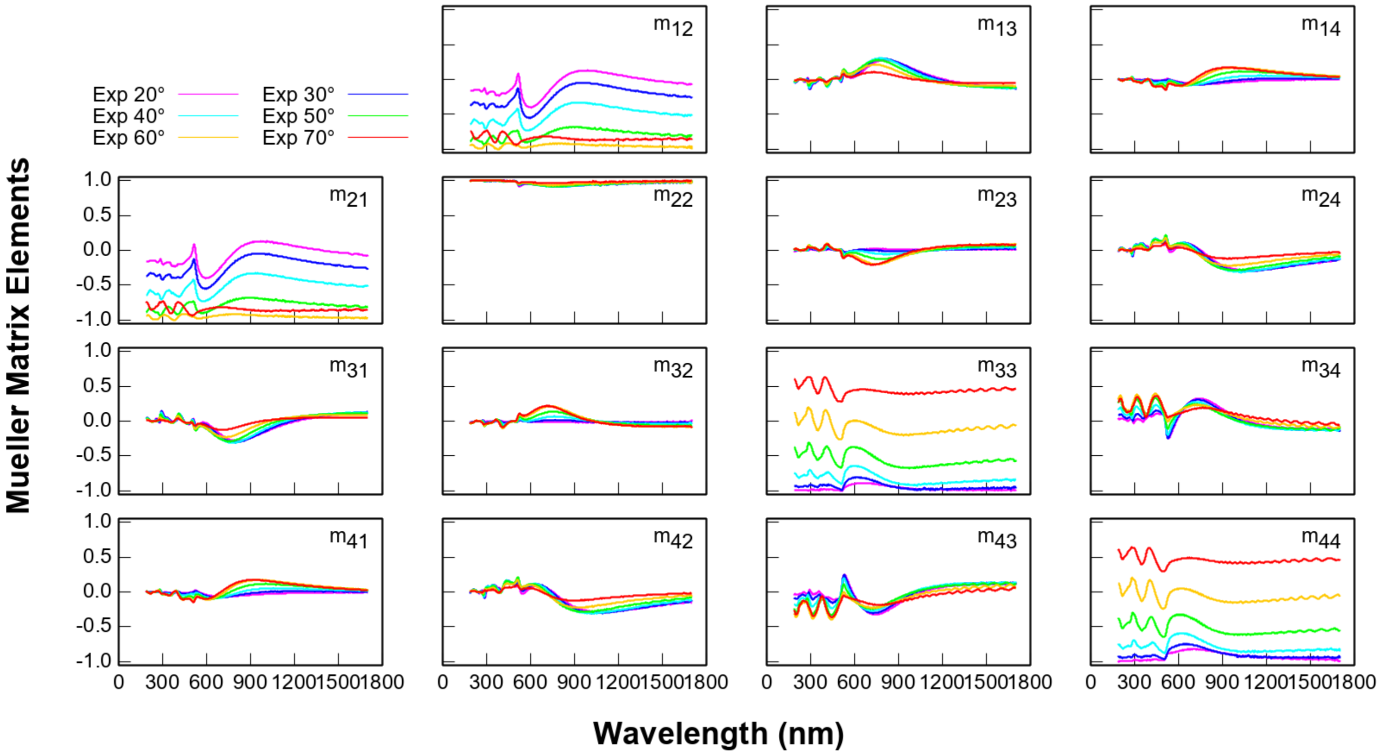

A twisting model was not adequate and strongly suggests the combination of both twisting phenomena and natural optical activity.

Supplementary Figure 14 RSoXS for as-cast and annealed ACPCA thin films

RSoXS measurements are performed in a transmission geometry using a soft x-ray scattering beam line. The X-ray photon energy (E = 283.5 eV) was resonant with the carbon K-edge. The neat and as-cast ACPCA thin films ((a) – (d)) demonstrate features characteristic of uniaxial anisotropy but no detectable Bragg scattering. On the other hand, the scattering patterns in the annealed ACPCA thin films ((e), (f)) indicate that the polymer chain is arranged in a periodic structure, where characteristic length scales (x) can simply be extracted from the peak wave vector (q_H) for Bragg scattering.

$$q_H = \frac{2\pi}{x}$$

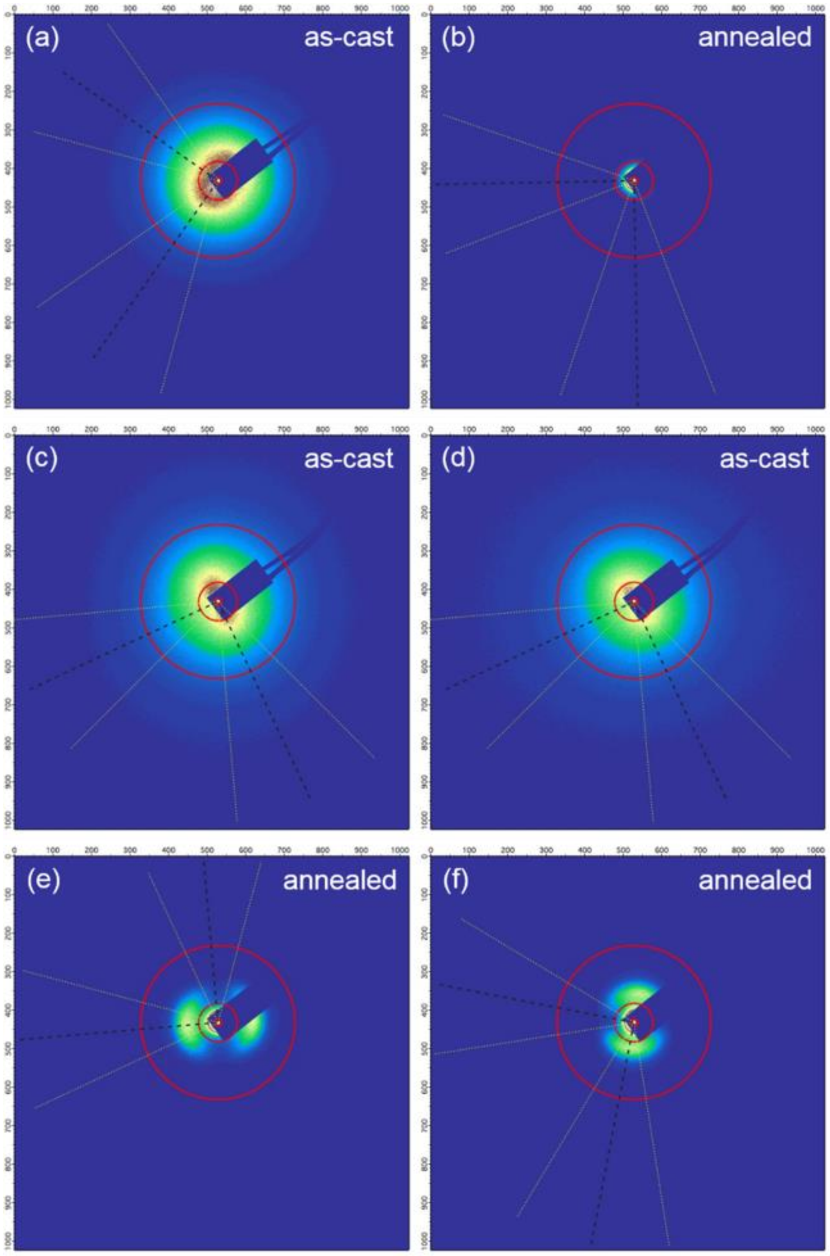

RSoXS patterns of the (a) as-cast and (b) annealed neat polymer thin films, and (c) and (d) as-cast and (e) and (f) annealed ACPCA (F8BT: aza[P]) thin films recorded for two perpendicular polarisations of the incident beam.

Supplementary Figure 15 Radial RSoXS scans

Radial scans of q about the beam centre q = 0 of the annealed neat achiral polymer (a) and ACPCA (b) thin films allow extraction of the peak wave vector (q_H). This corresponds to a periodic feature of 280 nm.

(a)

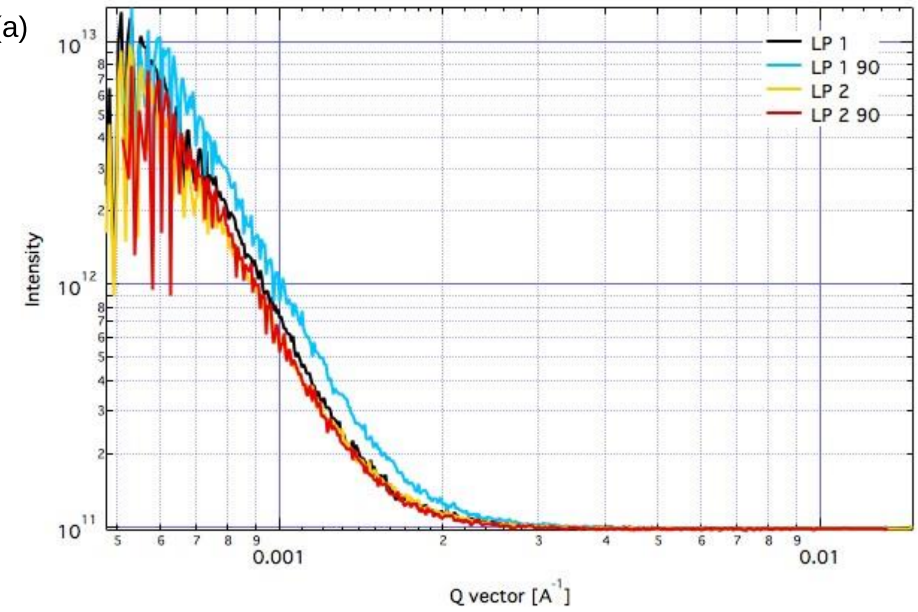

(b)

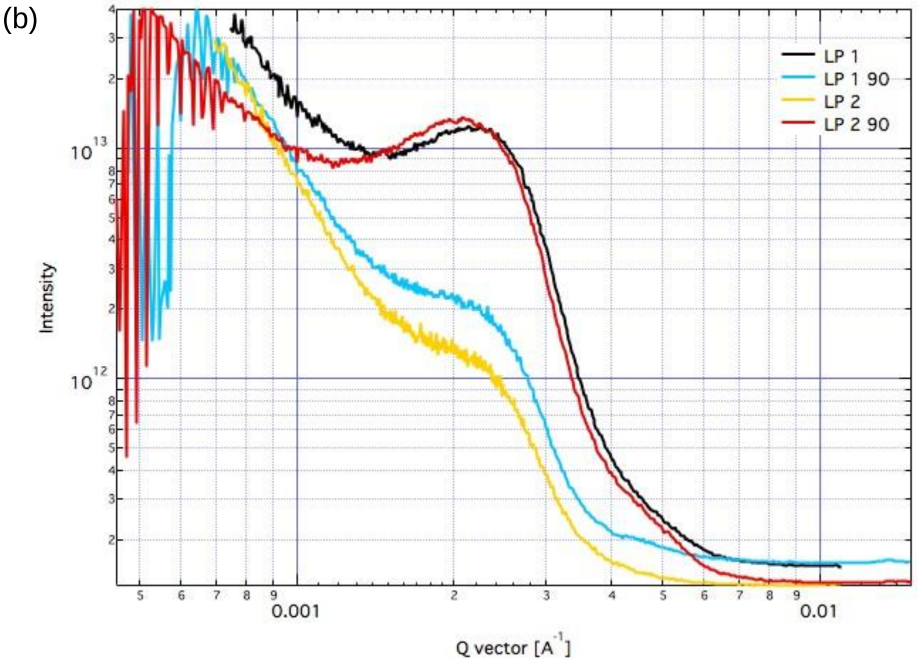

Supplementary Discussion 3 of AFM measurements

To probe the organisation at the air-film interface of the annealed thin films, tapping mode (AC mode) atomic force microscopy (AFM) was carried out under ambient conditions. The surface of the annealed and unaligned F8BT-based ACPCA film (Figure 5a) is very smooth, with a roughness (RMS) value of 0.49 nm (calculated over a 1.0 μm^2 scan area). In the absence of the chiral aza[6]H additive (Fig. 5b) the surface has a higher roughness, 0.81 nm RMS. The lower roughness of the ACPCA film is also evident in the height profiles (Figs. 5c and 5d).

A comparison between the topography images of the annealed pure F8BT polymer and the blend with the azahelicene (Figure 5a and 5b) reveals that a fibril-like organisation is particularly evident for the sample with the chiral additive, and, though present in the pure F8BT is less well resolved without the chiral additive. Overall, both surfaces are isotropic, in that there are no globally preferred directions for the fibrils, while very locally they are clearly aligned with a characteristic average separation of 25 nm. Assuming an inter-chain van der Waals interaction of 0.42 nm we can estimate the number of polymeric chains within a single fibril is around 60.[22]

A comparison of Figures 5a and 5b suggests that, although the fibrillar structures are present in both samples, the addition of the chiral additive results in changes in fibril organisation including a reduced roughness and a more structured surface on which the fibrils are more clearly resolved. Concerning the AFM measurements, the surface will have an organisation that is influenced by the nature of the interface, where it is clear from the images that the fibrils arrange parallel to the surface. The fibrils are also resolved in phases images which can provide information about their organisation over larger areas (Figs 5e and f). As in the topographic images the fibrils are more easily resolved in the ACPCA film and, while the separation of fibrils is approximately the same in each image, the ACPCA film shows a feature around which fibrils with variable lengths diverge – see the region marked with an arrow in the topographic image, Fig. 5a. These features are also evident in Fig. 5e and have a characteristic separation in the range of 100s nm, similar to the fibril lengths. These features, which have an appearance reminiscent of topological defects, are not resolved in the absence of the chiral additive (Fig. 5f). The phase signal from the AFM is usually interpreted as being sensitive to variations in composition, adhesion, friction, viscoelasticity properties, particularly for soft materials, that modify the damping of the oscillating cantilever.[23]

Supplementary Figure 16 2D-FFT of the AFM phase image of the annealed F8BT ACPCA films

2D-FFT of the AFM phase image of the annealed F8BT-based ACPCA film (on the left) and Lorentzian fitting performed on the highlighted peaks of the profile (on the right).

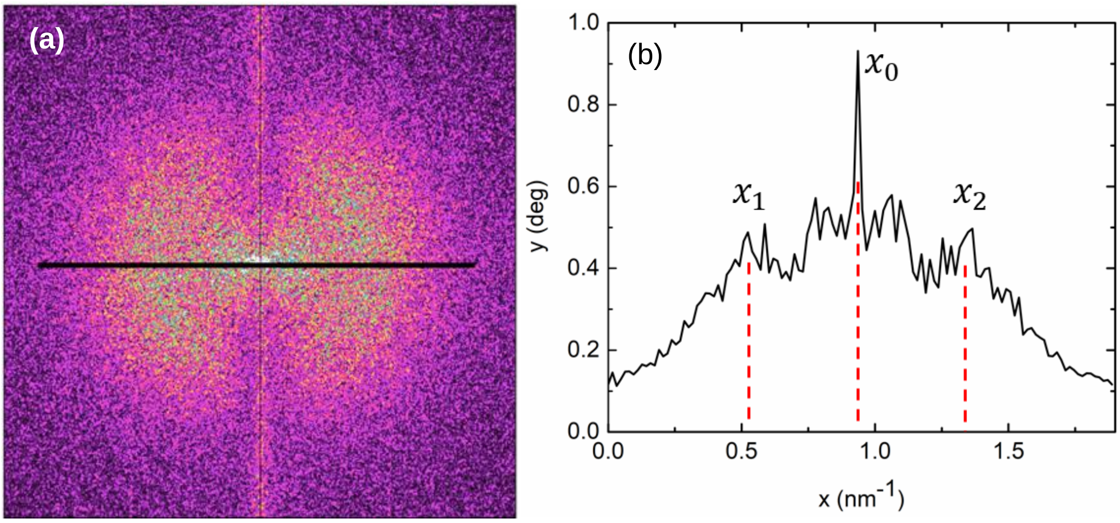

A 2D-FFT of the image has been performed (a) and a Lorentzian fitting (b) was then used to calculate the wave vector of the peaks associated showing a periodicity in the lattice of about ~ 25 nm that corresponds to the average width of the features visible in the phase image at the micro-scale already.

$x_0 = 98.99\ \mu m^{-1} \pm 0.\ 40\ \mu m^{-1}$

$x_1 = 58.33\ \mu m^{-1} \pm 0.78\ \mu m^{-1};\ 1/(x_1 - x_0) = (24.6 \pm 0.7)\ nm$

$x_2 = 139.66\ \mu m^{-1} \pm 0.75\ \mu m^{-1}; 1/(x_2 - x_0) = (24.6 \pm 0.7)\ nm$

Supplementary Figure 17 Optical micrographs

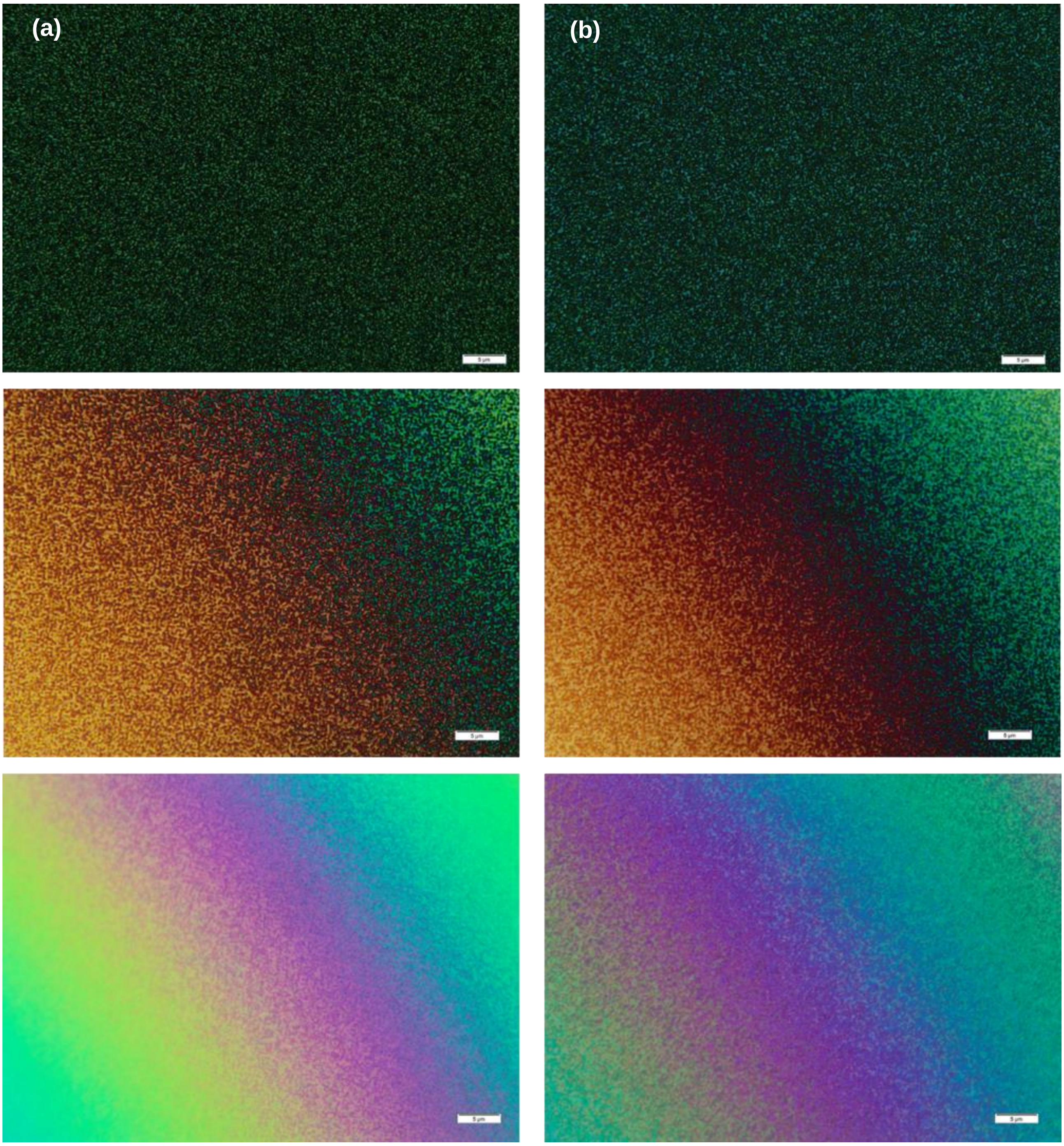

Optical micrographs of thermally annealed F8BT (a) and F8BT with 10 wt% aza[6]H (b). The length on the scale bar is 5 μm. Top row transmission with crossed polarisers, middle row as top row and Berek compensator at 50 degrees. Bottom row, reflection micrograph under otherwise identical conditions to the middle row.

Supplementary Figure 18 *In situ* circular dichroism spectra of ACPCA thin films

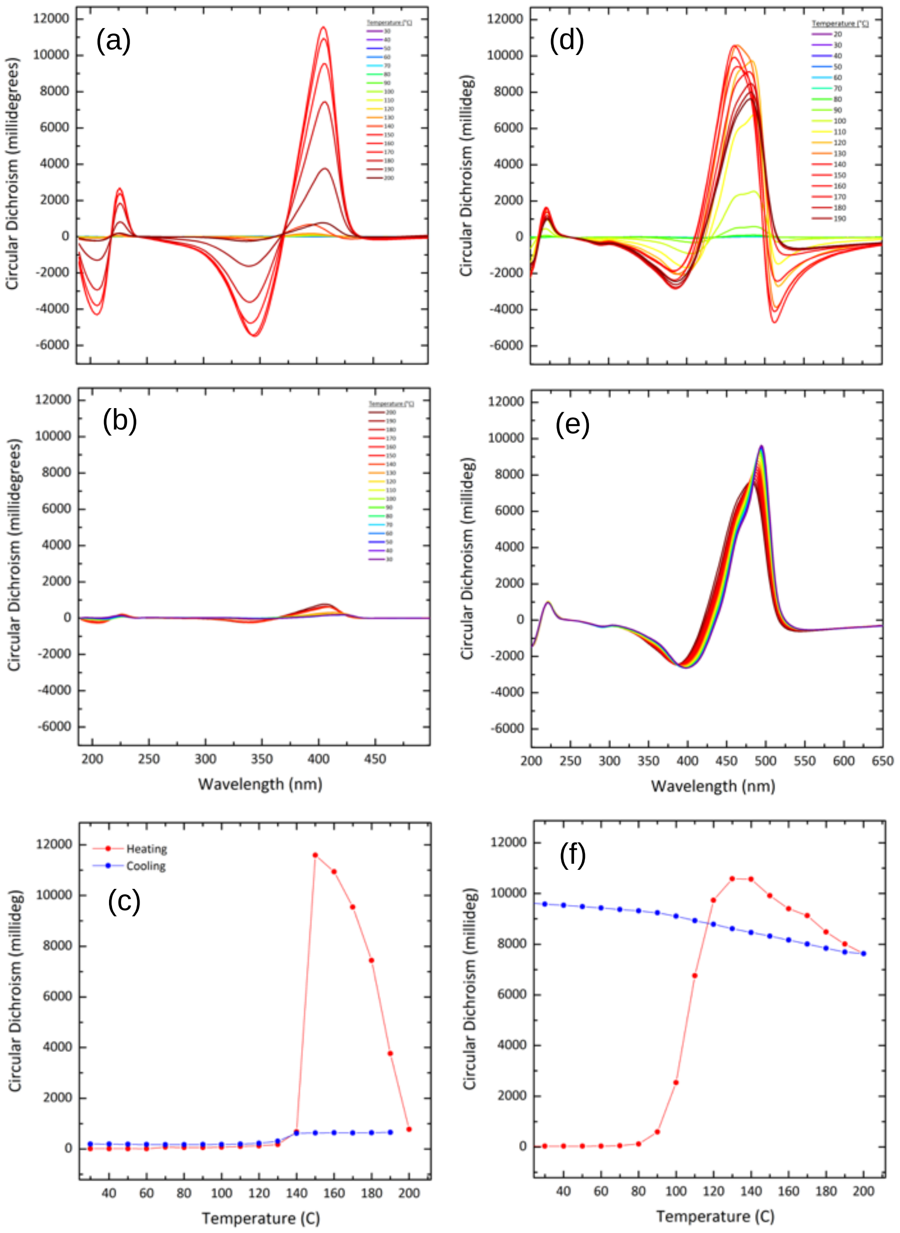

In situ circular dichroism spectra for PFO and F8T2:aza[P] thin films as a function of temperature during (a,d) heating and (b, e) cooling. (c, f) the maximum CD of the lowest energy transition from (a, b, d and e) for each polymer as a function of temperature.

Supplementary Figure 19 Extracted dissymmetry for ACPCA and CSCP thin films

Extracted dissymmetry (g-factor) for ACPCA (F8BT: aza[6]H) (a) and CSCP (b) thin films. These data are extracted from the *in situ* CD spectra in Figure 5.

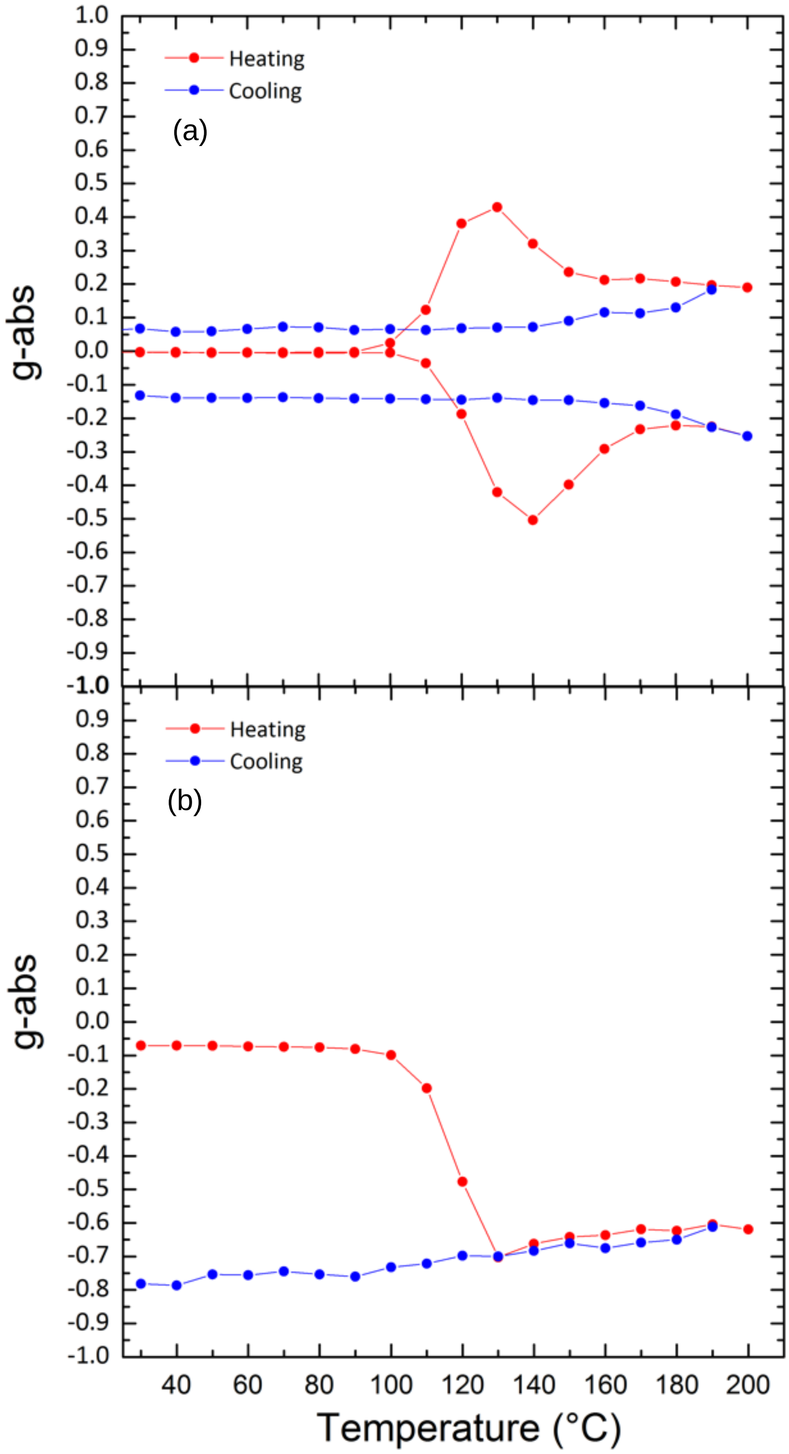

Supplementary Discussion 4 Results of *in situ* CD

To extend our understanding of the phase behaviour of ACPCA and CSCP thin films, we performed *in situ* CD measurements. In the case of F8T2, the chiroptical response recovers upon cooling (Supplementary Figure 18). For F8BT and PFO, the hysteresis of the chiroptical response (Figure 7 (b), Supplementary Figure 18) is not observed during a second cycle of heating and cooling (Supplementary Figure 20); where the increase and subsequent decrease of CD is fully reversible, but the magnitude is restricted to the CD recorded after the first cycle. In this case, the magnitude of the CD signal is maintained as the thin films are cooled to room temperature and is retained in subsequent heating/cooling cycles (Supplementary Figure 20). For the ACPCA thin films the chiroptical response increases dramatically if the films are rapidly cooled to room temperature (Supplementary Figures 20 – 22), as opposed to when they are cooled slowly (Figure 7, Supplementary Figure 18). This suggests that for a strong chiroptical effect in such blend materials it is essential to kinetically trap the chiral film structure formed around the glass transition temperature. The same is not true for the CSCP thin films, where slow cooling barely changes the chiroptical response (Figure 2, 7, S23). This demonstrates that the structure induced by the chiral sidechains is more persistent over repeated heating-cooling cycles.

Supplementary Figure 20 *In situ* circular dichroism spectra of F8BT:aza[P] thin films

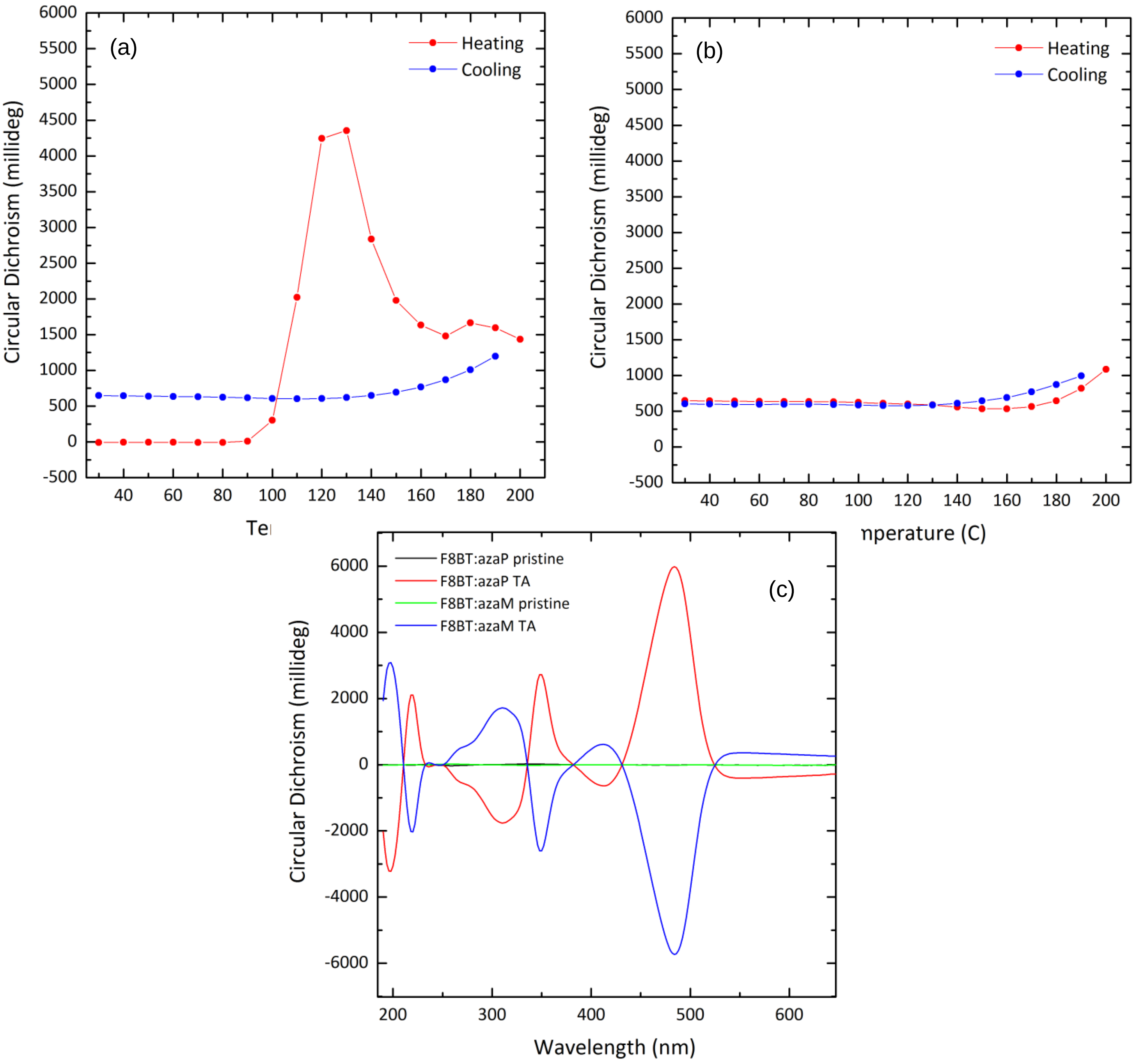

Maximum CD of F8BT:aza[*P*] extracted from *in situ* circular dichroism spectra during the first (a) and second (b) heating/cooling cycle, and (c) CD spectra recorded before and after annealing/ fast-cooling for F8BT:aza[*M*] and aza[*P*].

Supplementary Figure 21 *In situ* circular dichroism spectra of F8T2:aza[P] thin films

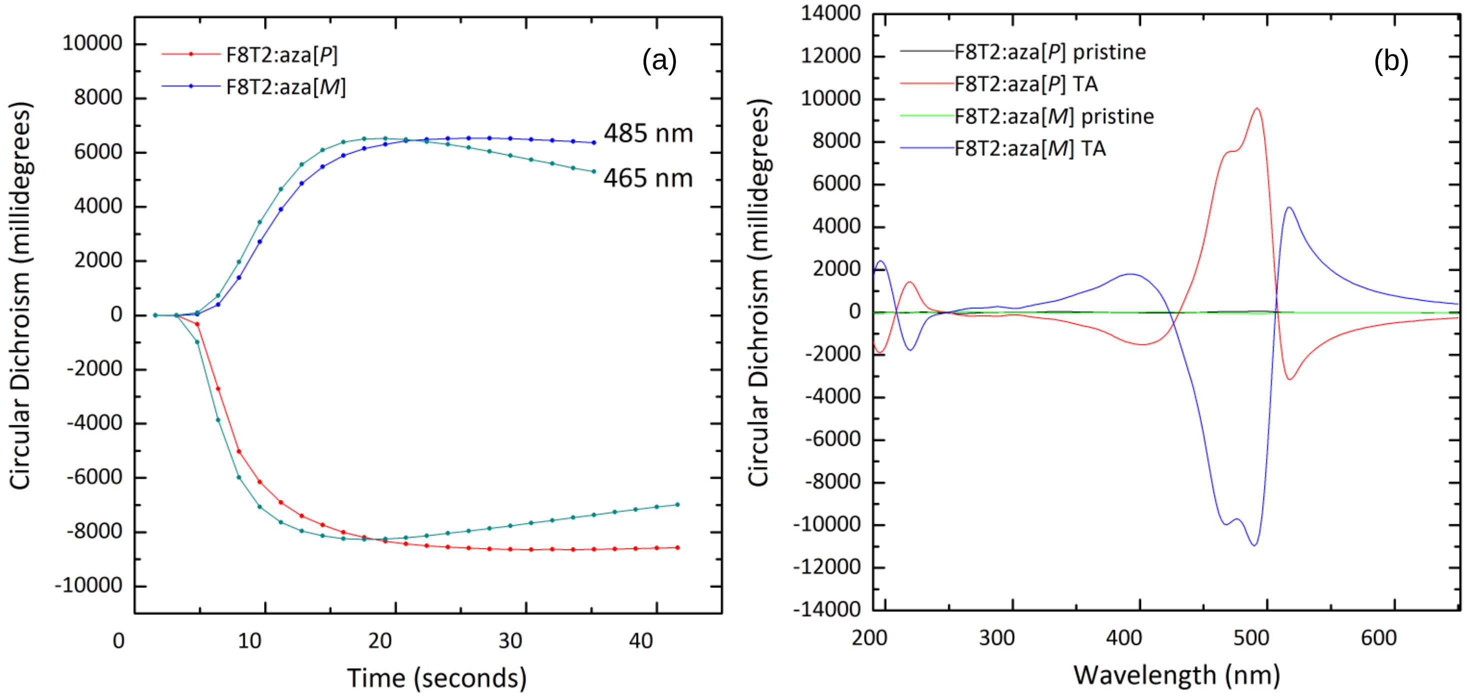

CD recorded at λ = 485 nm for F8T2:aza[6]H thin films held at 140 °C (b) CD spectra recorded before and after annealing for F8T2:aza[*M*] and aza[*P*].

Supplementary Figure 22 *In situ* circular dichroism spectra of PFO:aza[P] thin films

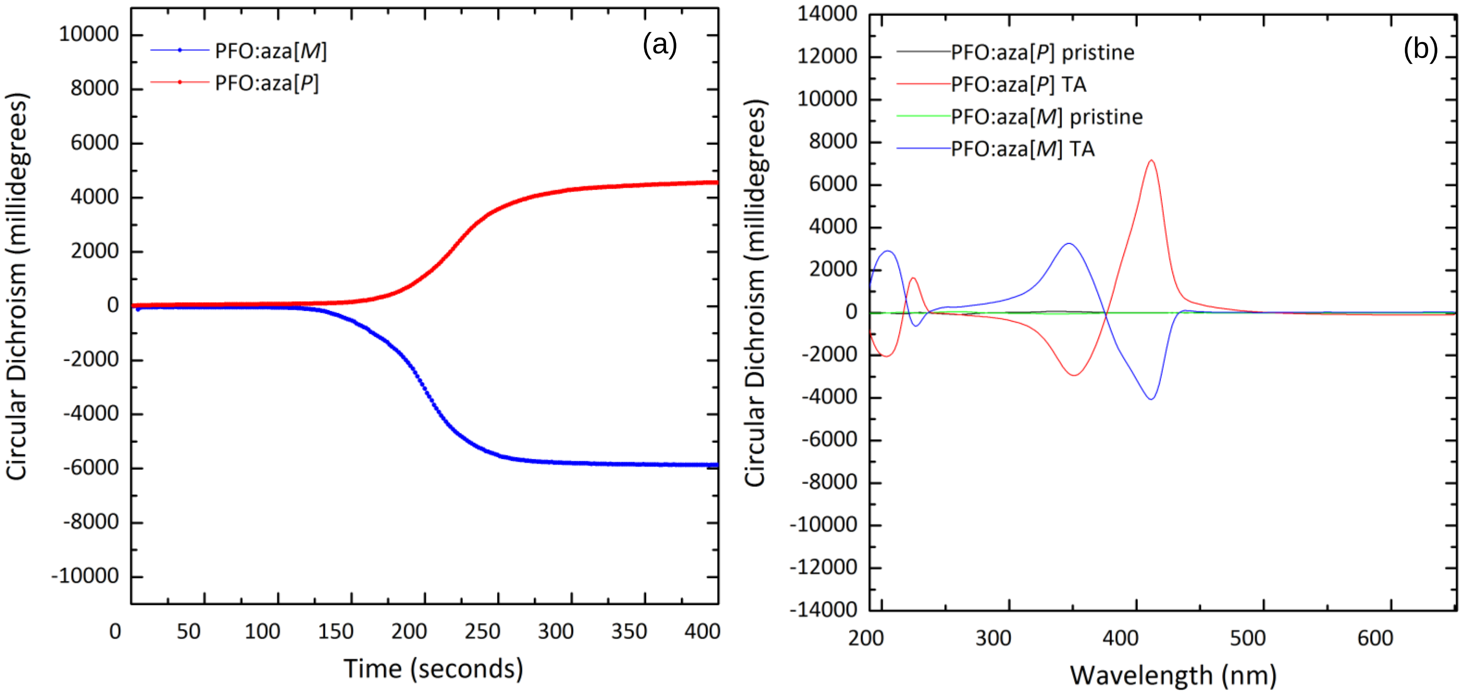

CD recorded at λ = 405 nm for PFO:aza[6]H thin films held at 160 °C (b) CD spectra recorded before and after annealing for PFO:aza[*M*] and aza[*P*].

Supplementary Figure 23 Temperature-dependent *in situ* circular dichroism spectra of CSCP thin films.

In situ circular dichroism spectra for cPFBT during heating and cooling and the maximum CD of the lowest energy transition during the first (a) and second (b) heating/cooling cycle.

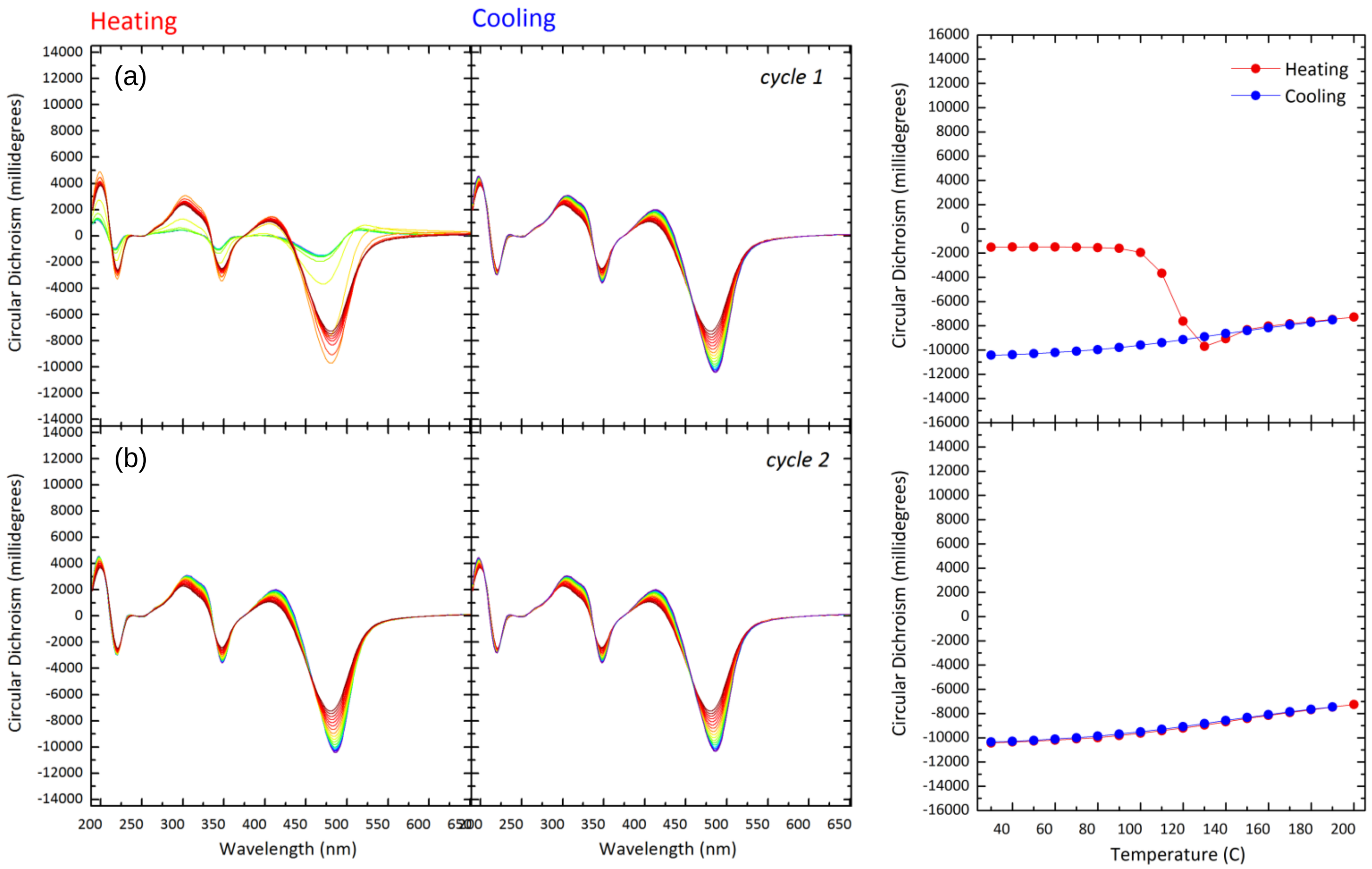

In situ circular dichroism spectra for cPFO during heating and the maximum CD of the lowest energy transition during the first (top row) and second (bottom row) heating/cooling cycle.

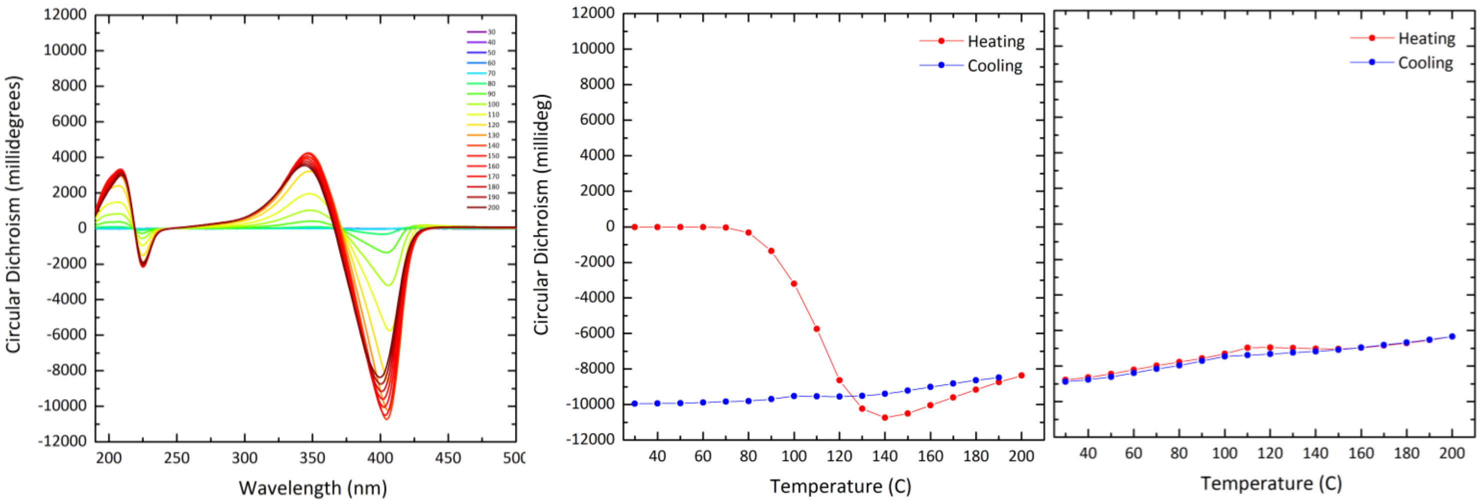

Supplementary Figure 24: Time-dependent *in situ* circular dichroism spectra of CSCP thin films

CD recorded at λ = 405 nm for cPFO and λ = 485 nm for cF8BT thin films held at 140 °C (b) CD spectra recorded before and after annealing for (a) cPFO and (b) cPFBT.

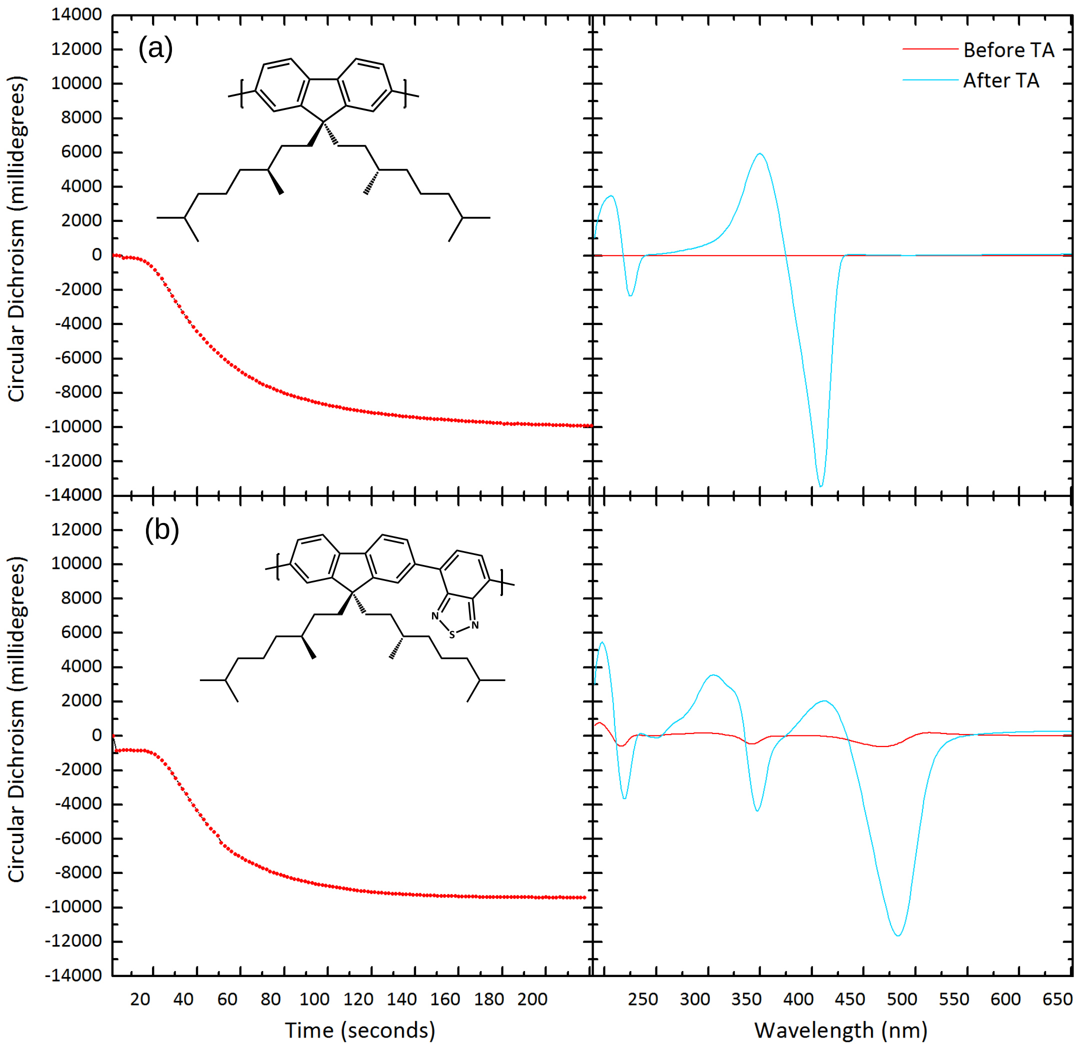

Supplementary Discussion 5: *in situ* CD measurements

Using the temperature at which we obtain the strongest CD ($T_{CD\ Max}$, extracted from the *in situ* temperature dependent CD measurements described above, Figure 7, Supplementary Figures 17 – 21), we investigated the annealing time required to induce the strongest chiroptical response in 120 nm thick films (Figure 7, Supplementary Figures 19 – S21, Supplementary Figure 23). The chiroptical effect is induced very quickly; within 300 seconds of being held at $T_{CD\ Max}$ for PFO, 20 seconds for F8T2, 800 seconds for F8BT and 180 seconds for both the CSCP thin films.

Of the ACPCA thin films considered in this study, the strongest chiroptical effect at room temperature is observed in the F8T2 : aza[6]H films, where CD ≈ 19,500 mdeg and represents the highest reported chiroptical effect in polymer thin films, and the weakest in PFO : aza[6]H films, where CD ≈ 3,100 mdeg (Figure 2). This may be due to the relative flexibility of the thiophene units, reduced density of sidechains resulting in more homogeneous conformation of polymer backbones, or differences in the chemical composition of the polymers.[24–26] In the case of the ACPCA thin films, *in situ* CD (Figure 5) indicates that below 200 °C there are two phases with strong chiroptical properties within F8BT and PFO, one kinetic chiral structure that forms at $T_{CD\ Max}$ with high CD, but does not persist upon elevating the temperature or repeat heating/cooling cycles. The other structure seems more thermodynamically robust, with lower CD, that forms when $T > T_{CD\ Max}$ and persists. In contrast, for F8T2 only one high intensity CD chiral phase forms, which is thermodynamically stable up to 200 °C, and is stable to further heating cycles. Regardless, the mirror symmetry of the temperature- and time-dependent CD spectra for the ACPCA thin films with [*P*] and the [*M*] enantiomers of the chiral additive (Figure 7), and absence of any aza[6]H signature in the chiroptical response, indicates that the aza[6]H acts as a chiral seed to template the handedness of the polymer chiral structure. For the CSCPs, there exists only one high intensity CD stable chiral phase below 200 °C that forms when T = 140 °C (Supplementary Figure 16). These results indicate that both approaches – chiral additive versus chiral sidechain – allow for a complementary means to achieve chiral polymer films with very large CD due to natural optical activity.

Supplementary Figure 25 Thickness dependence measurements of g_{abs}

<table>
<tr><th></th><th></th><th>ACPCA</th><th>CSCP</th></tr>
<tr><td rowspan="3">Non-aligned</td><td>F8T2</td><td>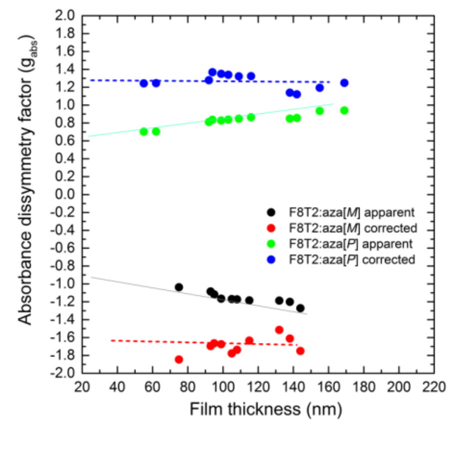
</td><td></td></tr>
<tr><td>F8BT</td><td>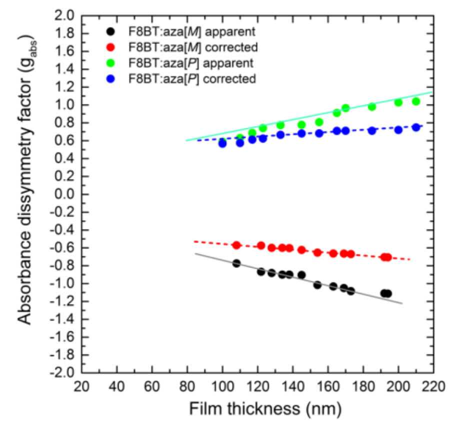
</td><td>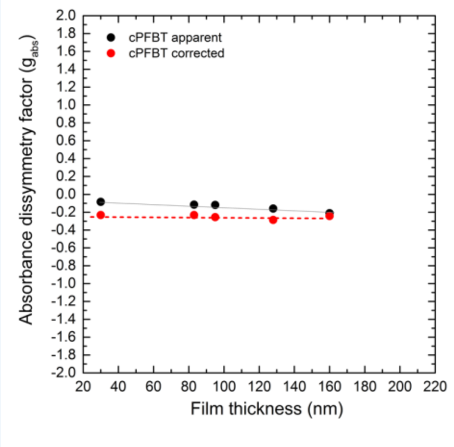
</td></tr>
<tr><td>PFO</td><td>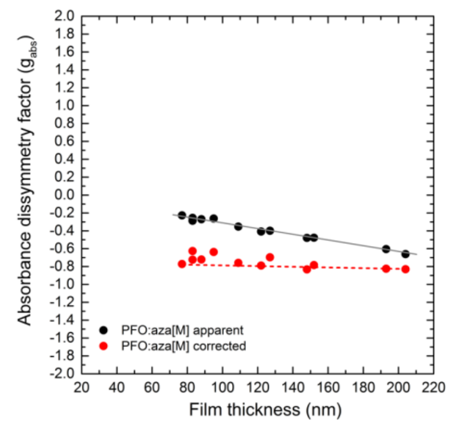
</td><td>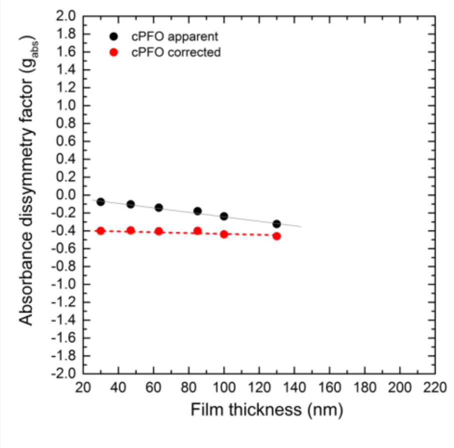
</td></tr>
<tr><td>Aligned</td><td>F8BT</td><td>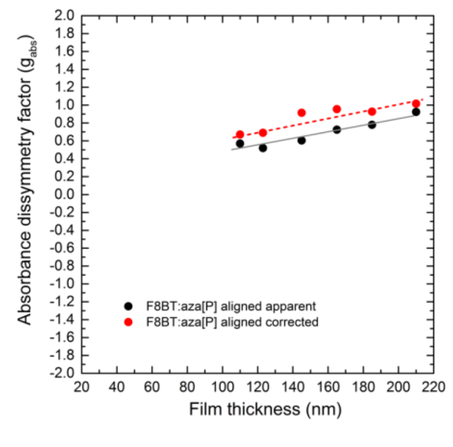
</td><td>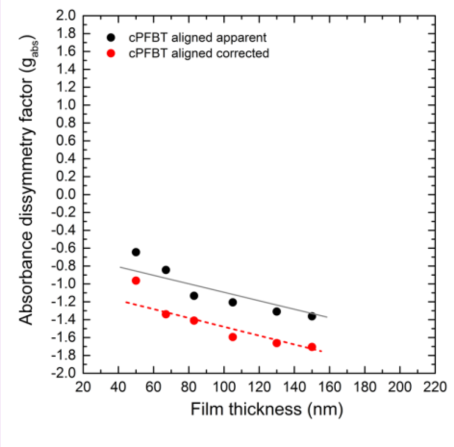
</td></tr>
</table>

Supplementary Discussion 6: Thickness dependence measurements of g_{abs}

To quantitively assess the optical activity of aligned and non-aligned ACPCA and CSCP thin films annealed at $T_{CD\ Max}$ we calculated the dissymmetry factor, g_{abs}, by normalising the CD (ΔA) to the unpolarised absorbance of the sample. To better understand the origins of this chiroptical response, we controlled the spin-coating speed to fabricate a series of thin films with different thicknesses. In such films, the competition of different aggregation pathways may result in the formation of various polymorphs in principle. However, the variable thickness ACPCA films considered here suggest that this is not the case for these materials, with similar shaped CD profiles for thick and thin films and no evidence of different polymorphs in AFM or cross-polarised optical microscopy images.[21]

At first glance, the g-factors of all systems (aligned and not-aligned, ACPCA and CSCP) appear to increase as a function of film thickness (i.e. thicker films achieve higher g_{abs} than thin films). To accommodate for reflection losses at the interfaces, we followed the simple protocol introduced by Schiek *et al.*[7] Once reflection losses are accounted for, the non-aligned thin films have a thickness independent g_{abs}, whereas aligned thin films show a g_{abs} that increases linearly as a function of film thickness. Clearly, this thickness-dependent dissymmetry of aligned systems (i.e. where structural chirality dominates) should tend to zero for very thin films, but in the cases considered here, we believe both natural optical activity and structural chirality contribute to the measured chiroptical response.

We should note that the large *g*-factors observed for these systems contrast the small effects typically seen for small molecules. In the case of small molecules, the *g*-factor can be calculated from[27];

$$g = 4\frac{R}{D} = 4 \times \frac{Im\ \boldsymbol{\mu}_{ij} \cdot \mathbf{m}_{ij}}{|\boldsymbol{\mu}_{ij}|^2 + |\boldsymbol{m}_{ij}|^2} \quad \text{Supplementary Equation 10}$$

Where $\boldsymbol{\mu}$ is the electric transition dipole moment, $\mathbf{m}$ the magnetic transition dipole moment, R the rotational strength and D the dipole strength of an electronic transition from i-j. Based on Supplementary Equation 10 and the large $\boldsymbol{\mu}$s of fluorene polymers, *g*-factors > 1 would require unrealistically large magnetic transition dipole moments. However, the well-known expression for *g*-factor (i) assumes isolated, non-interacting chromophores, (ii) assumes molecules that are much smaller than the wavelength of light and (iii) neglects

anisotropic effects (i.e. it does not consider quadrupole terms or higher order coupling). For anisotropic systems such as the polymer-based thin films considered here, a more sophisticated expression is required, and the coupling of transition dipole moments on nearby polymer chains must be considered.[28]

Supplementary References

1. Jo, J., Chi, C., Höger, S., Wegner, G. & Yoon, D. Y. Synthesis and Characterization of Monodisperse Oligofluorenes. *Chem. - A Eur. J.* **10**, 2681–2688 (2004).

2. Abbel, R. *et al.* Side-Chain Degradation of Ultrapure π-Conjugated Oligomers: Implications for Organic Electronics. *Adv. Mater.* **21**, 597–602 (2009).

3. Kosco, J. *et al.* The Effect of Residual Palladium Catalyst Contamination on the Photocatalytic Hydrogen Evolution Activity of Conjugated Polymers. *Adv. Energy Mater.* **8**, 1802181 (2018).

4. Kulkarni, C. *et al.* Molecular Design Principles for Achieving Strong Chiroptical Properties of Fluorene Copolymers in Thin Films. *Chem. Mater.* **31**, 6633–6641 (2019).

5. Jung, J., Lee, D., Kim, J. & Yu, C. Circularly polarized electroluminescence by controlling the emission zone in a twisted mesogenic conjugate polymer. *J. Mater. Chem. C* **6**, 726–730 (2018).

6. Schulz, M. *et al.* Chiral Excitonic Organic Photodiodes for Direct Detection of Circular Polarized Light. *Adv. Funct. Mater.* **29**, 1900684 (2019).

7. Schulz, M. *et al.* Giant intrinsic circular dichroism of prolinol-derived squaraine thin films. *Nat. Commun.* **9**, 2413 (2018).

8. Kulkarni, C., Meskers, S. C. J., Palmans, A. R. A. & Meijer, E. W. Amplifying Chiroptical Properties of Conjugated Polymer Thin-Film Using an Achiral Additive. *Macromolecules* **51**, 5883–5890 (2018).

9. Di Nuzzo, D. *et al.* High Circular Polarization of Electroluminescence Achieved via Self-Assembly of a Light-Emitting Chiral Conjugated Polymer into Multidomain Cholesteric Films. *ACS Nano* **11**, 12713–12722 (2017).

10. Albano, G., Lissia, M., Pescitelli, G., Aronica, L. A. & Di Bari, L. Chiroptical response inversion upon sample flipping in thin films of a chiral benzo[1,2-b:4,5-b′]dithiophene-based oligothiophene. *Mater. Chem. Front.* **1**, 2047–2056 (2017).

11. Albano, G. *et al.* Outstanding Chiroptical Features of Thin Films of Chiral Oligothiophenes. *ChemNanoMat* **4**, 1059–1070 (2018).

12. Kulkarni, C., Di Nuzzo, D., Meijer, E. W. & Meskers, S. C. J. Pitch and Handedness of the Cholesteric Order in Films of a Chiral Alternating Fluorene Copolymer. *J. Phys. Chem. B* **121**, 11520–11527 (2017).

13. Shen, Z., Wang, T., Shi, L., Tang, Z. & Liu, M. Strong circularly polarized luminescence from the supramolecular gels of an achiral gelator: tunable intensity and handedness. *Chem. Sci.* **6**, 4267–4272 (2015).

14. Lakhwani, G. & Meskers, S. C. J. Insights from chiral polyfluorene on the unification of molecular exciton and cholesteric liquid crystal theories for chiroptical phenomena. *J. Phys. Chem. A* **116**, 1121–1128 (2012).

15. Lakhwani, G., Koeckelberghs, G., Meskers, S. C. J. & Janssen, R. A. J. The chiroptical properties of chiral substituted poly[3-((3S)-3,7-dimethyloctyl)thiophene] as a function of film thickness. *Chem. Phys. Lett.* **437**, 193–197 (2007).

16. Craig, M. R., Jonkheijm, P., Meskers, S. C. J., Schenning, A. P. H. J. & Meijer, E. W. The Chiroptical Properties of a Thermally Annealed Film of Chiral Substituted Polyfluorene Depend on Film

Thickness. *Adv. Mater.* **15**, 1435–1438 (2003).

17. Geng, Y. *et al.* Synthesis, characterization, and optical properties of monodisperse chiral oligofluorenes. *J. Am. Chem. Soc.* **124**, 8337–8347 (2002).

18. Geng, Y. *et al.* Origin of Strong Chiroptical Activities in Films of Nonafluorenes with a Varying Extent of Pendant Chirality. *J. Am. Chem. Soc.* **125**, 14032–14038 (2003).

19. Wilson, J. N. *et al.* Chiroptical Properties of Poly(p - phenyleneethynylene) Copolymers in Thin Films: Large g -Values. *J. Am. Chem. Soc.* **124**, 6830–6831 (2002).

20. Yang, Y., Da Costa, R. C., Smilgies, D. M., Campbell, A. J. & Fuchter, M. J. Induction of circularly polarized electroluminescence from an achiral light-emitting polymer via a chiral small-molecule dopant. *Adv. Mater.* **25**, 2624–2628 (2013).

21. Wan, L. *et al.* Inverting the Handedness of Circularly Polarized Luminescence from Light-Emitting Polymers Using Film Thickness. *ACS Nano* **13**, 8099–8105 (2019).

22. Donley, C. L. *et al.* Effects of Packing Structure on the Optoelectronic and Charge Transport Properties in Poly(9,9-di- n -octylfluorene- alt -benzothiadiazole). *J. Am. Chem. Soc.* **127**, 12890–12899 (2005).

23. García, R. *Amplitude Modulation Atomic Force Microscopy*. *Amplitude Modulation Atomic Force Microscopy* (Wiley-VCH Verlag GmbH & Co. KGaA, 2010).

24. Barbarella, G., Melucci, M. & Sotgiu, G. The Versatile Thiophene: An Overview of Recent Research on Thiophene-Based Materials. *Adv. Mater.* **17**, 1581–1593 (2005).

25. Di Maria, F. *et al.* A Successful Chemical Strategy To Induce Oligothiophene Self-Assembly into Fibers with Tunable Shape and Function. *J. Am. Chem. Soc.* **133**, 8654–8661 (2011).

26. Wang, P. *et al.* Insights into Magneto-Optics of Helical Conjugated Polymers. *J. Am. Chem. Soc.* **140**, 6501–6508 (2018).

27. Blok, P. M. L. & Dekkers, H. P. J. M. Discrimination between $3\pi\pi^*$ and $3n\pi^*$ states in organic molecules by circular polarization of phosphorescence. *Chem. Phys. Lett.* **161**, 188–194 (1989).

28. Snir, J. & Schellman, J. Optical activity of oriented helixes. Quadrupole contributions. *J. Phys. Chem.* **77**, 1653–1661 (1973).
